# Polaritonic Coupled Cluster Theory for Unpolarized Cavities Exploiting Point-Group Symmetry

**DOI:** 10.1021/acs.jctc.5c01343

**Published:** 2026-06-02

**Authors:** Laurenz Monzel, Stella Stopkowicz

**Affiliations:** † 200989 Saarland University, Department of Chemistry, Physical and Theoretical Chemistry, Campus B2.2, 66123 Saarbrücken, Germany; ‡ Hylleraas Centre for Quantum Molecular Sciences, Department of Chemistry, 9379 University of Oslo, P.O. Box 1033, Blindern, N-0315 Oslo, Norway

## Abstract

We introduce a generalization of the quantum electrodynamic coupled cluster (QED-CC) wave function ansatz, to describe the strongly coupled light-matter system in an unpolarized optical Fabry-Pérot cavity. This is achieved by explicitly treating two cavity modes in our calculation with perpendicular polarizations and demonstrate that this ansatz preserves the symmetry of an unpolarized cavity. Furthermore, exploiting point-group symmetry enables the assignment of polaritonic excited states as well as their targeted calculation. Using our implementation, the aromatic species benzene, fluorobenzene, and azulene are investigated. We demonstrate that molecules in unpolarized cavities have a complicated excited-state landscapes with a plethora of avoided-crossings. We compare the results for a cavity with a single polarization to those of an unpolarized cavity described by two perpendicular polarization vectors using the excited states of the H_2_ molecule as an example.

## Graphical abstract

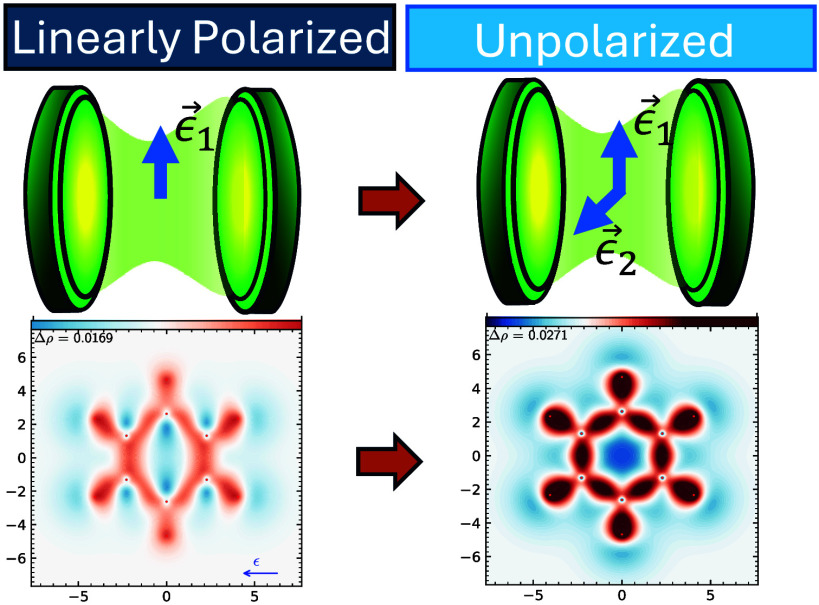

## 1 Introduction

Manipulating molecular properties in a targeted manner is a central objective of chemistry. Historically, methods are sought that have the most specific influence while being easy to implement experimentally. One possibility that has received significant attention in recent years, is the strong coupling to an electromagnetic field.^1–5^ By placing molecules in cavities, light-matter hybrids can be formed, which can be manipulated by carefully choosing the frequency and coupling strength of the cavity.^6,7^ The strong coupling leads to the mixing of electronic and photonic degrees of freedom, forming so-called polaritons,^1,8,9^ in cases where the mode volume of the cavity is small enough and where the loss of photons to the continuum is sufficiently suppressed.^10^ Another route to achieve strong coupling is by increasing the number of molecules, coupled collectively to the cavity.^11^ This enables the control of the matter states at room temperature with a relatively cheap experimental setup.^12^ Further, as in the long-wavelength limit the cavity-mediated interaction between particles is not decaying with their distance, collective phenomena occur which are currently heavily investigated both experimentally^1,3,13^ and theoretically.^7,14–16^ This allows to reach the strong coupling regime also by coupling sufficiently many molecules to the cavity field.^14^

Regarding matter systems, a plethora of theoretical methods have been formulated which aim to solve for the many-particle wave function of the system. In order to also include the cavity, these methods have been generalized to also include effects emerging from quantum electrodynamics (QED). Regarding optical cavities which strongly couple to electronic transitions, QED Hartree–Fock theory (QED-HF),^17,18^ density functional theory (QEDFT),^19–23^ configuration interaction theory (QED-CI),^24,25^ perturbation theory (QED-PT),^26,27^ multireference methods (QED-CASSCF)^28^ and various flavors of coupled-cluster theory (QED-CC)^17,24,29,30^ have been formulated. Compared to model Hamiltonians as the Dicke-, Jaynes- or Tavis-Cummings models,^31,32^ the aforementioned methods have the advantage that they allow for the relaxation of the electronic structure and treat electrons and photons on the same footing. In particular, it is mostly QED-CC theory and its variants which have been proven to provide highly accurate results. Recent milestones in polaritonic CC theory were the initial formulation,^17,24^ treating circularly polarized cavities,^33^ and the implementation of molecular gradients.^34^

Yet, QED-CC theory has only been formulated for single polarizations, both for linearly and circularly polarized cavities.^17,24,33^ By going beyond the single-mode approximation and explicitly treating two perpendicularly polarized cavity modes, we take a step toward a more realistic setting and enabling the description of unpolarized cavities as often used experimentally, such as, for example, the Fabry-Pérot cavity.^35^

In this paper, we present a CC implementation for molecules in unpolarized cavities in the dipole-approximation^36,37^ by explicitly treating two perpendicularly polarized modes and thereby moving beyond the single-mode approximation toward a more realistic representation of experimental cavity settings. Further steps toward a fully realistic description would include, for instance, polarization dependent mode volumes, dissipative losses, and boundary-induced modifications of the field, which are beyond the scope of the present work. We present how point-group symmetry can be applied for molecules in cavities and its exploitation in the speed-up of the respective calculations as well as in the interpretation of polaritonic states. An efficient implementation of point-group symmetry in CC calculations based on the direct-product decomposition^38^ is adapted here for the use in our QED-CC implementation.

First, in Section 2 the underlying theory of a strongly coupled light-matter system in an unpolarized Fabry-Pérot cavity is presented. We present further how the direct-product decomposition is used for the evaluation of the nonlinear coupled cluster (CC) equations.^39^ Section 3 presents exemplary calculations on molecules to study the effects of an unpolarized cavity for the ground and excited states. In (Sections 3.1–3.3), ground-state calculations on the aromatic species benzene, fluorobenzene, as well as azulene are presented. In (Sections 3.4 and 3.5) we present excited state calculations for unpolarized cavities with the help of EOM-CC and compare the coupling mechanism for a linearly and unpolarized cavity.

## 2 Theory

We will follow the index convention that *a*, *b*, *c*,··· refer to virtual orbitals, *i*, *j*, *k*,··· to occupied orbitals, and *p*, *q*, *r*,··· are generic orbital indices. Photonic modes are denoted with greek letters as α, β, γ,···. Further, we will express all operators and quantities in terms of atomic units

$$\hbar =e=m_{e}=1$$

Throughout, we stay in the polaritonic energy surface partitioning where the electron-photon wave function is described in the field of the parametrically treated nuclei.^7^

### 2.1 The Dipole Hamiltonian

In the dipole approximation it is assumed that the wavelength of the electromagnetic (EM) field exceeds the dimensions of the molecule.^7,21,36,37^ The interacting system of electrons and the EM field is then derived from the Pauli-Fierz Hamiltonian in the length gauge which is in the coherent-state basis given by^7^

$$Ĥ={\hat{\mathrm{H̃}}}_{\mathrm{el}}-\mathrm{D̂}\cdot \hat{\mathrm{d̃}}+{Ĥ}_{\mathrm{cav}}$$

This Hamiltonian describes the electrons interacting with homogeneously oscillating electric fields, where the light-matter interaction is captured by the product of the phase-shifted homogeneous displacement field ***D* ^** and the dipole-fluctuation operator ***d* ~**^. The phase-shifted displacement field ***D* ^** in the length gauge reads

$$\mathrm{D̂}=\sum_{\nu =1,2}\sum_{\alpha }^{N_{\mathrm{cav}}}\sqrt{\frac{\lambda_{\alpha ,\nu }^{2}\omega_{\alpha }}{2}}\epsilon_{\alpha ,\nu }\left({\mathrm{b̂}}_{\alpha ,\nu }^{†}+{\mathrm{b̂}}_{\alpha ,\nu }\right)$$

where each mode is characterized by its frequency ω_α_, coupling strength λ_α,*ν*_ and field polarizations **ϵ**_α,*ν*_. The index *ν* runs over two real-valued orthogonal field polarizations **ϵ**_α,1_ and **ϵ**_α,2_, which are aligned perpendicular to the principal axis of the cavity.

The photon-creation and annihilation operators are denoted by *b̂*_α_^†^ and *b̂*_α_. In the coherent-state basis, ***d* ~**^ is the dipole operator in normal order

$$\hat{\mathrm{d̃}}=\mathrm{d̂}-\left\langle d\right\rangle$$

with the dipole operator ***d* ^**.^17,27,40^ The operator ***d* ~**^ describes the dipole fluctuation relative to the Fermi vacuum.^41^ The coherent-state basis guarantees the translational invariance of the Hamiltonian.^17^ Typically, a Hartree–Fock (HF) reference determinant is chosen for the Fermi vacuum, while photons are described in a coherent-state ansatz. An alternative ansatz for the reference wave function could possibly be a strong-coupling QED Hartree–Fock (SC-QED-HF)^18^ reference, but, so far this ansatz has only been exploited in a perturbation-theory framework.^27^ The electronic Hamiltonian *Ĥ*_el_ includes the kinetic energy as well as the electrostatic electron–nuclei and electron–electron interactions

1 $${Ĥ}_{\mathrm{el}}={\mathrm{T̂}}_{e}+{\mathrm{V̂}}_{\mathrm{eN}}+{Ŵ}_{\mathrm{ee}}$$

The cavity Hamiltonian (including the matter system) is given by

2 $${Ĥ}_{\mathrm{cav}}=\sum_{\nu =1,2}\sum_{\alpha }^{N_{\mathrm{cav}}}\left(\omega_{\alpha }{\mathrm{b̂}}_{\alpha ,\nu }^{†}{\mathrm{b̂}}_{\alpha ,\nu }+\frac{\lambda_{\alpha ,\nu }^{2}}{2}{\left(\epsilon_{\alpha ,\nu }\cdot \hat{\mathrm{d̃}}\right)}^{2}\right)$$

The first term corresponds to the excitation of the displacement modes and the second term is the dipole self-energy contribution. Note that the dipole self-energy term ensures that the Hamiltonian is bound from below. It hence enables the computation of polaritonic ground states.^7,21,37,42^ Furthermore, we note that the dipole self-energy is part of the photon field, even though it is technically a purely electronic operator. In calculations, the summation over the cavity modes α has to be truncated and only a few effective modes that lie close to electronic transitions are typically included. Which modes are selected can heavily influence the light-matter coupling and symmetry of the system, which will be discussed in the following sections. Note also, that when taking more modes into account, mass-renormalization effects increase.^43^ Here, we only take two modes (with the same frequency) into account and assume that mass-renormalization effects are negligible. A procedure how to include mass-renormalization effects for simple model systems can be found in ref 44.

### 2.2 Point-Group Symmetry for the Bare Cavity Hamiltonian

Our objective is to model a Fabry-Pérot cavity.^35^ Experimentally, such a cavity is formed by two mirrors, with the separation between them determining the frequencies ω_α_ of the EM modes supported within the cavity. First we discuss the bare cavity Hamiltonian (eq 2) in absence of the matter system, i.e.

3 $${Ĥ}_{\mathrm{bare}}=\sum_{\nu =1,2}\sum_{\alpha }\omega_{\alpha }{\mathrm{b̂}}_{\alpha ,\nu }^{†}{\mathrm{b̂}}_{\alpha ,\nu }$$

We note that the EM mode structure is altered in the presence of the matter system. The orientation of the cavity is defined by the wave vectors ***k***_α_, pointing in the direction of the mirrors. The wave vectors ***k***_α_ and frequencies ω_α_ are related by | ***k***_α_| = *cω*_α_, where *c* is the speed of light. For the bare cavity (eq 3, see also upper left corner of Figure 1), the symmetry corresponds to *D*_∞*h*_. Hence, the cavity Hamiltonian *Ĥ*_bare_ transforms symmetrically under symmetry operations within the *D*_∞*h*_ group. More concretely, *Ĥ*_bare_ transforms symmetrically under a *C*_∞_ rotation with respect to the principal axis of the cavity, under *C*_2_ rotations perpendicular to it, under vertical reflections σ_*v*_ containing the *C*_∞_ axis and under a horizontal reflection σ_*h*_ normal to the *C*_∞_ axis, etc.

**Figure 1:**
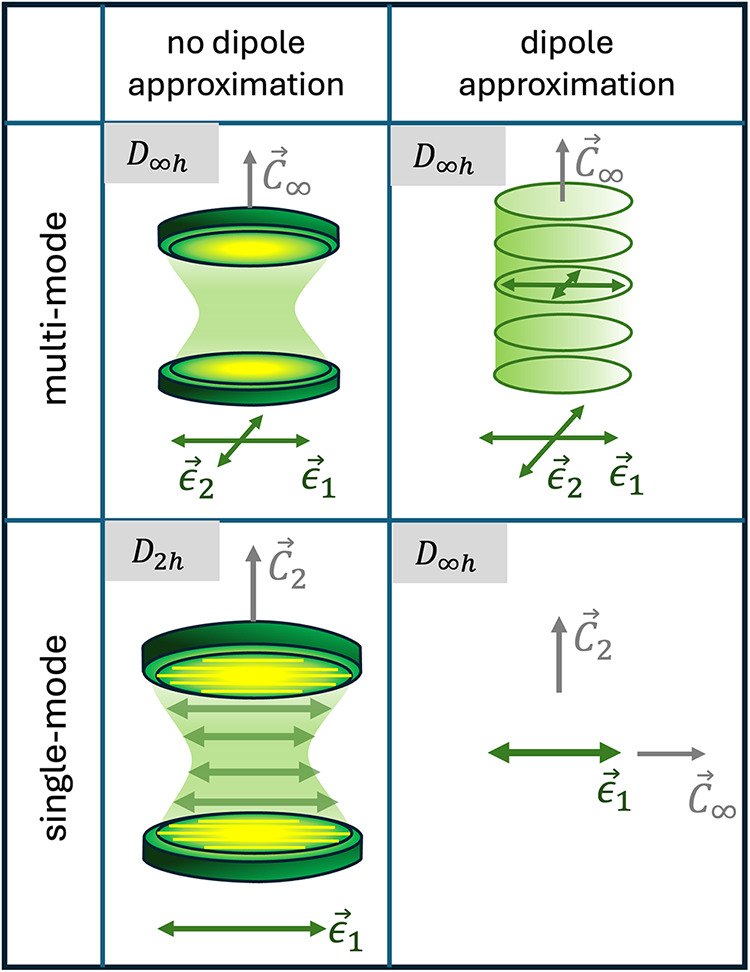
Cavity symmetry with and without the dipole approximation, respectively, in a multimode vs single-mode approximation. Polarization vectors are shown bidirectional to resemble the correct symmetry of the system.

The invariance of the Hamiltonian under symmetry operations within the group can be shown explicitly by introducing the corresponding symmetry operators. For a compact notation the polarization is made explicit by abbreviating the elementary operators as *b̂*_α,1_ = *b̂*_α_ and *b̂*_α,2_ = *b̅̂*_α_, with the corresponding perpendicular field polarization **ϵ**_α,1_ = **ϵ**_α_ and **ϵ**_α,2_ = **ϵ̅**_α_.

The bare cavity Hamiltonian (eq 3) can then be rewritten as

4 $${Ĥ}_{\mathrm{bare}}=\sum_{\alpha }^{N_{\mathrm{cav}}}\omega_{\alpha }\left({\mathrm{b̂}}_{\alpha }^{†}{\mathrm{b̂}}_{\alpha }+{\hat{\mathrm{b̅}}}_{\alpha }^{†}{\hat{\mathrm{b̅}}}_{\alpha }\right)$$

To show that the Hamiltonian is rotationally invariant, we introduce the associated operators for a rotation with respect to the principal axis of the cavity around an arbitrary angle θ

$${Ĉ}_{\theta }=\exp\left[\theta \sum_{\alpha }^{N_{\mathrm{cav}}}\left({\mathrm{b̂}}_{\alpha }^{†}{\hat{\mathrm{b̅}}}_{\alpha }-{\hat{\mathrm{b̅}}}_{\alpha }^{†}{\mathrm{b̂}}_{\alpha }\right)\right]$$

With this operator it can easily be verified that the cavity Hamiltonian *Ĥ*_cav_ in the form (eq 4) is invariant under *C*_∞_ rotations. Thereby, the elementary operators transform as

$$\begin{array}{l} {\mathrm{b̂}}_{\alpha }^{\prime }={Ĉ}_{\theta }^{†}{\mathrm{b̂}}_{\alpha }{Ĉ}_{\theta }={\mathrm{b̂}}_{\alpha }\cos\theta +{\hat{\mathrm{b̅}}}_{\alpha }\sin\theta \\ {\hat{\mathrm{b̅}}}_{\alpha }^{\prime }={Ĉ}_{\theta }^{†}{\hat{\mathrm{b̅}}}_{\alpha }{Ĉ}_{\theta }={\hat{\mathrm{b̅}}}_{\alpha }\cos\theta -{\mathrm{b̂}}_{\alpha }\sin\theta \end{array}$$

and analogously for the creation operator *b̂*_α_^†^. Hence, *Ĉ*_θ_^†^ *Ĥ*_bare_ *Ĉ*_θ_ = *Ĥ*_bare_. Also, the Hamiltonian in the form of eq 4 readily transforms symmetrically under σ_*v*_ reflections (or *C*_2_ rotations; *Ĉ*_2_(***n***) = σ̂_*v*_(***n***))

$${\mathrm{\sigma ̂}}_{v}\left(n\right)=\exp\left[i\pi \sum_{\alpha }^{N_{\mathrm{cav}}}\left(n_{2}^{2}{\mathrm{b̂}}_{\alpha }^{†}{\mathrm{b̂}}_{\alpha }+n_{1}^{2}{\hat{\mathrm{b̅}}}_{\alpha }^{†}{\hat{\mathrm{b̅}}}_{\alpha }-n_{1}n_{2}\left({\hat{\mathrm{b̅}}}_{\alpha }^{†}{\mathrm{b̂}}_{\alpha }+{\mathrm{b̂}}_{\alpha }^{†}{\hat{\mathrm{b̅}}}_{\alpha }\right)\right)\right]$$

The two-dimensional vector ***n*** = (*n*_1_, *n*_2_)^*T*^ is a unit vector in the **ϵ**_α_,**ϵ̅**_α_ plane and spans together with ***k*** the reflection plane of σ_*v*_. Likewise, for a *C*_2_(***n***) rotation it defines the rotation axis. The elementary operators transform as

$$\begin{array}{l} {\mathrm{b̂}}_{\alpha }^{\prime }={\mathrm{\sigma ̂}}_{v}^{†}{\mathrm{b̂}}_{\alpha }{\mathrm{\sigma ̂}}_{v}=\left(1-2n_{2}^{2}\right){\mathrm{b̂}}_{\alpha }+2n_{1}n_{2}{\hat{\mathrm{b̅}}}_{\alpha }\\ {\hat{\mathrm{b̅}}}_{\alpha }^{\prime }={\mathrm{\sigma ̂}}_{v}^{†}{\hat{\mathrm{b̅}}}_{\alpha }{\mathrm{\sigma ̂}}_{v}=\left(1-2n_{1}^{2}\right){\hat{\mathrm{b̅}}}_{\alpha }+2n_{1}n_{2}{\mathrm{b̂}}_{\alpha } \end{array}$$

The horizontal reflection σ_*h*_, is defined as a reflection normal to the ***k***_α_ vectors. It is hence located in a surface spanned by **ϵ**_α_ and **ϵ̅**_α_. In the dipole approximation, the electric field is homogeneous and oriented along the polarization vectors, so the horizontal reflection leaves the electric field invariant and therefore

$$\left[{\mathrm{\sigma ̂}}_{h},\mathrm{D̂}\right]=0$$

This, however, only holds in the dipole approximation, while for example a circularly polarized cavity changes the polarization under a σ̂_*h*_ operation as has been shown by Riso et al.^33^ As usual, higher symmetry operations as an inversion *i* or *S*_*n*_-rotation can be represented by sequentially applying the symmetry operations above.

### 2.3 Point-Group Symmetry for the Light−Matter System

To maintain the symmetries for the coupled light-matter system, also the displacement field should be set up in a basis of perpendicularly polarized modes

$$\mathrm{D̂}=\sum_{\alpha }^{N_{\mathrm{cav}}}\sqrt{\frac{\lambda_{\alpha }^{2}\omega_{\alpha }}{2}}\left[\epsilon_{\alpha }\left({\mathrm{b̂}}_{\alpha }^{†}+{\mathrm{b̂}}_{\alpha }\right)+{\mathrm{\epsilon ̅}}_{\alpha }\left({\hat{\mathrm{b̅}}}_{\alpha }^{†}+{\hat{\mathrm{b̅}}}_{\alpha }\right))\right]$$

Note that to retain the symmetries of the cavity, the coupling strength along two perpendicular polarized modes must be equal λ_α,1_ = λ_α,2_ ≡ λ_α_.

Note, however, that the displacement operator ***D* ^** is not invariant under *C*_∞_ rotations. The rotations can be written as

$${Ĉ}_{\theta }^{†}\mathrm{D̂}{Ĉ}_{\theta }=\sum_{\alpha }\sqrt{\frac{\lambda_{\alpha }^{2}\omega_{\alpha }}{2}}\left[\epsilon_{\alpha }^{\prime }\left({\mathrm{b̂}}_{\alpha }^{†}+{\mathrm{b̂}}_{\alpha }\right)+{\mathrm{\epsilon ̅}}_{\alpha }^{\prime }\left({\hat{\mathrm{b̅}}}_{\alpha }^{†}+{\hat{\mathrm{b̅}}}_{\alpha }\right))\right]$$

with the new basis

$$\begin{array}{l} \epsilon_{\alpha }^{\prime }=\left(\cos\theta \epsilon_{\alpha }-\sin\theta {\mathrm{\epsilon ̅}}_{\alpha }\right)\\ {\mathrm{\epsilon ̅}}_{\alpha }^{\prime }=\left(\cos\theta {\mathrm{\epsilon ̅}}_{\alpha }+\sin\theta \epsilon_{\alpha }\right) \end{array}$$

i.e., the polarization vectors change. For example, for a rotation of θ = π, the displacement field is inverted: *Ĉ*_π_^†^ ***D* ^***Ĉ*_π_ = −***D* ^**.

However, the bilinear coupling ***D̂*** · ***d* ~**^ is invariant under a *Ĉ*_θ_ rotation

$${Ĉ}_{\theta }^{†}\mathrm{D̂}\cdot \mathrm{d̃̂}{Ĉ}_{\theta }=\mathrm{D̂}\cdot \mathrm{d̃̂}$$

This is ensured by the fact that the **ϵ**· ***d* ~**^ and **ϵ̅**· ***d* ~**^ components of the molecular dipole operator transform in the same manner as the displacement field ***D* ^**. In the example above, with θ = π, the **ϵ**· ***d* ~**^ and **ϵ̅**· ***d* ~**^ components are inverted as well.[Fn fn1] A similar behavior is found for the vertical *C*_2_ rotations and σ_*v*_ reflections. This holds for all symmetry operations of the group.

This shows that a rotation of **ϵ**_α_ and **ϵ̅**_α_ does not change any physical properties and hence, for equal coupling strengths, an arbitrary linear combination can be formed. For a Fabry-Pérot cavity since all wave vectors ***k***_α_ are aligned in a parallel manner, the displacement field operator can always be written in terms of two unified field polarizations **ϵ** ≡ **ϵ**_α_ and **ϵ̅** ≡ **ϵ̅**_α_ for all α, so that

5 $$\mathrm{D̂}=\sum_{\alpha }^{N_{\mathrm{cav}}}\sqrt{\frac{\lambda_{\alpha }^{2}\omega_{\alpha }}{2}}\left[\epsilon \left({\mathrm{b̂}}_{\alpha }^{†}+{\mathrm{b̂}}_{\alpha }\right)+\mathrm{\epsilon ̅}\left({\hat{\mathrm{b̅}}}_{\alpha }^{†}+{\hat{\mathrm{b̅}}}_{\alpha }\right))\right]$$

For the combined light-matter system the *D*_∞*h*_ symmetry of the cavity may be broken, due to the parametrically introduced nuclei in *V̂*_eN_, see eq 1. A *D*_∞*h*_ symmetry is therefore only obtained for atoms or linear molecules of *D*_∞*h*_ symmetry. For the polaritonic wave function |Ψ⟩, in general

$$\left\langle \Psi \right|{\mathrm{b̂}}_{\alpha }^{†}{\mathrm{b̂}}_{\alpha }\left|\Psi \right\rangle \neq \left\langle \Psi \right|{\hat{\mathrm{b̅}}}_{\alpha }^{†}{\hat{\mathrm{b̅}}}_{\alpha }\left|\Psi \right\rangle$$

i.e., the two displacement modes may exhibit differing levels of excitation. Exceptions are atoms and linear molecules oriented parallel along ***k***_α_. In all systems, however, the energy is invariant among rotations of the polarization vectors.[Fn fn2]

It should be noted that, in our description, the cavity is neither linearly nor circularly polarized. Moreover, complex-valued polarization vectors can be employed without loss of generality. The equivalence of these descriptions can be demonstrated via a unitary transformation, as detailed in Appendix A.

We also mention that assuming equal coupling strengths along the two perpendicular polarizations is an idealization and that in an experimental setup, the coupling strengths may differ. I.e., in realistic experimental setups, imperfections in the cavity mirrors can lead to unequal coupling strengths for different polarization directions, such that the emitter couples more strongly to one polarization than to the other. In this case, the symmetry of the coupled light–matter system is reduced, which can lift degeneracies and give rise to a more complex polaritonic energy landscape. Moreover, such deviations from the idealized model are inherently connected to cavity losses, which are typically not included in current many-body cavity QED approaches. In practice, photon leakage into the external continuum occurs due to finite mirror reflectivity, and the associated loss rates may depend on the polarization of the cavity modes.

### 2.4 Comparison to the Single-Polarization Approximation

The term single-polarization approximation refers to the scenario in which the light–matter interaction is described by only a single polarization of the electromagnetic field. This is equivalent to eliminating one polarization component by setting λ̅_α_ = 0. This should not be confused with the single-mode approximation which is equivalent to fixing *N*_cav_ = 1. Figure 1 illustrates this situation, where depending on the approximation in the Hamiltonian, the cavity symmetry changes

In the upper left corner, a general Fabry-Pérot cavity is depicted which is characterized by the principal rotation axis *C*_∞_ pointing in the direction of the two mirrors. The system has an overall *D*_∞*h*_ symmetry. When eliminating one field polarization, a linearly polarized cavity is obtained and the symmetry is reduced to *D*_2*h*_—see lower left panel in Figure 1. The cavity therefore only supports light of one polarization and the principal rotation axis is reduced here from *C*_∞_ to *C*_2_, noting that two orthogonal *C*_2_ rotations remain.

When applying the dipole approximation, it is assumed that the wavelength of the EM field is large over the dimension of the molecule and that the matter−system is not close to the boundaries. In Figure 1 this is realized by removing the mirrors so that the electric field has no boundaries. This can be understood as an rotationally symmetric electric field pointing in an arbitrary direction in the *xy*-plane—see upper right corner of Figure 1. When additionally assuming that one field polarization is not coupled to the molecule, one of the polarization vectors is removed by which the electric field is given a predefined polarization. This can be seen in Figure 1 in the lower-right corner, where a homogeneous electric field is oriented along the ϵ_*x*_-vector. Note that in consequence, an additional *C*_∞_ rotation axis is formed in the direction of the polarization vector. This means that the symmetry of the system in the dipole approximation together with the single-polarization treatment is higher (*D*_∞*h*_) than in the setup for a linearly polarized cavity (*D*_2*h*_), i.e., the lower left part of Figure 1. Also, when comparing the results of an unpolarized Fabry-Pérot cavity to a linearly polarized cavity in the dipole approximation, the point-group might be the same (*D*_∞*h*_), but the principal axis of the system is shifted by 90°, which leads to a different symmetry.

We mention that the linearly polarized cavity (λ̅_α_ = 0) and the unpolarized cavity (λ_α_ = λ̅_α_) are both idealized Fabry-Pérot cavities. The experimental coupling strength would depend on the specific system parameters, e.g., the quality of the cavity, the position of the emitter in the cavity, thermodynamic fluctuations within the mirrors, etc., which corresponds to a situation where λ_α_ ≠ λ̅_α_. Nonetheless, most current developments in many-body methods focus on the idealized case of a single polarization, while the case of an unpolarized cavity has so far not been explored. Future work should also consider the more general case of λ_α_ ≠ λ̅_α_, which can be understood as the transition between the cases of a linearly polarized cavity and an unpolarized cavity, respectively.

### 2.5 Unpolarized Polaritonic CC Theory

The polaritonic CC wave function ansatz is^17^

$$\left|\Psi_{\mathrm{CC}}\right\rangle =e^{\mathrm{Q̂}}\left|0,0\right\rangle$$

with the cluster operator *Q̂*. The state |0,0⟩ is the Fermi-vacuum and represents the HF determinant with the photonic vacuum. Both numbers in |0,0⟩ can be understood as occupation number vectors (ONVs), where the first number designates the electronic ONV and the second the photonic ONV. For the electronic space, a “0” designates the case where all electrons are located in occupied orbitals, the state $\left|\begin{array}{l} a\\ i \end{array},0\right\rangle$ is a determinant where an electron is excited from the orbital *i* into the virtual orbital *a* and similarly for double excitations $\left|\begin{array}{l} \mathrm{ab}\\ \mathrm{ij} \end{array},0\right\rangle$ . In the photonic space a “0” represents the absence of photons in all modes, i.e., the physical vacuum. The state |0, 1_*n*_⟩ designates a state where one photon is in the *n*’th mode, and the state |0, 1_*n*_1_*n̅*_⟩ are two photons, one in mode *n* and the other in the perpendicular polarized mode *n̅*. This scheme can easily be generalized to include higher photonic excitations and other modes.

In this work the CCSD-12-SD truncation scheme is employed, with the cluster operator truncated as^17,47^

$$\mathrm{Q̂}={\mathrm{T̂}}_{1}+{\mathrm{T̂}}_{2}+{Ŝ}_{1}^{1}+{Ŝ}_{2}^{1}+{\mathrm{\Gamma ̂}}_{1}+{\mathrm{\Gamma ̂}}_{2}$$

Here, *T̂*_1_ and *T̂*_2_ are the standard electronic single and double excitation operators, *Ŝ*_1_^1^ and *Ŝ*_2_^1^ are the mixed operators that include one-photon creation, and Γ̂_1_ and Γ̂_2_ are the bare one- and two-photon creation operators. The Γ̂_1_ operator is set up as

$${\mathrm{\Gamma ̂}}_{1}=\sum_{\alpha }\left(\gamma^{\alpha }{\mathrm{b̂}}_{\alpha }^{†}+\gamma^{\mathrm{\alpha ̅}}{\hat{\mathrm{b̅}}}_{\alpha }^{†}\right)$$

with the two sets of amplitudes corresponding to perpendicular field polarizations (γ^α^ and γ^α̅^). In the same manner, the Γ̂^2^ operator can be set up as

$${\mathrm{\Gamma ̂}}_{2}=\sum_{\alpha <\beta }\left(\gamma^{\alpha \beta }{\mathrm{b̂}}_{\alpha }^{†}{\mathrm{b̂}}_{\beta }^{†}+\gamma^{\mathrm{\alpha ̅}\mathrm{\beta ̅}}{\hat{\mathrm{b̅}}}_{\alpha }^{†}{\hat{\mathrm{b̅}}}_{\beta }^{†}+\gamma^{\alpha \mathrm{\beta ̅}}{\mathrm{b̂}}_{\alpha }^{†}{\hat{\mathrm{b̅}}}_{\beta }^{†}+\gamma^{\mathrm{\alpha ̅}\beta }{\hat{\mathrm{b̅}}}_{\alpha }^{†}{\mathrm{b̂}}_{\beta }^{†}\right)$$

Regarding the Γ̂_2_ operator, when introducing unrestricted sums, the diagonal elements of γ^*αα*^ and γ̿^*αα*^ would falsely be scaled by a factor of $\frac{1}{2}$ . This must be taken into account by using

$$\gamma^{\alpha \beta }\to \left(1+\delta_{\alpha \beta }\right)\gamma^{\alpha \beta }$$

and similarly for γ^α̅β̅^, but not for γ^αβ̅^ as the latter corresponds to an off-diagonal block and in general is not symmetric. When further exploiting the symmetry γ^αβ̅^ = γ^β̅α^ and the commutator [α^†^, β̅^†^] = 0, the Γ̂_2_ operator becomes

$${\mathrm{\Gamma ̂}}_{2}=\frac{1}{2}\sum_{\alpha \beta }\left(\gamma^{\alpha \beta }{\mathrm{b̂}}_{\alpha }^{†}{\mathrm{b̂}}_{\beta }^{†}+\gamma^{\mathrm{\alpha ̅}\mathrm{\beta ̅}}{\hat{\mathrm{b̅}}}_{\alpha }^{†}{\hat{\mathrm{b̅}}}_{\beta }^{†}+2\gamma^{\mathrm{\alpha ̅}\beta }{\mathrm{b̂}}_{\alpha }^{†}{\hat{\mathrm{b̅}}}_{\beta }^{†}\right)$$

The exploitation of polarization symmetry works therefore completely analogously to the exploitation of spin symmetry. E.g., in a spin-unrestricted treatment, the *T̂*_2_ operator is parametrized as

$${\mathrm{T̂}}_{2}=\frac{1}{4}\sum_{\mathrm{abij}}t_{\mathrm{abij}}{â}_{a}^{†}{â}_{i}{â}_{b}^{†}{â}_{j}+\frac{1}{4}\sum_{\mathrm{aibj}}t_{\mathrm{a̅}\mathrm{b̅}\mathrm{i̅}\mathrm{j̅}}{\hat{\mathrm{a̅}}}_{a}^{†}{\hat{\mathrm{a̅}}}_{i}{\hat{\mathrm{a̅}}}_{b}^{†}{\hat{\mathrm{a̅}}}_{j}+\sum_{\mathrm{aibj}}t_{a\mathrm{b̅}i\mathrm{j̅}}{â}_{a}^{†}{â}_{i}{\hat{\mathrm{a̅}}}_{b}^{†}{\hat{\mathrm{a̅}}}_{j}$$

where for the amplitudes we used that *t*_*aib̅ j̅*_ = −*t*_*ai̅b̅j*_ = −*t*_*a̅ibj̅*_ = *t*_*a̅i̅bj*_. This partitioning for the photonic and electronic indices can also be applied for the *Ŝ*_1_^1^ and *Ŝ*_2_^1^ operators

$${Ŝ}_{1}^{1}=\sum_{\mathrm{ai}\alpha }\left(s_{\mathrm{ai}}^{\alpha }{\mathrm{b̂}}_{\alpha }^{†}+s_{\mathrm{ai}}^{\mathrm{\alpha ̅}}{\hat{\mathrm{b̅}}}_{\alpha }^{†}\right){â}_{a}^{†}{â}_{i}+\sum_{\mathrm{ai}\alpha }\left(s_{\mathrm{a̅}\mathrm{i̅}}^{\alpha }{\mathrm{b̂}}_{\alpha }^{†}+s_{\mathrm{a̅}\mathrm{i̅}}^{\mathrm{\alpha ̅}}{\hat{\mathrm{b̅}}}_{\alpha }^{†}\right){\hat{\mathrm{a̅}}}_{a}^{†}{\hat{\mathrm{a̅}}}_{i}$$

$${Ŝ}_{2}^{1}=\frac{1}{4}\sum_{\mathrm{abij}\alpha }\left(s_{\mathrm{aibj}}^{\alpha }{\mathrm{b̂}}_{\alpha }^{†}+s_{\mathrm{aibj}}^{\mathrm{\alpha ̅}}{\hat{\mathrm{b̅}}}_{\alpha }^{†}\right){â}_{a}^{†}{â}_{i}{â}_{b}^{†}{â}_{j}+\frac{1}{4}\sum_{\mathrm{abij}\alpha }\left(s_{\mathrm{a̅}\mathrm{i̅}\mathrm{b̅}\mathrm{j̅}}^{\alpha }{\mathrm{b̂}}_{\alpha }^{†}+s_{\mathrm{a̅}\mathrm{i̅}\mathrm{b̅}\mathrm{j̅}}^{\mathrm{\alpha ̅}}{\hat{\mathrm{b̅}}}_{\alpha }^{†}\right){\hat{\mathrm{a̅}}}_{a}^{†}{\hat{\mathrm{a̅}}}_{i}{\hat{\mathrm{a̅}}}_{b}^{†}{\hat{\mathrm{a̅}}}_{j}+\sum_{\mathrm{abij}\alpha }\left(s_{\mathrm{ai}\mathrm{b̅}\mathrm{j̅}}^{\alpha }{\mathrm{b̂}}_{\alpha }^{†}+s_{\mathrm{ai}\mathrm{b̅}\mathrm{j̅}}^{\mathrm{\alpha ̅}}{\hat{\mathrm{b̅}}}_{\alpha }^{†}\right){â}_{a}^{†}{â}_{i}{\hat{\mathrm{a̅}}}_{b}^{†}{\hat{\mathrm{a̅}}}_{j}$$

If the molecule is symmetric for rotations along the orientation of the cavity, the symmetries γ^α^ = γ ^α̅^, *s*_*ai*_^α^ = *s*_ai_^α̅^ and *s*_*abij*_^α^ = *s*_*abij*_^α̅^ can be employed to give an even more compact notation. E.g., the Γ̂_1_ operator would be set up as

$${\mathrm{\Gamma ̂}}_{1}=\sum_{\alpha }\gamma^{\alpha }\left({\mathrm{b̂}}_{\alpha }^{†}+{\hat{\mathrm{b̅}}}_{\alpha }^{†}\right)$$

and similarly for *Ŝ*_1_^1^ and *Ŝ*_2_^1^. The CC amplitudes are solved in a nonlinear set of projected equations

$$\left\langle \mu ,\nu \right|\hat{\mathrm{H̃}}\left|0,0\right\rangle =0$$

with μ designating an electronically excited determinant and ν an excitation of the EM field. The similarity-transformed Hamiltonian *H̃̂* is given as

$$\hat{\mathrm{H̃}}=e^{-\mathrm{Q̂}}Ĥe^{\mathrm{Q̂}}$$

A detailed description of how the CC amplitude equations are solved can be found in ref 48.

To calculate properties and densities at the CC level of theory, the left-hand side solution of the similarity-transformed Hamiltonian matrix is required. The left-hand side wave function is given as^48,49^

$$\left\langle \Psi_{\mathrm{CC}}^{L}\right|=\left\langle 0,0\right|\left(1+\mathrm{\Lambda ̂}\right)e^{-\mathrm{Q̂}}$$

with Λ̂ being a de-excitation operator truncated in the same scheme as *Q̂*. The coefficients in Λ̂ can be determined via a set of projected equations

$$\left\langle 0,0\right|\left(1+\mathrm{\Lambda ̂}\right)\hat{\mathrm{H̃}}\left|\mu ,\nu \right\rangle =0$$

With a converged set of Λ-amplitudes, polaritonic expectation values can be obtained in the familiar manner via

$$\left\langle O\right\rangle =\left\langle \Psi_{\mathrm{CC}}^{L}\right|Ô\left|\Psi_{\mathrm{CC}}\right\rangle$$

This also allows for the calculation of the electron and photon density matrices. E.g., the one-electron density matrix is obtained as

$$\gamma_{\mathrm{pq}}=\left\langle \Psi_{\mathrm{CC}}^{L}\right|{â}_{p}^{†}{â}_{q}\left|\Psi_{\mathrm{CC}}\right\rangle$$

A detailed description of how the Λ-equations are solved and how the density matrices are calculated can be found in ref 48. The one-electron density matrix is used with the molecular orbitals ϕ_*p*_(***r***) to assemble the one-electron density

$$\rho \left(r\right)=\sum_{\mathrm{pq}}\gamma_{\mathrm{pq}}\phi_{p}^{*}\left(r\right)\phi_{q}\left(r\right)$$

The one-electron density gives the probability of finding an electron in the finite volume d ***r*** at a position ***r***. When integrating the one-electron density over all Cartesian coordinates, the total number of electrons is obtained, i.e., ∫_–∞_^∞^ ρ­(***r***)­d ***r*** = *N*_el_.

Excited states can be obtained from the converged set of CC-amplitudes via the equation-of-motion-CC (EOM-CC) parametrization^50^

$$\hat{\mathrm{H̃}}\mathrm{R̂}\left|0,0\right\rangle =E_{\mathrm{exc}}\mathrm{R̂}\left|0,0\right\rangle$$

where the excitation operator *R̂* is truncated in the same scheme as the cluster operator *Q̂*. Therefore

$$\mathrm{R̂}={{\mathrm{R̂}}_{1}}^{0}+{\mathrm{R̂}}_{2}^{0}+{\mathrm{R̂}}_{1}^{1}+{\mathrm{R̂}}_{2}^{1}+{\mathrm{R̂}}_{0}^{1}+{\mathrm{R̂}}_{0}^{2}$$ 6

with *R̂*_μ_^ν^ consisting of electronic excitation and photonic creation operators. For example, the mixed one-electron one-photon operator *R̂*_1_^1^ is given as

$${\mathrm{R̂}}_{1}^{1}=\sum_{\mathrm{ai}\alpha }r_{\mathrm{ai}}^{\alpha }{â}_{a}^{†}{â}_{i}{\mathrm{b̂}}_{\alpha }^{†}$$

Like in electronic CC theory, the excitation operator *R̂* commutes with the cluster operator *Q̂*. The coefficients in *R̂* can be obtained by a Davidson-like algorithm.^51^ But, while the cluster operator *Q̂* and the de-excitation operator Λ̂ transform in a totally symmetric manner, the excitation operator *R̂* transforms according to the irreducible representation of the excitation. A detailed description of how the EOM-CC equations are solved for the coefficients in *R̂* can be found in ref 48.

For a validation of the method, the reader is referred to refs 17 and 47, where the polaritonic QED-CCSD-1-SD and the QED-CCSD-12-SD method is benchmarked against near-exact QED-FCI-5 calculations, showing that QED-CCSD-1-SD calculations lead to quantitative results for intermolecular interactions and that QED-CCSD-12-SD calculations also lead well converged results for intramolecular interactions.

### 2.6 Exploiting Point-Group Symmetry in Cavity-QED Calculations

Exploiting point-group symmetry in the context of direct-product decomposition in quantum-chemical calculations leads to significant speedups in computational time, reduces the memory requirements^38^ and enables the targeted calculation of states of different irreducible representations. For the treatment of electronic operators in terms of the direct-product decomposition, the reader is referred to ref 38. The following discussion will focus on the bilinear coupling and the photonic Hamiltonian.

As discussed before, the Hamiltonian is totally symmetric for symmetry operations within the point group. Consequently, all operators in the Hamiltonian are characterized by the totally symmetric irreducible representation Γ_1_.^45^ For the cavity Hamiltonian *Ĥ*_cav_ this property is rather obvious, as by construction it is build with the number operator *b̂*_α_^†^ *b̂*_α_ and the direct product of an irreducible representation with itself always contains the totally symmetric representation

$$\Gamma_{1}\in \Gamma_{\alpha }⊗\Gamma_{\alpha }$$

The bilinear coupling operator in second quantization for two perpendicular polarized modes can be written in the form

$${Ĥ}_{\mathrm{bil}}=\sum_{\alpha }^{N_{\mathrm{cav}}}\sum_{\mathrm{pq}}{\mathrm{d̃}}_{\mathrm{pq}}^{\alpha }\left({\mathrm{b̂}}_{\alpha }+{\mathrm{b̂}}_{\alpha }^{†}\right){â}_{p}^{†}{â}_{q}+\sum_{\alpha }^{N_{\mathrm{cav}}}\sum_{\mathrm{pq}}{\mathrm{d̃}}_{\mathrm{pq}}^{\mathrm{\alpha ̅}}\left({\hat{\mathrm{b̅}}}_{\alpha }+{\hat{\mathrm{b̅}}}_{\alpha }^{†}\right){â}_{p}^{†}{â}_{q}$$

with *â*_*p*_^†^ and *â*_*p*_ being the electronic creation and annihilation operators for an electron in orbital *p*. A single term of the bilinear coupling operator is totally symmetric if the direct product of the irreducible representation of the elementary operators contains the Γ_1_ representation, i.e.,

$$\Gamma_{1}\in \Gamma_{p}⊗\Gamma_{q}⊗\Gamma_{\alpha }$$

Depending on the molecule and its orientation, the bilinear coupling can be grouped in the following way: If the molecule has a dipole moment oriented along one polarization vector, the photonic index is totally symmetric (Γ_α_ = Γ_1_) and the bilinear coupling integrals *d̃*_*pq*_^α^ become block diagonal in the electronic indices (Γ_*p*_ = Γ_*q*_). On the other hand, if the molecule has no dipole moment, or a dipole moment not aligned with the polarization vector, the irreducible representation of the photonic index is not totally symmetric (Γ_α_ ≠ Γ_1_) and hence the diagonal blocks for the electronic indices vanish (Γ_*p*_ ≠ Γ_*q*_). Thus, the implementation needs to be flexible enough to handle both block-diagonal matrices and matrices with nonzero blocks on the off-diagonals—see Figure 2.

**Figure 2:**
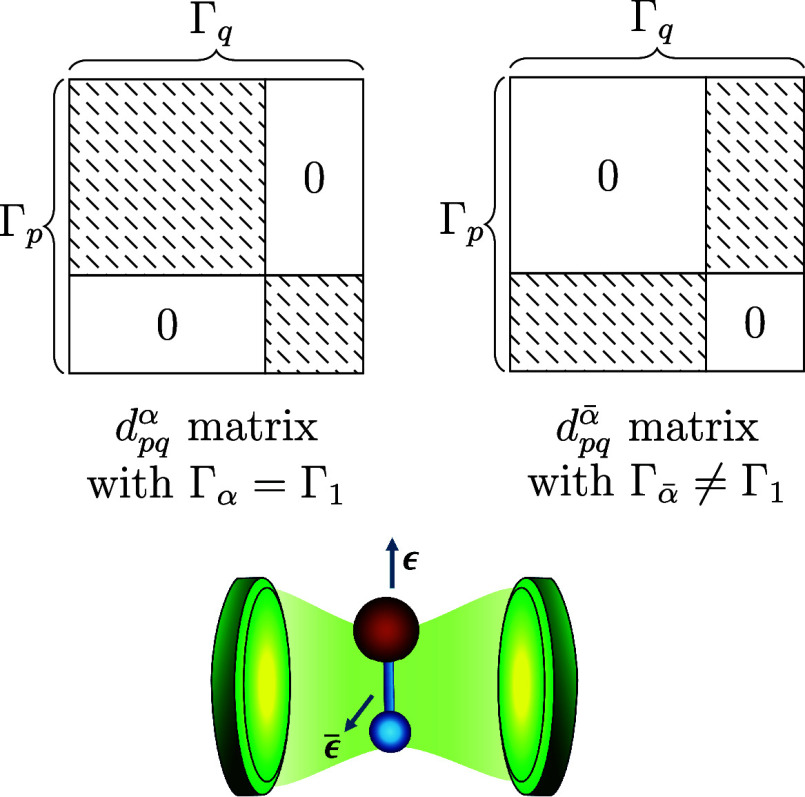
Exemplary matrix representation of the bilinear coupling *d̃*_*pq*_^α^ and *d̃*_*pq*_^α̅^. The polarization **ϵ** is here equal to the totally symmetric irreducible representation Γ_α_ = Γ_1_ and the electronic indices become block diagonal. The second polarization **ϵ̅** is not totally symmetric and the occupation for the electronic indices is on the off-diagonal blocks.

In principle, the orientation of the polarization vectors is arbitrary as long as they are perpendicular to **k** and among each other. Here, we chose one polarization vector, e.g., **ϵ**, to be aligned parallel to the permanent molecular dipole moment and the second polarization vector **ϵ̅** perpendicular to it. The bilinear coupling operator then reads

$${Ĥ}_{\mathrm{bil}}=\sum_{\alpha \in \Gamma_{1}}\sum_{\Gamma_{\mathrm{el}}}\sum_{\begin{array}{l} p\in \Gamma_{\mathrm{el}}\\ q\in \Gamma_{\mathrm{el}} \end{array}}{\mathrm{d̃}}_{\mathrm{pq}}^{\alpha }\left({\mathrm{b̂}}_{\alpha }+{\mathrm{b̂}}_{\alpha }^{†}\right){â}_{p}^{†}{â}_{q}+\sum_{\begin{array}{l} \Gamma_{1}\in \\ \Gamma_{\mathrm{ph}}⊗\Gamma_{p}⊗\Gamma_{q} \end{array}}\sum_{\begin{array}{l} p\in \Gamma_{p}\\ q\in \Gamma_{q}\\ \mathrm{\alpha ̅}\in \Gamma_{\mathrm{ph}} \end{array}}{\mathrm{d̃}}_{\mathrm{pq}}^{\mathrm{\alpha ̅}}\left({\hat{\mathrm{b̅}}}_{\alpha }+{\hat{\mathrm{b̅}}}_{\alpha }^{†}\right){â}_{p}^{†}{â}_{q}$$

where the second term only runs over irreducible representations where Γ_1_ ∈ Γ_ph_ ⊗ Γ_*p*_ ⊗ Γ_*q*_ is fulfilled and Γ_ph_ ≠ Γ_1_. An exemplary representation of the bilinear coupling operator in matrix representation can be found in Figure 2.

Handling of symmetry based on the direct-product decomposition in the context of CC theory is well-known for Abelian groups^38,52^ and has been exploited in many quantum-chemical program packages. It ensures that the number of floating-point operations can be reduced by a factor of *h* ^2^, with the order of the group, *h*.^52^ For use within QED-CC theory, the photonic indices must be taken into account.

As the cluster operator *Q̂* must be totally symmetric, it is obvious that the γ^α^ amplitude vanishes when *b̂*_α_^†^ is not totally symmetric itself. In other words, the molecule must have a dipole moment along **ϵ** in order to have nonvanishing contributions in γ^α^. For the same reason in the mixed amplitudes *s*_*ia*_^α^ and *s*_*ijab*_^α^ the electronic indices do not have to contain the totally symmetric irreducible representation as long as the full operator is totally symmetric. An exemplary contraction from the QED-CC amplitude equations is
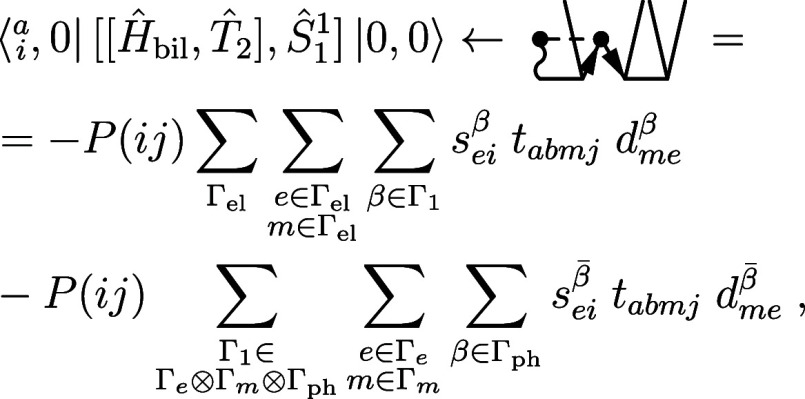
where the polarization vector **ϵ** is parallel to the molecular dipole moment and the second polarization vector **ϵ̅** perpendicular to it. The sum over Γ_el_ is over all irreducible representations in the group and further Γ_ph_ ≠ Γ_1_. The amplitudes *s*_*ei*_^α^ and *s*_*ei*_^α̅^ can be grouped into blocks similar to the bilinear coupling integrals *d*_*pq*_^α^ and *d*_*pq*_^α̅^, see Figure 2.

Exploiting the block-structure of tensors in the above manner ensures that only the potentially nonzero elements are computed which leads to time and memory savings. Further, it allows to make use of the usual integral-packing strategies for the permutations of indices of the different irreducible representations. For example, the mixed amplitudes *s*_*aibj*_^α^ can be packed exploiting the block structure

$$\begin{array}{l} s_{\Gamma_{A}\Gamma_{B}\Gamma_{A}\Gamma_{C}}^{\Gamma_{\mathrm{ph}}}=-s_{\Gamma_{A}\Gamma_{B}\Gamma_{C}\Gamma_{A}}^{\Gamma_{\mathrm{ph}}}=s_{\Gamma_{B}\Gamma_{A}\Gamma_{C}\Gamma_{A}}^{\Gamma_{\mathrm{ph}}}=-s_{\Gamma_{B}\Gamma_{A}\Gamma_{A}\Gamma_{C}}^{\Gamma_{\mathrm{ph}}} \end{array}$$

Similar to regular CC theory, the most significant amount of memory can hence be saved by packing the two-electron integrals *g̃*_*pqrs*_ and additionally the four- and five-index amplitudes *t*_*ijab*_ and *s*_*ijab*_^α^. Overall, the memory requirements are then similar as compared to standard CC implementations.

### 2.7 Implementation

The evaluation of integrals and the self-consistent-field iterations are carried out in the CFOUR program package.^53,54^ The QED-CC and EOM-QED-CC steps are carried out in the Qcumbre program prackage.^55,56^ The algorithm exploits the block structure of matrices in all stages, i.e., the integral calculation, the QED self-consistent-field calculation, the integral transformation, and in the CC and EOM-CC algorithms. The implemented algorithm was verified for single-polarization cavities by comparing results obtained using the *e* ^T^ program package^57^ for different systems, frequencies, and molecular orientations with respect to the polarization vector. The algorithm was designed such that the single polarization appears as a special case of a cavity including an arbitrary number of EM modes.

## 3 Results and Discussion

All calculations presented in this work were performed using a developer’s version of the CFOUR^53,54^ and Qcumbre^55,56^ program packages for the HF and CC steps, respectively. All calculations employ point-group symmetry in all stages and were performed using a cc-pVTZ basis set^58^ unless stated otherwise. The convergence criterion for all iterative steps was 10^–8^ *E*_h_ or smaller. The molecular geometries have been optimized in absence of the cavity using the ORCA^59^ program package on the DFT-B3LYP/def2-SVP level of theory. The coupling strength was fixed to λ = 0.05 au if not stated otherwise. For all calculations the light−matter interaction is described with a single cavity frequency ω and interactions with other frequencies are neglected.

The algorithm exploits the block structure of matrices in all stages, i.e., the integral calculation, the QED self-consistent-field calculation, the integral transformation, and in the CC and EOM-CC algorithms.

The presented one-electron densities were calculated on a grid with at least 10^2^ grid points in each dimension and subsequently integrated over one coordinate. E.g., the integration of the *z* coordinate yields

$$\rho \left(x,y\right)=\int_{-\infty }^{\infty }dz\rho \left(x,y,z\right)$$

which can be visualized in a contour matrix.

To assess how the electrons are affected by the cavity, the density difference between the molecule in the cavity and the free molecule is calculated as Δρ­(***r***) = ρ_cav_(***r***) – ρ_ref_(***r***). An indicator to quantify the influence of the cavity on the electrons is obtained by integrating the absolute density difference

$$\Delta \rho =\int_{-\infty }^{\infty }d^{3}r\left|\Delta \rho \left(r\right)\right|$$

When dividing by two, this quantity can be understood as the fraction of an electron that is shifted in space and is given in the upper left corner of all density plots in the following. We stress that Δ*ρ* should not be interpreted as a rigorous observable, but rather as a convenient diagnostic quantity.

The effect of mass-renormalization is not included in the presented calculations, and the following results are to be understood as a proof of concept for the implementation of the algorithm and the exploitation of symmetry in the context of linearly polarized and unpolarized cavities. Currently, mass-renormalization effects are commonly ignored in *ab initio* QED calculations,^44,60^ such as QED-CC or QEDFT, also for very high coupling strengths (λ > 0.01 au).^17,22,61–64^

### 3.1 Benzene

Aromatic species are particularly interesting for polaritonic chemistry as they have isolated energetic low-lying excited states which can be easily addressed by tuning the cavity frequency.^65^

We compare results for benzene in a linearly polarized (see also ref 64) and unpolarized cavity, respectively. The benzene molecule has *D*_6*h*_ symmetry, which is reduced in the presence of the cavity depending on the orientation of the polarization vectors with respect to the molecule. Only in the cases where the polarization vector **ϵ** or wave vector ***k*** is aligned perpendicularly to the molecular plane for the linear polarization and unpolarized cavity, respectively, the *D*_6*h*_ symmetry is maintained. For other orientations, the symmetry is reduced, e.g., if the wave vector is aligned within the molecular plane, the symmetry is reduced to *D*_2*h*_. As most program packages, our algorithm is designed to only utilize real Abelian groups up to *D*_2*h*_, as higher symmetries require a more general algorithm.

Figure 3 shows how the energy of the benzene molecule changes with respect to the cavity orientation for a linearly polarized cavity (left) and an unpolarized cavity (right), respectively.

**Figure 3:**
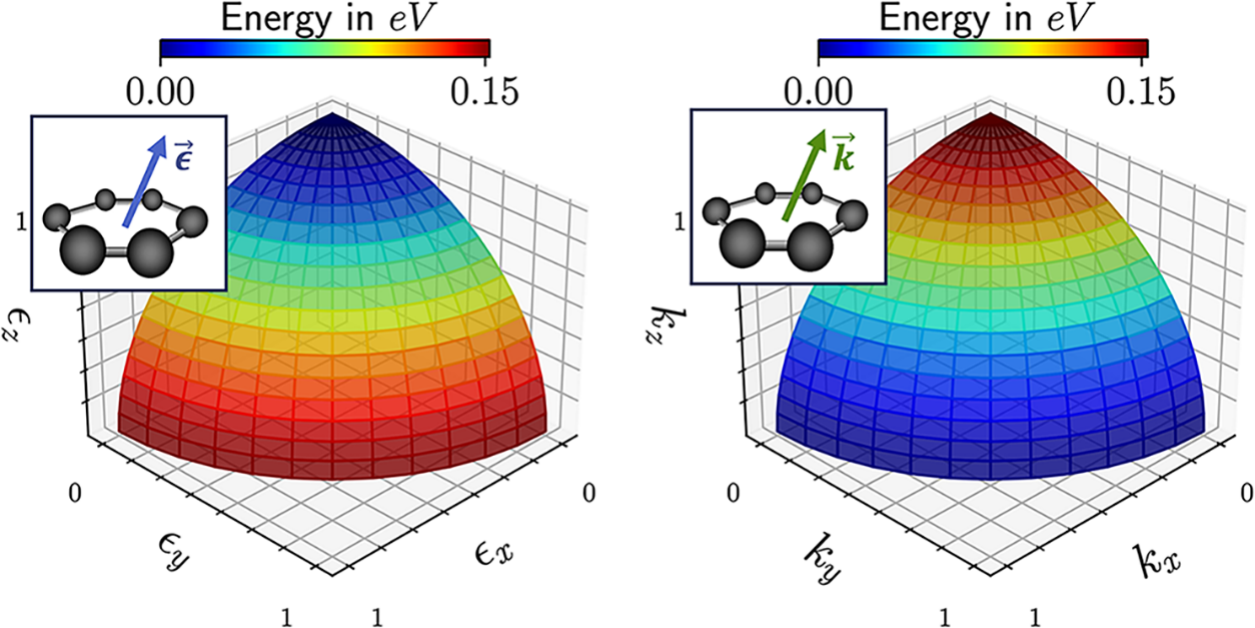
QED-CCSD-12-SD ground-state energy for the benzene monomer with respect to orientation of the polarization vector (left) and wave vector (right). Calculated using a cc-pVDZ basis, a cavity frequency of ω = 0.2 m*E*_h_, and a coupling strength of λ = 0.05 au.

The cavity introduces an anisotropy, and the preferred orientation of the molecule in a linearly polarized cavity is obtained when the polarization vector is aligned perpendicular to the molecular plane (ϵ_*z*_). Thus, overall the molecule exhibits two degenerate energy minima, for the polarization vector aligned along ϵ_*z*_ and −ϵ_*z*_. In addition, an energy barrier of about 0.15 eV between the in plane and out-of-plane orientations is found. Rotating the polarization vector within the molecular plane causes only minor changes in the energy. I.e., these changes are one order of magnitude lower compared to changes in the angle with respect to the molecular plane.

For the molecule in an unpolarized cavity, the situation is different: The molecule is stabilized if the wave vector ***k*** is oriented within the molecular plane. For a wave vector oriented perpendicular to the molecular plane, i.e., with ***k*** = (0, 0, ± |*k*|), the potential surface features a maximum.

Hence, the potential energy surface is fundamentally modified depending on whether the molecule is placed in a linearly polarized or an unpolarized cavity, respectively. In a linearly polarized cavity, two distinct molecular orientations correspond to energy minima, and rotational motion is restricted by an associated potential barrier. In contrast, in an unpolarized cavity, stabilization occurs for a range of orientations in which the wave vector ***k*** lies within the molecular plane (forming an energy valley), while two distinct orientations with ***k*** orthogonal to the molecular plane correspond to energy maxima.

The calculated potential barrier is of about Δ*E* = 0.152 eV for the linearly polarized cavity and of Δ*E* = 0.153 eV for the unpolarized case in a cc-pVDZ basis. For a cc-pVTZ basis, the barrier decreases significantly to Δ*E* = 0.129 eV in the linearly polarized and Δ*E* = 0.131 eV in the unpolarized case. Further increasing the basis to cc-pVQZ shows that the barriers heights have essentially converged: Δ*E* = 0.127 eV for the linearly polarized and Δ*E* = 0.128 eV for the unpolarized case This behavior shows that the basis set has to be sufficiently large in order to correctly describe the self-energy contribution.

Considering an ensemble of molecules, the rotational barrier will lead to a preferred orientation of the molecules within the cavity. Whether the rotational barrier is observable in molecular spectra depends on the thermodynamic conditions of the system. In particular, at room temperature (*k*_B_ *T* ≈ 0.026 eV), the present barrier heights correspond to only a few *k*_B_ *T*, such that thermally activated rotational motion is expected to partially average out the anisotropy.

For comparison, the umbrella inversion in ammonia has a barrier of Δ*E* = 0.25 eV,^66^ whereas for phosphine the barrier is about Δ*E* = 1.70 eV, effectively suppressing inversion under ambient conditions.^67^ In the case of ammonia, the inversion is observable at room temperature via tunneling-induced splittings in microwave and infrared spectra, whereas in phosphine the much larger barrier suppresses tunneling and renders the inversion effectively unobservable under ambient conditions.

In contrast, the smaller rotational barriers found here suggest that any cavity-induced effects on rotational motion will likely be subject to significant thermal averaging, and their experimental observability will depend on temperature, rotational constants, and the achievable spectral resolution. Hence, for the cavity, due to the introduced anisotropy, a fine-structure splitting in the rovibrational spectrum should thus potentially become observable, particularly under low-temperature conditions where rotational averaging is reduced. The effect of the rotational barrier of molecules in cavities has also been investigated in ref 47 for an ensemble of hydrogen molecules, with the monomer exhibiting a rotation barrier of about Δ*E* = 0.018 eV and a coupling strength of λ = 0.1 au. For an ensemble of up to 144 molecules at a temperature of *T* = 70 K, a significant change in the orientation distribution of the molecules within the cavity was predicted. However, the extent of these effects depends heavily on the coupling parameter λ which corresponds to the coupling strength in the experimental setup, see also Figure S2 in the SI, where the barrier is calculated for various coupling strengths. Whether these effects are observable at room temperature has to be investigated in future work, as thermal fluctuations are expected to compete with the relatively small barrier heights and may lead to substantial orientational averaging. Therefore, direct experimental signatures may be reduced under ambient conditions and could require either stronger coupling strengths or lower temperatures to become clearly detectable. Nevertheless, the cavity-induced anisotropy provides a mechanism for modifying rotational energy landscapes, which may influence molecular dynamics even if only in an averaged sense.

We also mention that mass-renormalization effects are not included in the present calculation which can change the barrier height further. However, it is not expected that including mass-renormalization effects will change the qualitatively different shapes of the potential energy surfaces, as they are caused by a fundamentally different anisotropy in the cavity modes. Nevertheless, the influence of mass-renormalization effects is expected to change the quantitative values of the barrier height.

A closely related phenomenon has been reported for molecules subjected to finite magnetic fields. For example, refs 68 and 69 investigate how an external field modifies the rotation barrier and alters the resulting rotational dynamics. The observed anisotropy in the energy barrier will now be analyzed further using the one-electron densities.

The reduction in symmetry to *D*_2*h*_ is visible in the one-electron density differences in Figure 4 bottom for the more stable orientation in which the ***k*** vector lies in the molecular plane. Note that the left and right plots show different perspectives for the same orientation of the molecule within the cavity. In the plots, a buildup of electron density with respect to the cavity-free case is indicated in red while a decrease is shown in blue. In the cavity, the density becomes more localized in the C–C bonds and on the hydrogen atoms, see also ref 21, where density differences have been reported at the QEDFT level of theory. We note that, as expected, the density is mostly shifted along the polarization vectors.

**Figure 4:**
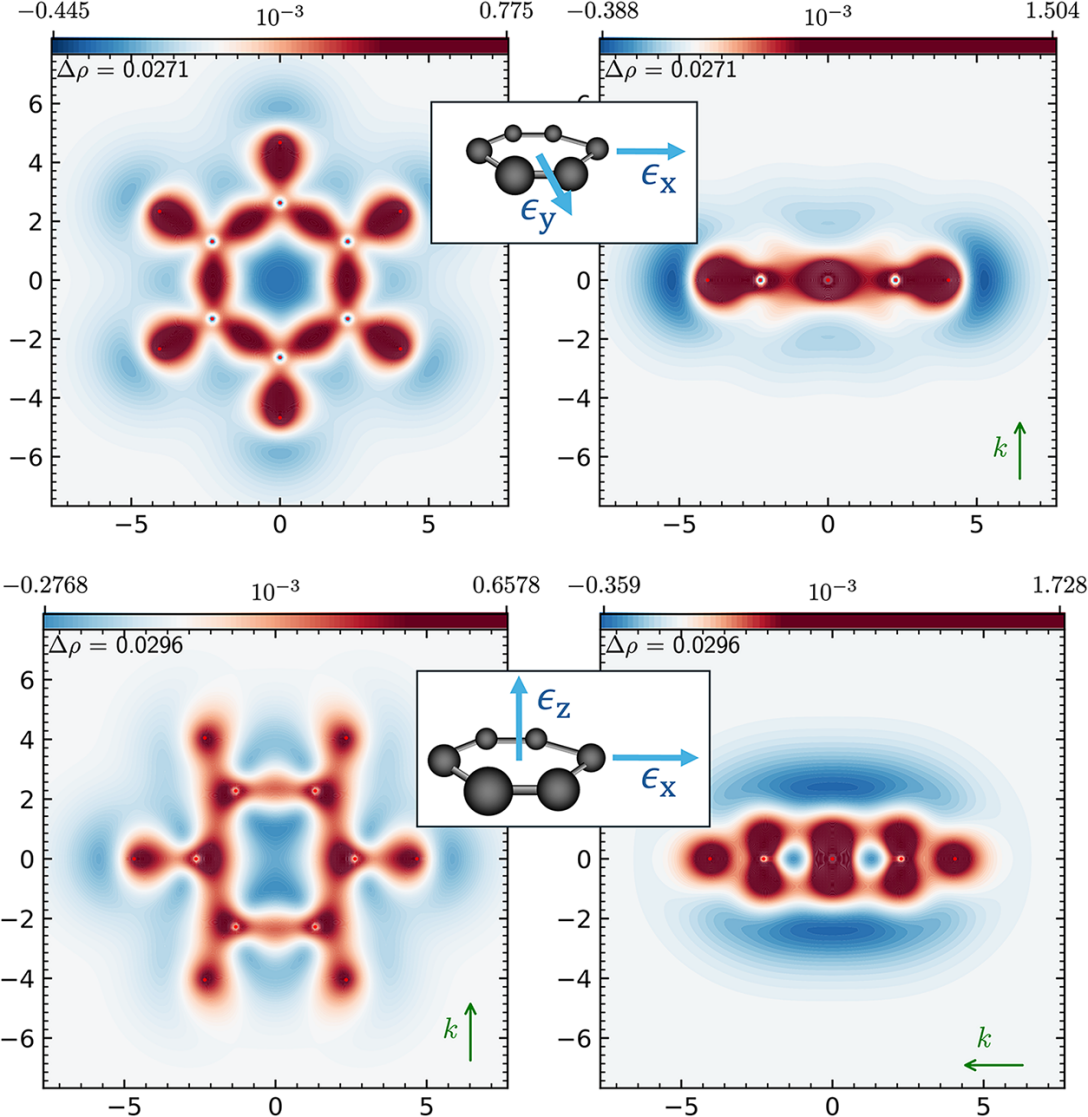
Correlated one-electron density difference for a benzene molecule in an unpolarized cavity. Left and right plots are different perspectives on the same density difference. The lower plots show the ***k*** vector aligned in the molecular plane and the upper plots show the ***k***-vector normal to the molecular plane. All QED-CCSD-12-SD calculations were performed using a cc-pVTZ basis, with a cavity frequency of ω_cav_ = 0.2 *E*_h_ and a coupling strength of λ = 0.2 au.

In the case where the ***k***-vector is aligned in the molecular plane, one polarization vector is oriented normal to the molecular plane and the density is partly shifted into the π-system (Figure 4 bottom right), while for the case where both polarization vectors are localized in the molecular plane, the density is shifted into the σ-system (Figure 4 top). For the ground state density, the difference Δ*ρ* is very small. For benzene, it ranges between 10^–2^ and 10^–1^, showing that the influence of an optical cavity on the ground state is relatively small.

Comparing the benzene molecule in the linearly polarized and unpolarized cavities, respectively, reveals some differences (see Figures 4 and 5). First of all, the overall trends of the linearly polarized cavity agree with the results presented in ref 64. In particular, it is found that electron density is accumulated in the π-system for a polarization vector perpendicular to the molecule. When comparing to the results for unpolarized cavities, one has to keep in mind that the resulting point groups may differ: For linearly polarized cavities, the *D*_6*h*_ symmetry is preserved for a polarization vector **ϵ** oriented perpendicular to the molecular plane. Aligning the polarization vector within the molecular plane reduces the symmetry to *D*_2*h*_. This is contrary to the unpolarized cavity, where the *D*_6*h*_ orientation is preserved for the polarization vectors lying in the molecular plane (and ***k*** perpendicular to it). Thus, even if the systems have the same point-group symmetry, they describe different physical situations. Furthermore, comparing the density differences Δρ in Figures 4 and 5 reveals that the density in an unpolarized cavity is more strongly affected as compared to the linearly polarized case. This is mainly due to the fact, that the molecule is coupled by two perpendicularly polarized modes to the electric field, so each electron interacts with the EM field in two Cartesian directions, which increases the overall influence of the cavity on the molecule.

**Figure 5:**
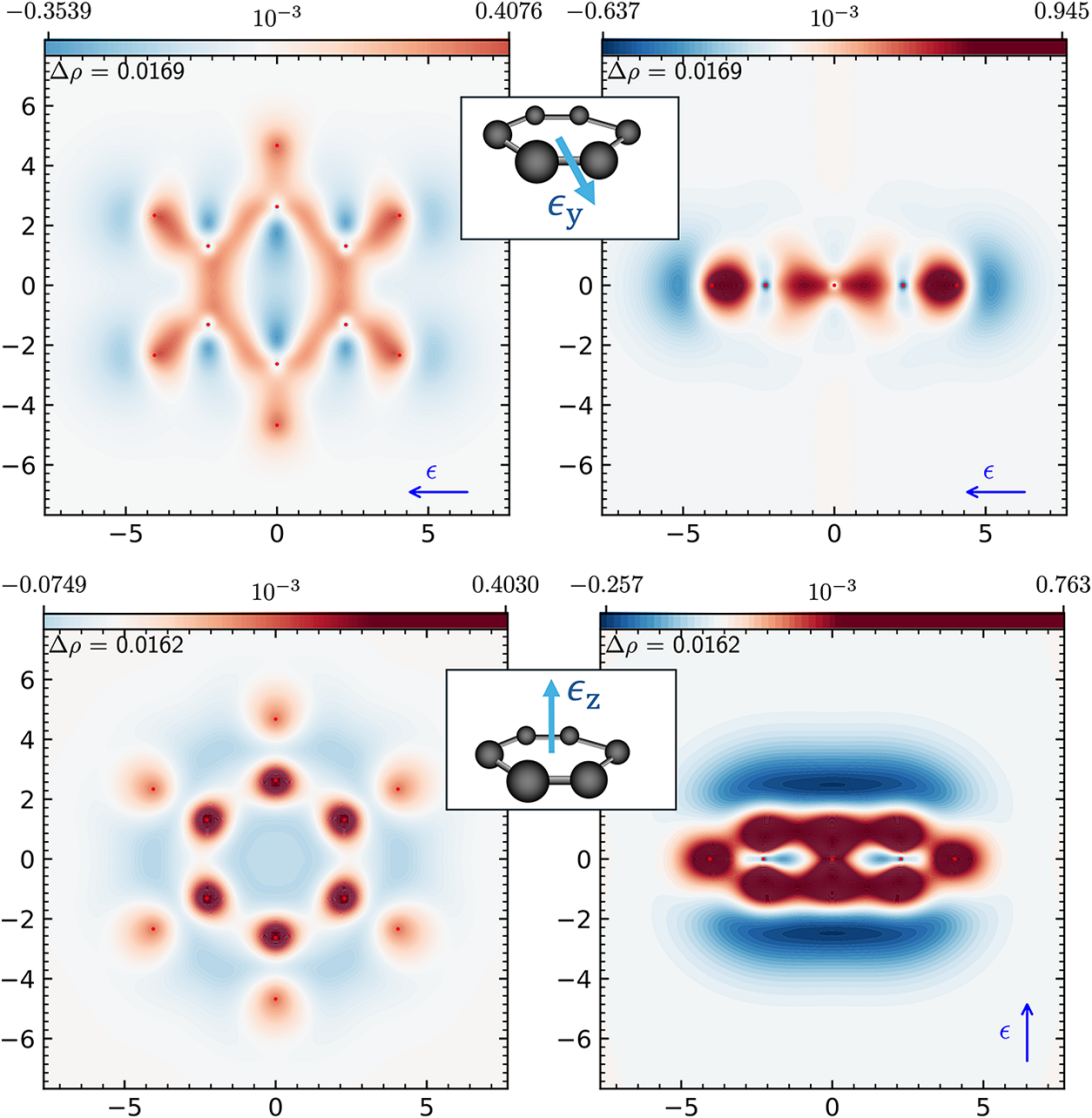
Correlated one-electron density differences for benzene in a linearly polarized cavity. Left and right plots are different perspectives on the same density difference. The upper plots show the **ϵ** vector aligned in the molecular plane and the lower plots show the **ϵ**-vector normal to the molecular plane. All QED-CCSD-12-SD calculations were performed using a cc-pVTZ basis, with a cavity frequency of ω_cav_ = 0.2 *E*_h_ and a coupling strength of λ = 0.2 au.

### 3.2 Fluorobenzene

For the fluorobenzene molecule, qualitative differences as compared to benzene are found: Figure 6 shows that the cavity can also be responsible for removing electrons from a bond, here the F–C bond. Hence, both accumulation and depletion of electron density in bonding regions may occur. The density shifts are mainly perpendicular to the ***k***-vector along the polarization vectors as can be seen by comparing the case where the ***k***-vector is perpendicular to the molecular plane and where the ***k***-vector lies within the molecular plane. If the ***k***-vector is aligned within the molecular plane and parallel to the F–C bond (hence the polarization vectors are perpendicular to the bond) the electron density in the bond is almost unaffected. When the ***k***-vector is perpendicular to the F–C bond (hence one polarization vector can be aligned parallel to it) the electron density in the bond is significantly reduced. A similar behavior is encountered in a linearly polarized cavity when the polarization vector is oriented along the F–C bond (see SI, Figure S1).

**Figure 6:**
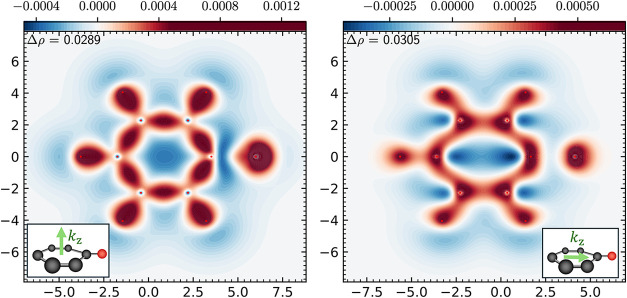
Correlated one-electron density differences for fluorobenzene in two orientations within an unpolarized cavity. The QED-CCSD-12-SD calculations were performed with a cc-pVTZ basis, with a cavity frequency of ω = 0.2 *E*_h_, and a coupling strength of λ = 0.05 au.

### 3.3 Azulene

The azulene molecule has been investigated at the QEDFT level of theory for a linearly polarized cavity by Flick et al.^21^ This allows for a comparison between QED-CC and QEDFT predictions. Figure 7 depicts the azulene molecule in a linearly (left) and unpolarized cavity (right) for a coupling strength of λ = 0.08 au and a frequency of ω = 2.41 eV. These parameters are consistent with those used in ref 21, except for the molecular geometry, which may differ slightly. Furthermore, the cc-pVDZ basis was employed. Flick et al. presented that the azulene molecule develops a rich fine structure in the one-electron density differences within a linearly polarized cavity. At a first glance, the density difference for the linearly polarized cavity appears similar to the results presented at the density functional theory (DFT) level of theory, in particular for the results obtained for the Krieger-Li-Iafrate approximation in ref 21. Most of the electron density is accumulated at the hydrogen atoms oriented along the polarization vector to both ends of the azulene molecule and the σ C–C bonds. We do not find an accumulation of electron density in the α position in the heptatrien ring as suggested by the DFT results with an optimized-effective potential (OEP), see also ref 21.

**Figure 7:**
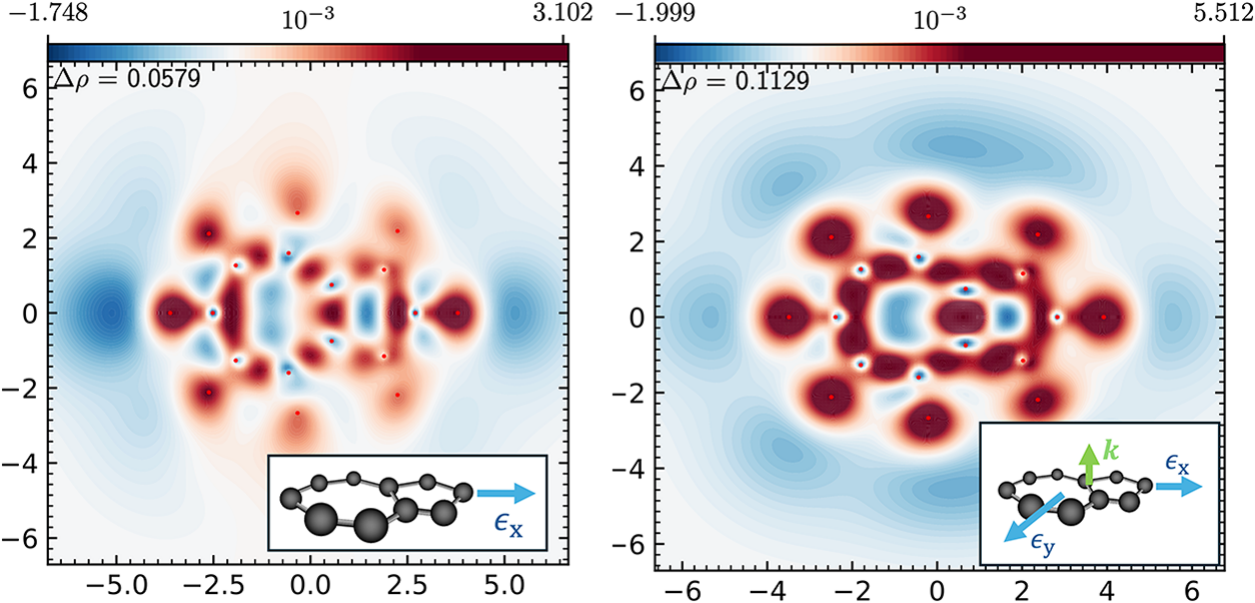
Correlated one-electron density differ- ences for the azulene molecule in a linearly polarized (left) and unpolarized cavity (right), respectively. The cavity frequency was set to ω = 2.41 eV, and the coupling strength λ = 0.08 au.

As expected, in an unpolarized cavity, see Figure 7 right, where the ***k***-vector of the cavity is aligned perpendicular to the molecule with the two polarization vectors lying in the molecular plane, the electron density is shifted in a symmetric manner and localized at the hydrogen atoms and the C–C σ bonds.

### 3.4 Symmetries of Excited States within Polaritonic EOM-CC Theory

In the uncoupled system (λ_α_ = 0) the excitation operator *R̂* yields in the simplest case either an electronic excitation *R̂*_μ_|0,0⟩ = |μ,0⟩ or a photonic creation *R̂*_ν_ |0,0⟩ = |0,ν⟩. In the following, the excited states are referred to by the irreducible representation they belong to. The electronic ground state corresponds to the Fermi vacuum with symmetry Γ_*G*_, while the photonic ground state corresponds to the physical vacuum and is hence totally symmetric |0,0⟩ = |Γ_*G*_,Γ_1_⟩. This scheme can be adapted for electronic and photonic excited states. For the electronic states we have |μ,0⟩ = |Γ_*E*_,Γ_1_⟩. For the photonic excited states two notations are used addressing either the number of photons or the irreducible representation of the photonic mode. I.e., an excitation with one photon will be denoted as |0,ν⟩ = |Γ_*G*_, 1⟩ or as |0,ν⟩ = |Γ_*G*_,Γ_α_⟩, respectively. Increasing the coupling strength mediates interactions via the bilinear coupling operator and leads to the formation of an upper and lower polariton |*P*_+_⟩ and |*P*_–_⟩. In the simplest case of just two interacting states, the upper and lower polaritons are given by a linear combination of the uncoupled states

$$\begin{array}{l} \left|P_{+}\right\rangle =c_{1}\left|\Gamma_{E},\Gamma_{1}\right\rangle +c_{2}\left|\Gamma_{G},\Gamma_{\alpha }\right\rangle \\ \left|P_{-}\right\rangle =c_{3}\left|\Gamma_{E},\Gamma_{1}\right\rangle -c_{4}\left|\Gamma_{G},\Gamma_{\alpha }\right\rangle \end{array}$$

The coupling occurs if the uncoupled electronic excited state |Γ_*E*_,Γ_1_⟩ and the cavity excitation |Γ_*G*_,Γ_α_⟩ belong to the same irreducible representation. The states hence only couple if Γ_1_ ∈ Γ_*G*_ ⊗ Γ_α_ ⊗ Γ_*E*_. The coefficients *c*_*i*_ are heavily influenced by the frequency and coupling strength of the cavity. The formation of the upper and lower polariton can be viewed as the result of an avoided crossing of an electronic excited state and an excitation of the EM field.^70,71^ Of course, if more states of the same irreducible representation are in the energetic vicinity of the cavity frequency, they may contribute to the interaction.

Note that although the notation |Γ_*G*_,0⟩ suggests the absence of photons, the similarity-transformed Hamiltonian still contains the exponential of the cluster operator, e^*Q̂*^. Hence, with the definition of the EOM excitation operator *R̂* from eq 6, the excited state within polaritonic CC theory is given as

$$\left|\Psi_{\mathrm{exc}}\right\rangle =e^{\mathrm{Q̂}}\mathrm{R̂}\left|\Gamma_{G},0\right\rangle =e^{\mathrm{Q̂}}\left|\Gamma_{E},\Gamma_{\alpha }\right\rangle$$

An exemplary energy diagram for the formation of polaritons in a three level system of electronic ground state *G*, and excited states *E*_1_ and *E*_2_ is shown in Figure 8. Note that for each electronic state |Γ_*E*_,0⟩, there exists one photonic state |Γ_*E*_,1⟩ shifted in energy by the cavity frequency ω_α_. Only the photonic state |Γ_*G*_,1⟩ is depicted, while in theory also the photonic states |Γ_*E*_1__,1⟩ and |Γ_*E*_2__,1⟩ exist, but are assumed to be energetically too high for any significant coupling.

**Figure 8:**
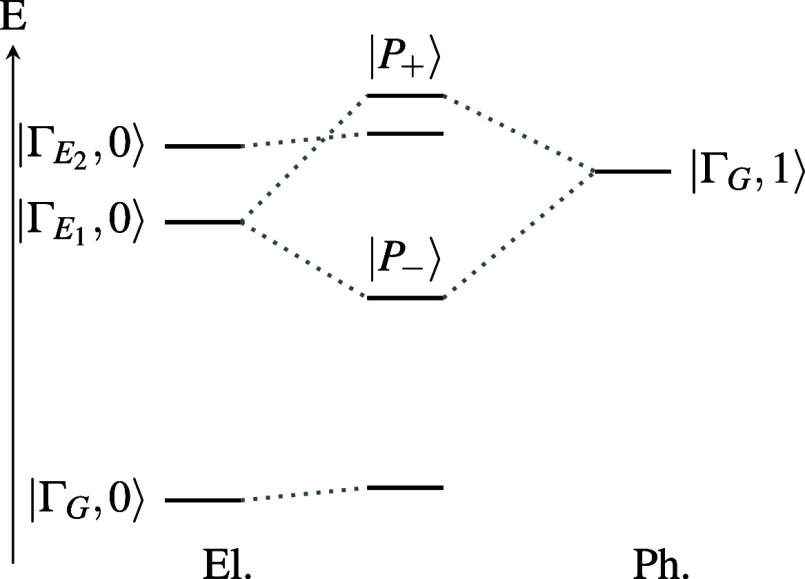
Exemplary energy landscape for an electronic three level system in the presence of a single photonic mode. The irreducible representations of the electronic ground state is Γ_*G*_ and for the two excited states Γ_*E*_1__ and Γ_*E*_2__. The irreducible representation of the photon is Γ_α_. For the depicted system Γ_*G*_ ⊗ Γ_α_ = Γ_*E*_1__ ≠ Γ_*E*_2__.

### 3.5 H_2_ in Linearly Polarized and Unpolarized Cavities

In the following we study the effects of a linearly polarized and an unpolarized cavity on the H_2_ molecule. We note that the polaritonic ground state does not change significantly in the cavity^17,25^ and hence we only discuss the polaritonic excited states. The following discussion is divided into states of *ungerade* and *gerade* parity, as they differ in their coupling behavior to the cavity. All calculations have been performed with the QED-EOM-CCSD-12-SD truncation scheme using an uncontracted (unc) augmented (aug) cc-pV5Z basis set.

#### 3.5.1 States of *Ungerade* Parity

The three lowest electronic excited states of *ungerade* parity of H_2_ in a linearly polarized cavity are depicted in Figure 9 as a function of the bond distance (mid and right). For reference, the left panel shows the excited states in absence of the cavity. To estimate the excitation of the cavity, the ground-state curve shifted by the cavity frequency ω_cav_ is included as a gray dashed line. The mid panel shows the case where the polarization vector is aligned parallel to the molecular axis (ϵ_∥_) and the right panel shows the case where the polarization vector is aligned perpendicular to the molecular axis (ϵ_⊥_). If the polarization vector is aligned parallel to the molecule, the overall symmetry of the system is *D*_∞*h*_ and the creation of a photon is Σ_*u*_^+^ symmetric. The cavity thus couples to both electronic excited states which are of Σ_*u*_^+^ symmetry (green values) and two upper and lower polaritons are formed, appearing at a bond distance of around 1.5 a_0_ and 2.3 a_0_. The electronic Π_*u*_ state (red values) is not strongly coupled to the cavity due to the different symmetry and hence crosses the photonic states. If the polarization vector is aligned perpendicular to the molecule (right), the symmetry of the system is reduced to *D*_2*h*_ and the creation of a photon is of *B*_3*u*_ symmetry (red values). The electronic Π_*u*_ state separates into the states with the symmetry *B*_2*u*_ and *B*_3*u*_ (red and orange), and thus only the *B*_3*u*_ state is strongly coupled to the cavity. Hence, only a single upper and lower polariton is formed, appearing at a bond distance of around 1.7 a_0_. The electronic *B*_1*u*_ states (green) are not strongly influenced by the cavity and develop parallel to the cavity free case as a function of the nuclear distance.

**Figure 9:**
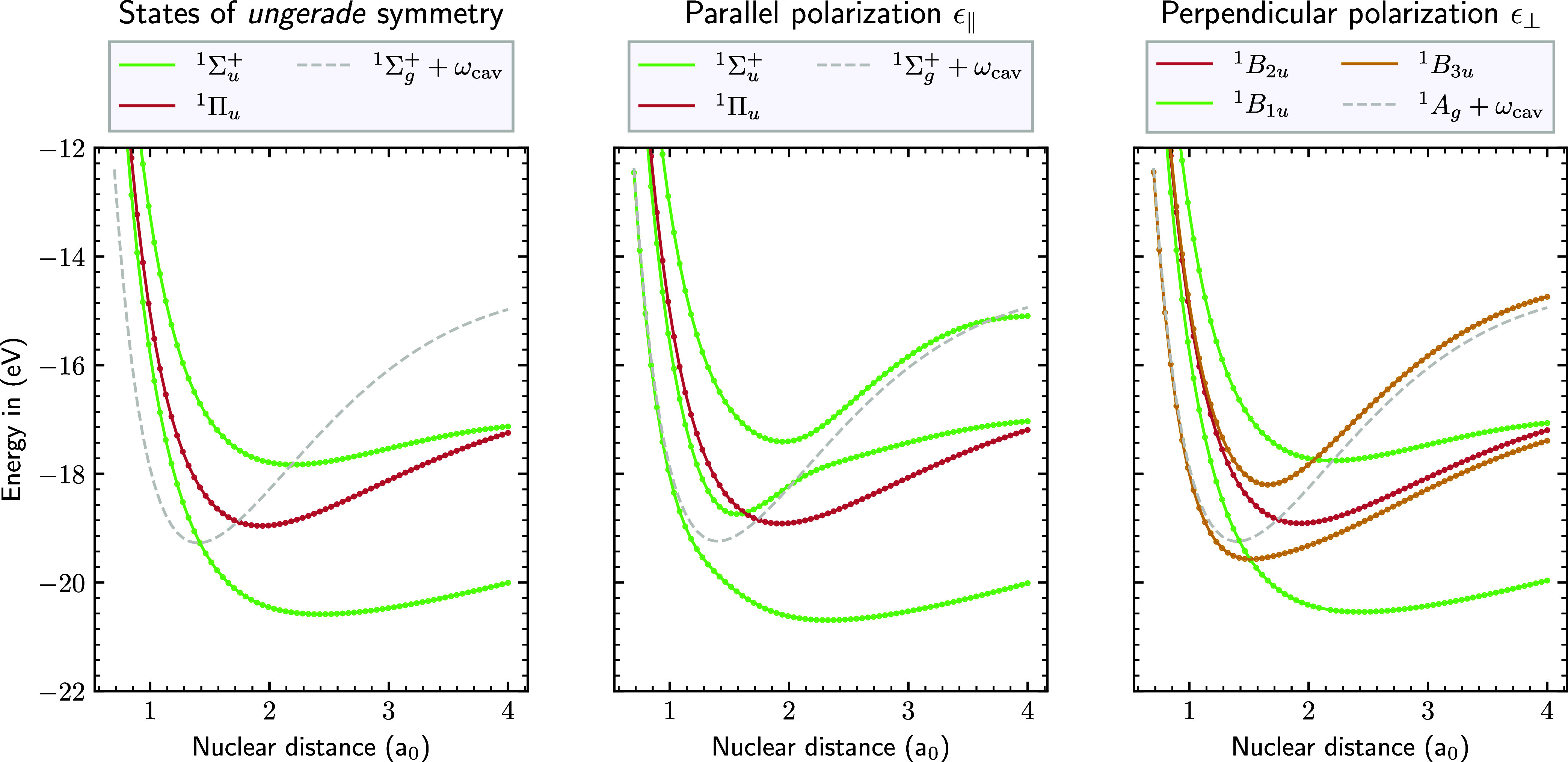
Low-lying singlet states of H_2_ in a linearly polarized cavity with the polarization vector aligned parallel (mid) and perpendicular (right) with respect to the molecular axis. For reference, the left panel shows the excited states in absence of the cavity. Only selected states of *ungerade* parity are shown. The cavity frequency was set to 12.48 eV and the coupling strength to 0.05 au. All QED-CCSD-12-SD calculations were performed with an unc-aug-cc-pV5Z basis.

The same electronic states for the hydrogen molecule in an unpolarized cavity are shown in Figure 10. Again, the left panel shows the hydrogen molecule in the absence of the cavity with the shifted ground state represented as a gray dashed line. The mid panel shows the case where the wave vector of the cavity is aligned parallel to the molecular axis (*k*_∥_) and the right panel shows the case where the wave vector is aligned perpendicular to the molecular axis (*k*_⊥_). In the parallel case, the creation of a photon is 2-fold degenerate and of Π_*u*_ symmetry (red values). Hence, a 2-fold degenerate upper and lower polariton is formed together with the electronic Π_*u*_ state.

**Figure 10:**
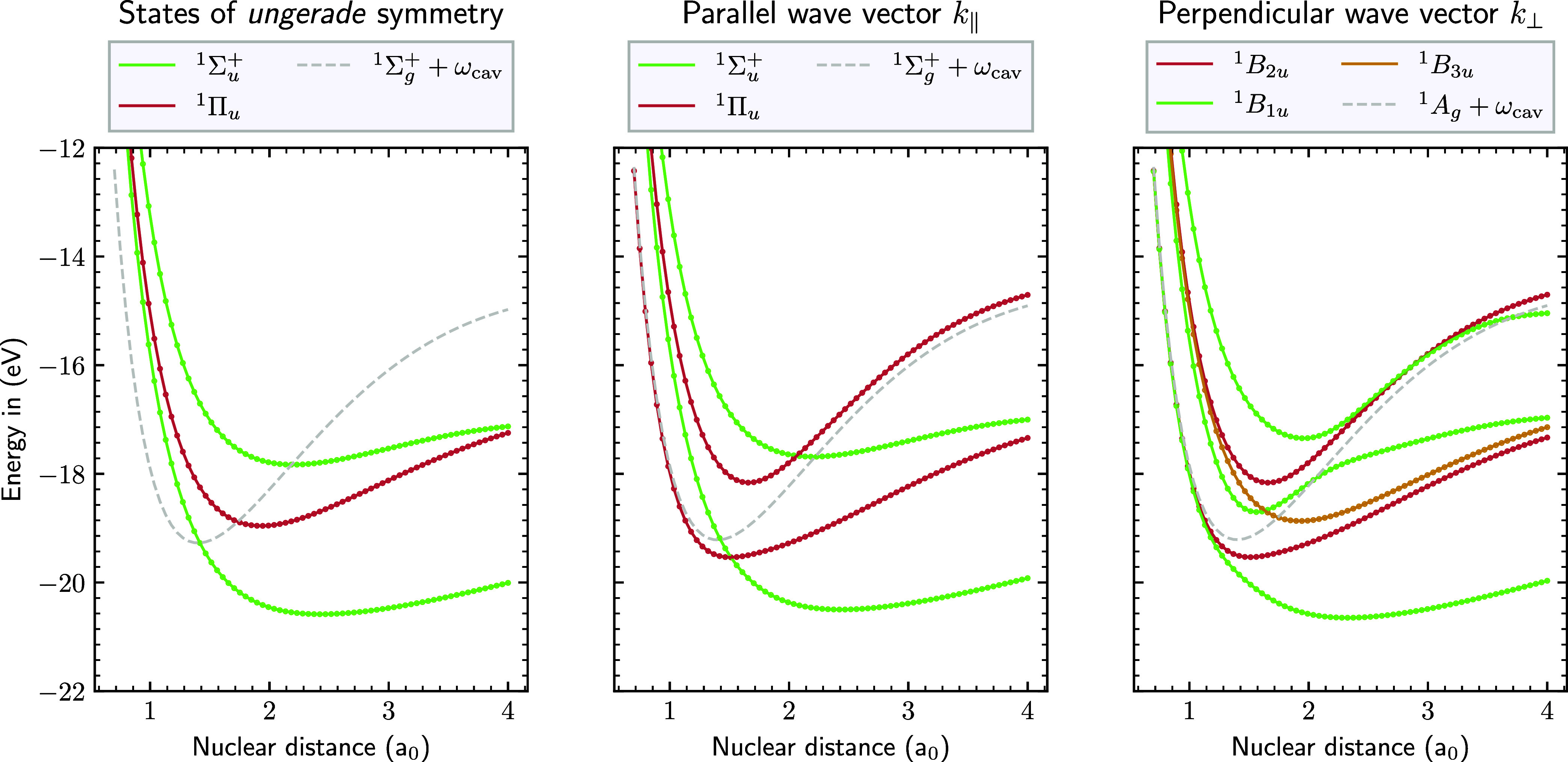
Low-lying singlet states of H_2_ in an unpolarized cavity with the polarization vector aligned parallel (mid) and perpendicular (right) with respect to the molecular axis. For reference, the left panel shows the excited states in absence of the cavity. Only selected states of *ungerade* parity are shown. The cavity frequency was set to 12.48 eV and the coupling strength to 0.05 au. All QED-CCSD-12-SD calculations were performed with an unc-aug-cc-pV5Z basis.

This degeneracy heavily relies on the fact that the coupling strengths along the two polarization directions are identical, i.e., λ_1_ = λ_2_, which is the case for an ideal unpolarized cavity. In the SI, we show that a small deviation from this ideal case, i.e., λ_1_ = 0.95 λ_2_, lifts the degeneracy and results in a small separation of the upper and lower polariton (≈ 0.02 eV). The qualitative picture of the excited-state landscape, however, remains unchanged.

If the molecule is aligned perpendicular to the wave vector, the *D*_∞*h*_ symmetry is reduced to *D*_2*h*_ and the degeneracy of the electronic and photonic states is lifted. The electronic state separates as Π_*u*_ → *B*_2*u*_ ⊕ *B*_3*u*_ and the photonic state as Π_*u*_ → *B*_*1u*_ ⊕ *B*_2*u*_. Thus, for the perpendicular orientation, two upper and lower polaritons are formed, one within the *B*_1*u*_ states (green) and the other within the *B*_2*u*_ states (red).

Comparing the results of the linearly polarized and unpolarized cavity, respectively, shows that a fundamentally different excited-state landscape is obtained for the two cases. While in the linearly polarized cavity only one avoided crossing is observed, the unpolarized cavity exhibits two avoided crossings. We note that the excited states of the molecule in an unpolarized cavity with the wave vector aligned perpendicular to the molecular axis (*k*_⊥_) appear as the combined result of the two linearly polarized cavities, i.e., ϵ_∥_ and *ε*_⊥_. This behavior is reasonable, since the two field polarizations contribute additively in the Hamiltonian. Nevertheless, various molecular properties, including the features of the absorption spectra, nuclear dynamics, and thermodynamic behavior of the excited states, are expected to be fundamentally altered in the two cavity types due to the distinct excited-state landscapes and the topology of the energy surfaces.

#### 3.5.2 States of *Gerade* Parity

The states of *gerade* parity differ from those of *ungerade* parity as the formation of the upper and lower polariton cannot result from a single-photon excitation with respect to the polaritonic ground state |Σ_*g*_^+^, 00̅⟩. As the coupled light-matter system exhibits an inversion symmetry, the excitation of a single photon changes the parity of the system. Hence, to couple electronic states of *gerade* parity to the photon field, two-photon excitations are required. The description within the EOM calculation therefore must include the two-photon creation operator *R̂*_0_^2^, see also eq 6. Figure 11 shows the lowest-lying states of *gerade* parity for the linearly polarized cavity. The cavity frequency was tuned to 6.694 eV which is about half the excitation energy of the first electronic excited states of *gerade* parity. The left panel shows the hydrogen molecule in absence of the cavity with the shifted ground state as a gray dashed line (shifted by 2ℏω_cav_). In total, three electronic states of Σ_*g*_^+^ symmetry are shown (blue) and one degenerate state with Π_*g*_ symmetry (red). If the molecule is aligned parallel to the polarization vector (mid panel), the creation of a photon is of Σ_*u*_^+^ symmetry and thus the creation of two photons corresponds to Σ_*g*_^+^ symmetry.[Fn fn3] Therefore, all three electronic states of Σ_*g*_^+^ symmetry are coupled to the two-photon state. The first and second avoided crossings are relatively weak, whereas the avoided crossing with the third state is considerably stronger. The electronic state with Π_*g*_ symmetry (red) is not strongly coupled to the cavity and is allowed to cross with the photonic states. If the molecule is aligned perpendicularly to the polarization vector (right panel), the symmetry is reduced to *D*_2*h*_ and the degeneracy in the electronic state is lifted as Π_*g*_ →*B*_2*g*_ ⊕ *B*_3*g*_ (orange and red). However, the two-photon state is still of *A*_*g*_ symmetry and couples to the corresponding electronic states (blue). It should be noted that the third avoided crossing is significantly less pronounced in the perpendicular orientation than in the parallel orientation.

**Figure 11:**
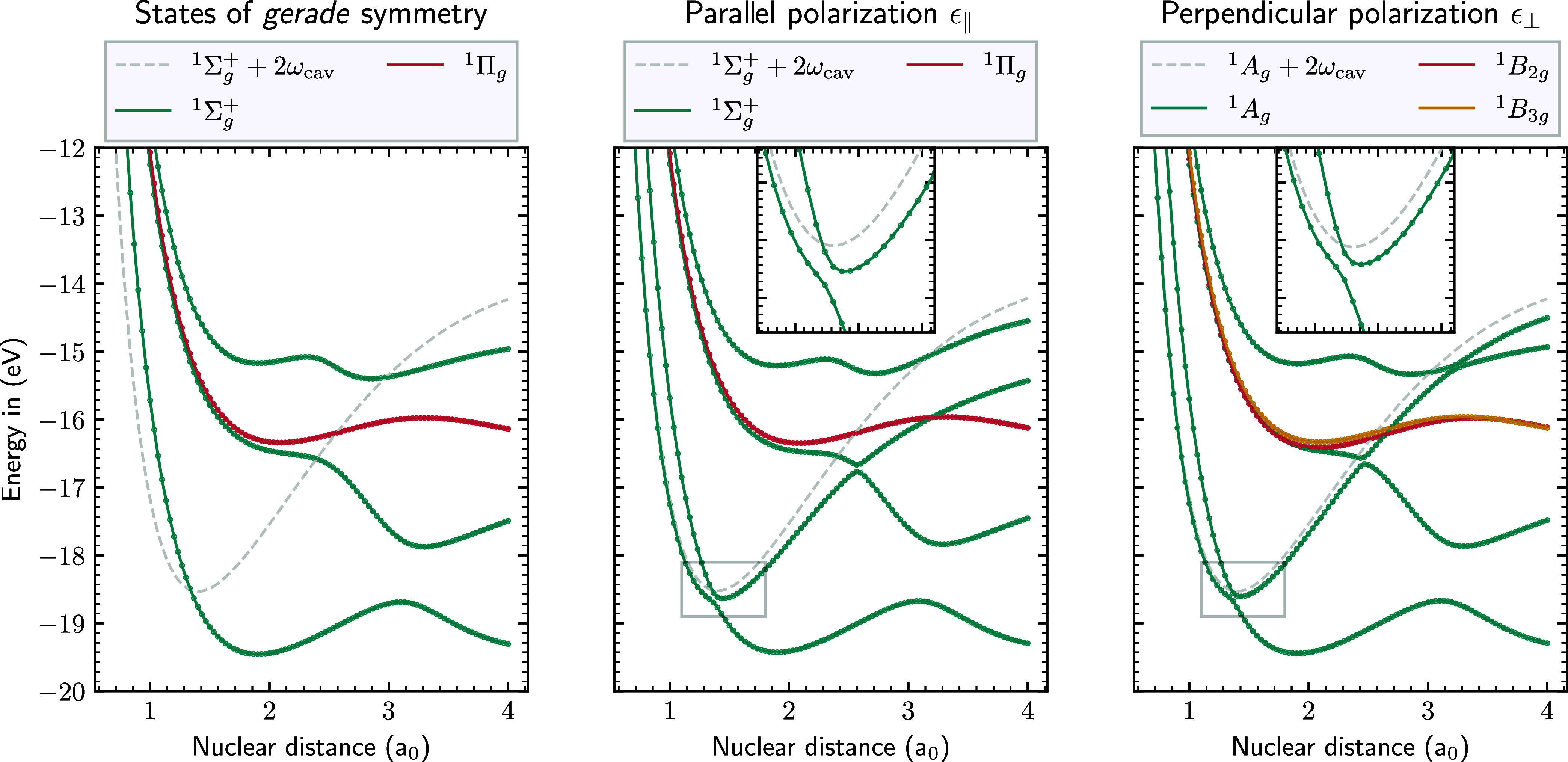
Low-lying singlet states of H_2_ in a linearly polarized cavity with the polarization vector aligned parallel (mid) and perpendicular (right) with respect to the molecular axis. For reference, the left panel shows the excited states in absence of the cavity. Only selected states of *gerade* parity are shown. The cavity frequency was set to 6.694 eV and the coupling strength to 0.05 au. All QED-CCSD-12-SD calculations were performed with an unc-aug-cc-pV5Z basis.

In Figure 12 the lowest-lying states of *gerade* parity are shown for the unpolarized cavity. In the unpolarized cavity the creation of a single photon is two-fold degenerate and of Π_*u*_ symmetry (with the molecule aligned parallel to the wave vector). Hence, the creation of two photons can be determined as Π_*u*_ ⊗ Π_*u*_ = Σ_*g*_^+^ ⊕ Σ_*g*_^–^ ⊕ Δ_*g*_. However, the photonic state must be symmetric with respect to the exchange of two particles, which requires the antisymmetric state Σ_*g*_^–^ to be excluded. The two-photon states can therefore be expressed as an excitation of the ground-state |Σ_*g*_^+^,00̅⟩ with the following two-photon excitations

$$\begin{array}{l} \Sigma_{g}^{+}\to \left|\Sigma_{g}^{+},2\mathrm{0̅}\right\rangle +\left|\Sigma_{g}^{+},0\mathrm{2̅}\right\rangle \\ \Delta_{g}^{\left(1\right)}\to \left|\Sigma_{g}^{+},1\mathrm{1̅}\right\rangle \\ \Delta_{g}^{\left(2\right)}\to \left|\Sigma_{g}^{+},2\mathrm{0̅}\right\rangle -\left|\Sigma_{g}^{+},0\mathrm{2̅}\right\rangle \end{array}$$

The symmetry of the photonic states can be identified from the character tables by considering the transformation of the quadrupole moment, i.e., if the wave vector is aligned along the *z*-axis, the two-photon states transform as *xx*, *yy*, and *xy* components.^45^ Due to the symmetry, only the Σ_*g*_^+^ photonic state is strongly coupled to the electronic states (blue) while the photonic Δ_*g*_ state (green) crosses the electronic states. Similarly, also the electronic Π_*g*_ state (red) has a different symmetry and is allowed to cross with the photonic states. If the molecule is oriented perpendicular to the wave vector (right panel), the degenerate photonic states separates as Δ_*g*_ → *A*_*g*_ ⊕ *B*_3*g*_ and with respect to the polaritonic ground-state |Σ_*g*_^+^, 0 0̅⟩, the two-photon states can be expressed as

$$\begin{array}{l} A_{g}\to \left|\Sigma_{g}^{+},2\mathrm{0̅}\right\rangle \\ A_{g}\to \left|\Sigma_{g}^{+},0\mathrm{2̅}\right\rangle \\ B_{3g}\to \left|\Sigma_{g}^{+},1\mathrm{1̅}\right\rangle \end{array}$$

Hence, in total two two-photon states have *A*_*g*_ symmetry and both are coupled to the electronic states of *A*_*g*_ symmetry (blue). It is pointed out that one of the two-photon states with a *A*_*g*_ symmetry is significantly more strongly coupled to the electronic states. The *B*_3*g*_ photonic state (orange), which has one photon in each polarization (|Σ_*g*_^+^,11̅⟩), undergoes an avoided crossing with the electronic state of the same symmetry. Note that this avoided crossing appears only in the unpolarized cavity and is not observed in the linearly polarized cavity, regardless of the molecular orientation. The only state that does not exhibit an avoided crossing is the electronic *B*_2*g*_ state (red).

**Figure 12:**
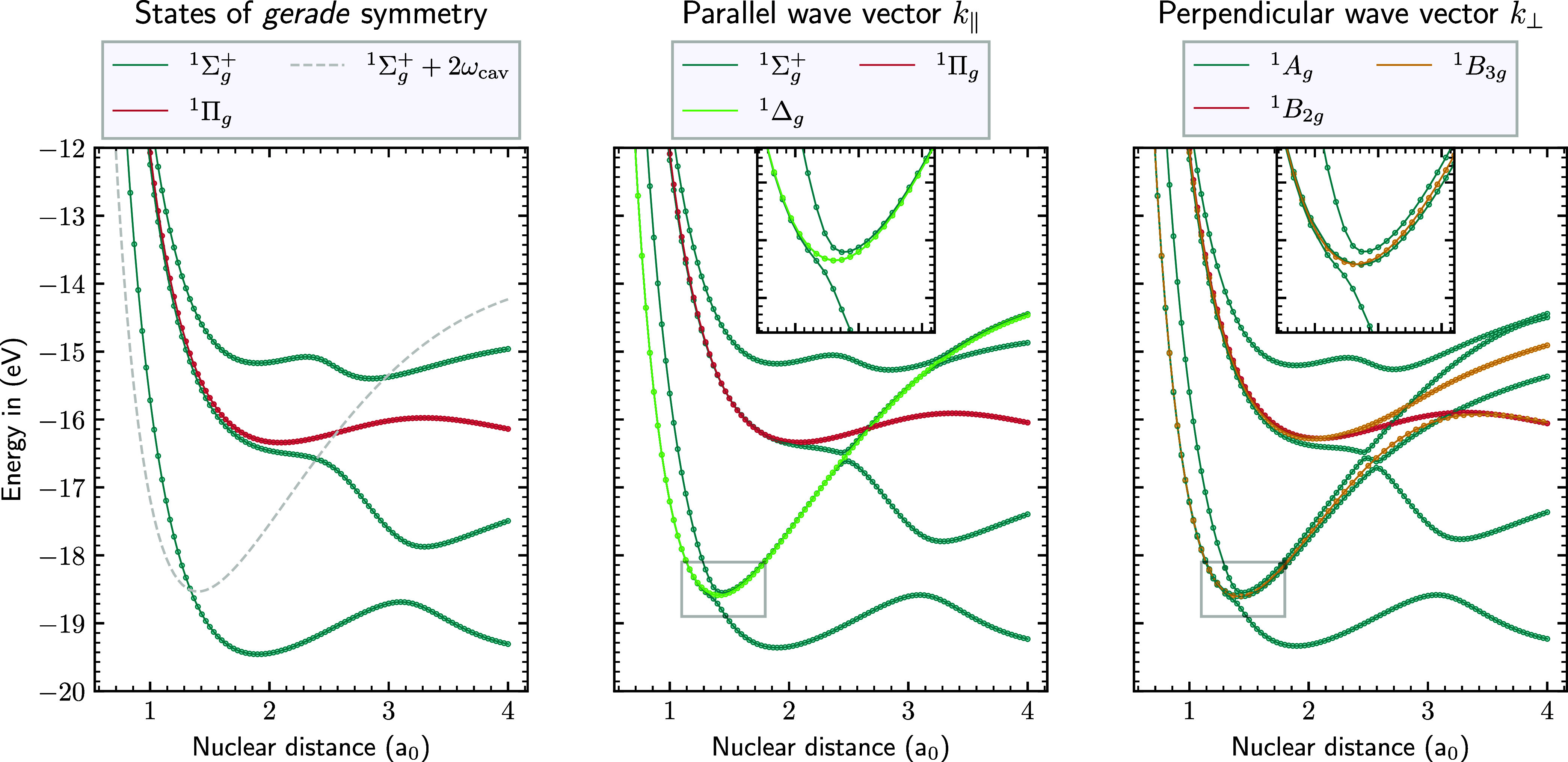
Low-lying singlet states of H_2_ in an unpolarized cavity with the polarization vector aligned parallel (mid) and perpendicular (right) with respect to the molecular axis. For reference, the left panel shows the excited states in absence of the cavity. Only selected states of *gerade* parity are shown. The cavity frequency was set to 6.694 eV and the coupling strength to 0.05 au. All QED-CCSD-12-SD calculations were performed with an unc-aug-cc-pV5Z basis.

The results on the excited states of the H_2_ molecule illustrate that the linearly and unpolarized cavity will lead to fundamentally different excited-state landscapes. In particular, the molecular orientations at which the upper and lower polaritons are formed can vary substantially between the two cavity types. For instance, the unpolarized cavity has a more complex excited-state landscape due to the two polarization directions along which the cavity couples to the matter system,. We reiterate that the results presented here correspond to an idealized system with equal coupling strengths for both polarization directions. In an experimental setup, the system may not preserve, for example, the *D*_∞*h*_ symmetry, which could lift the degeneracy of the photonic states. Similarly, due to imperfections, a linearly polarized cavity might support a second field polarization that is weakly coupled to the matter system, leading to a lifting of degeneracy as well. Nevertheless, the present calculations illustrate the fundamental differences between the two idealized cavity types.

## 4 Conclusions

In this paper, we presented a generalization of polaritonic coupled-cluster (CC) theory to account for a rotationally symmetric, unpolarized cavity within the dipole approximation, such as an unpolarized Fabry-Pérot cavity. To maintain the *D*_∞*h*_ symmetry of the bare cavity, at least two perpendicular polarized modes must be included in the Hamiltonian.

Current implementations within QED-CC have been limited to the case of polarized cavities. The presented generalization to unpolarized cavities allows for a wider range of cavity types to be investigated and can be used to study the influence of the cavity on molecular properties in a more general setting. Calculations on the aromatic species benzene, fluorobenzene, and azulene showed that the changes in the QED-CC one-electron density vary significantly between a linearly polarized and an unpolarized cavity. For the fluorobenzene molecule, we have also shown that placing a molecule in a cavity can remove electron density from the fluor-carbon bond, which might lead to the destabilization of the chemical bond. This mechanism could potentially influence the reactivity of the molecule. Furthermore, for the one-electron density of the azulene molecule, QED-CC calculations were compared to QEDFT results. These have revealed some agreement in the qualitative shift of the one-electron density, but also show clear differences which should be investigated further.

An efficient exploitation of point-group symmetry based on the direct product decomposition has been presented which leads to a significant speed up of the QED-CC and QED-EOM-CC calculations. It also allows the characterization of excited states according to the irreducible representations and allows to predict between which states a Rabi splitting is formed.

Studies on the H_2_ molecule showed rich coupling patterns, where the exploitation of point-group symmetry allowed the characterization of polaritonic excited states and enabled their targeted calculation. It has also been shown for the H_2_ molecule that the unpolarized cavity can be qualitatively understood as effectively representing a combination of two orthogonal linear polarizations.

Future work should investigate the effects of mass renormalization and how the matter system is modified when additional cavity modes are included. Moreover, exploring a continuous tuning of the cavity from a linearly polarized to an unpolarized configuration, by gradually varying the coupling strength of the second polarization direction, could provide further insight into how cavity polarization influences molecular properties.

## Supplementary Material



## Data Availability

The data that support the findings of this study are available within the article and its Supporting Information.

## Footnotes

1 For electronic operators, the transformation can be found in the literature, see, for example, refs 45 and 46.

2 Note that the same is not true for a molecule in a linearly polarized cavity.

3 The symmetry of the two-photon state is obtained from the direct product Σ_*u*_^+^ ⊗ Σ_*u*_^+^ = Σ_*g*_^+^.

## A Transformation to Complex Polarization Vectors

It will be illustrated that the cavity is neither linearly or circularly polarized by transforming the displacement field ***D* ^** in a basis of complex polarization vectors. Using the unitary operator

$${Û}_{\varphi }=\exp\left[i\varphi \sum_{\alpha }^{N_{\mathrm{cav}}}\left({\mathrm{b̂}}_{\alpha }^{†}{\hat{\mathrm{b̅}}}_{\alpha }+{\hat{\mathrm{b̅}}}_{\alpha }^{†}{\mathrm{b̂}}_{\alpha }\right)\right]$$

the elementary operators can be transformed as

7
$$\begin{array}{l} {\mathrm{b̂}}_{\alpha }\to {\mathrm{b̂}}_{\alpha }\cos\varphi +i{\hat{\mathrm{b̅}}}_{\alpha }\sin\varphi \\ {\hat{\mathrm{b̅}}}_{\alpha }\to {\hat{\mathrm{b̅}}}_{\alpha }\cos\varphi +i{\mathrm{b̂}}_{\alpha }\sin\varphi \\ {\mathrm{b̂}}_{\alpha }^{†}\to {\mathrm{b̂}}_{\alpha }^{†}\cos\varphi -i{\hat{\mathrm{b̅}}}_{\alpha }^{†}\sin\varphi \\ {\hat{\mathrm{b̅}}}_{\alpha }^{†}\to {\hat{\mathrm{b̅}}}_{\alpha }^{†}\cos\varphi -i{\mathrm{b̂}}_{\alpha }^{†}\sin\varphi \end{array}$$

This corresponds to a transformation into a basis of elliptical polarization vectors and can be made explicit when inserting the elementary operators (eq 7) in the displacement field (eq 5). E.g. using the angle φ = π/2, the polarization vectors transform as

$$\begin{array}{l} \epsilon^{\prime }=\frac{1}{\sqrt{2}}\left(\epsilon +i\mathrm{\epsilon ̅}\right)\\ {\mathrm{\epsilon ̅}}^{\prime }=\frac{1}{\sqrt{2}}\left(\mathrm{\epsilon ̅}+i\epsilon \right) \end{array}$$

hence, they become circularly polarized. This illustrates that the polarization vectors only serve as a basis for the representation of the EM field. Whether real or complex valued polarization vectors polarization vectors are used does not change the underlying physical properties.

## Notes

Computational details together with molecular geometries for benzene, azulene, and fluorobenzene; it further provides one-electron density plots; potential barrier analyses under various cavity conditions; and comprehensive tables of excited states of H_2_ with both *gerade* and *ungerade* symmetry in linearly polarized and unpolarized cavities (PDF)
