# Supplementary material for: Polaritonic Coupled Cluster Theory for Unpolarized Cavities Exploiting Point-Group Symmetry
Source: J Chem Theory Comput. 2026 Jun 2;22(12):5991–6007. doi: 10.1021/acs.jctc.5c01343 (PMC13296504; doi:10.1021/acs.jctc.5c01343)
Supplement: Supplementary file 1 [file ct5c01343_si_001.pdf]

Supporting Information: Polaritonic Coupled Cluster Theory for Unpolarized Cavities Exploiting Point-Group Symmetry

Laurenz Monzel*,† and Stella Stopkowicz*,†,‡

†*Fachrichtung Chemie, Universität des Saarlandes, Campus B2.2, D-66123 Saarbrücken, Germany*

‡*Hylleraas Centre for Quantum Molecular Sciences, Department of Chemistry, University of Oslo, P.O. Box 1033 Blindern, N-0315 Oslo, Norway*

E-mail: laurenz.monzel@uni-saarland.de; stella.stopkowicz@uni-saarland.de

Rotational invariance of the unpolarized cavity

As the Hamiltonian will not change under a linear combination of the polarization vectors, the energy will not change in the QED-HF and QED-CC calculations up to the convergence criterion. The solvers are therefore not sensitive to the chosen basis of the polarization vectors. To demonstrate this point, a QED-CCSD-1-SD/cc-pV5Z calculation for the H_2O molecule was performed, with the wave vector aligned perpendicular to the molecular plane, and three linear combinations of the polarization vectors $(0°, 45°, 30°)$. For a convergence criterion in the electron density of 10^{-10}, all energies agree up to 10^{-13} E_{h}.

Table S1: Geometry for the water molecule.

	x (a_0)	y (a_0)	z (a_0)
H	0	-1.44597	1.10484
H	0	1.44597	1.10484
O	0	0.00000	0.00000

Table S2: Three orientation of the polarization vectors $\boldsymbol{\epsilon}$ an $\bar{\boldsymbol{\epsilon}}$.

	x (a_0)	y (a_0)	z (a_0)
		Case 1	
$\boldsymbol{\epsilon}$	0.0	0.0	-1.0
$\bar{\boldsymbol{\epsilon}}$	0.0	1.0	0.0
		Case 2	
$\boldsymbol{\epsilon}$	0.0	0.707107	-0.707107
$\bar{\boldsymbol{\epsilon}}$	0.0	0.707107	0.707107
		Case 3	
$\boldsymbol{\epsilon}$	0.0	0.5	-0.866025
$\bar{\boldsymbol{\epsilon}}$	0.0	0.866025	0.5

Table S3: Ground-state correlation energies for the water molecule and absolute energies.

	QED-CC (Corr. energy) (E_h)	QED-HF + QED-CC (E_h)
Case 1	-0.2928388191294 34	-76.308921992697 02
Case 2	-0.2928388191294 37	-76.308921992697 09
Case 3	-0.2928388191294 35	-76.308921992697 07

Fluorobenzene in a linearly polarized cavity

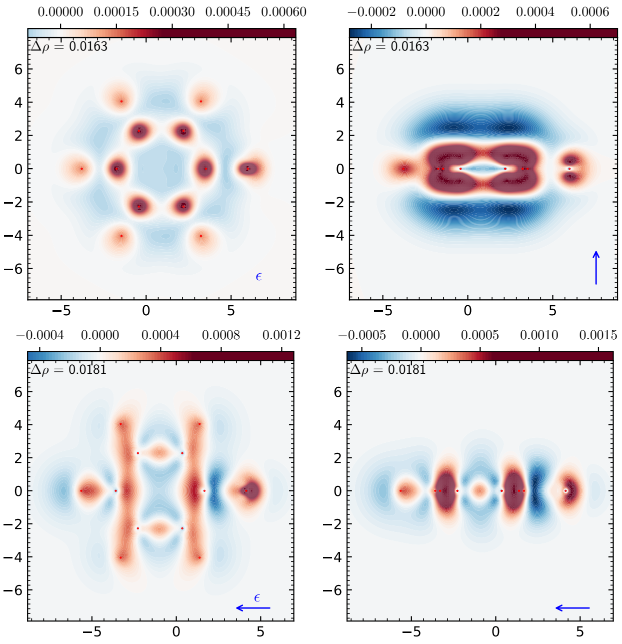

Figure S1: Correlated one-electron density differences for fluorobenzene in two orientations within a linearly polarized cavity. The QED-CCSD-12-SD calculations were performed with a cc-pVTZ basis, a cavity frequency of $\omega = 0.2\ E_\mathrm{h}$ and a coupling strength of $\lambda = 0.05$ a.u.

Benzene in a linearly and unpolarized cavity

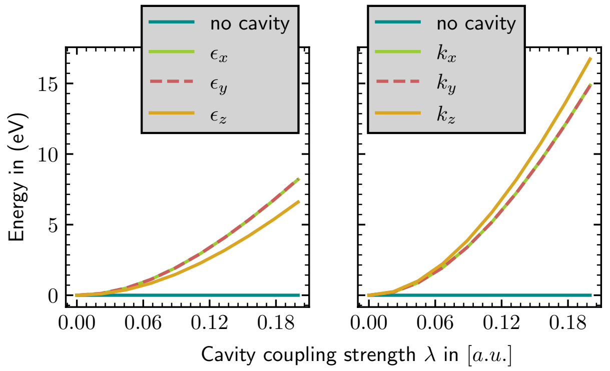

Figure S2: Potential barrier height of the benzene molecule with respect to different orientations in a linearly (left) and unpolarized (right) cavity. The cavity frequency was fixed as $\omega_{\text{cav}} = 0.1\ E_{\text{h}}$. The coupling strength was varied in steps of $\Delta\lambda = 0.022$ a.u. A cc-pVTZ basis was utilized.

Geometries of molecules

Table S4: Geometry for the benzene molecule in a_0.

Atom center	Cartesian geometry in a_0	
H	4.04983121690698	2.33848216048779
C	2.27711795932538	1.31460686449430
C	2.27711795932538	-1.31460686449430
H	4.04983121690698	-2.33848216048779
C	0.00000000000000	-2.62905343248059
H	0.00000000000000	-4.67606322887373
C	-2.27711795932538	-1.31460686449430
H	-4.04983121690698	-2.33848216048779
C	-2.27711795932538	1.31460686449430
H	-4.04983121690698	2.33848216048779
C	0.00000000000000	2.62905343248059
H	0.00000000000000	4.67606322887373

Table S5: Geometry for the azulene molecule in a_0.

Atom center	Cartesian geometry in a_0	
C	-1.26866806853591	1.92438989279682
C	-1.59699010340496	0.56261557814130
H	-2.66893282091365	0.33204619125468
C	-0.75099634964616	-0.54694244712311
C	-1.15211338363022	-1.89765202634847
C	0.75099634964616	-0.54694244712311
C	1.15211338363022	-1.89765202634847
C	1.59699010340496	0.56261557814130
H	2.66893282091365	0.33204619125468
C	1.26866806853591	1.92438989279682
H	2.11573594524611	2.61742512270539
C	0.00000000000000	-2.70561721020542
H	-2.11573594524611	2.61742512270539
H	-2.18544728279424	-2.24575344665612
H	2.18544728279424	-2.24575344665612
H	0.00000000000000	-3.79752591434090
C	0.00000000000000	2.51809656766395

H	0.00000000000000	3.61381384682047

Table S6: Geometry for the fluorobenzene molecule in a_0.

Atom center	Cartesian geometry in a_0	
F	0.00000000000000	-5.1000000000000
H	4.04983121690698	2.33848216048779
C	2.27711795932538	1.31460686449430
C	2.27711795932538	-1.31460686449430
H	4.04983121690698	-2.33848216048779
C	0.00000000000000	-2.62905343248059
C	-2.27711795932538	-1.31460686449430
H	-4.04983121690698	-2.33848216048779
C	-2.27711795932538	1.31460686449430
H	-4.04983121690698	2.33848216048779
C	0.00000000000000	2.62905343248059
H	0.00000000000000	4.67606322887373

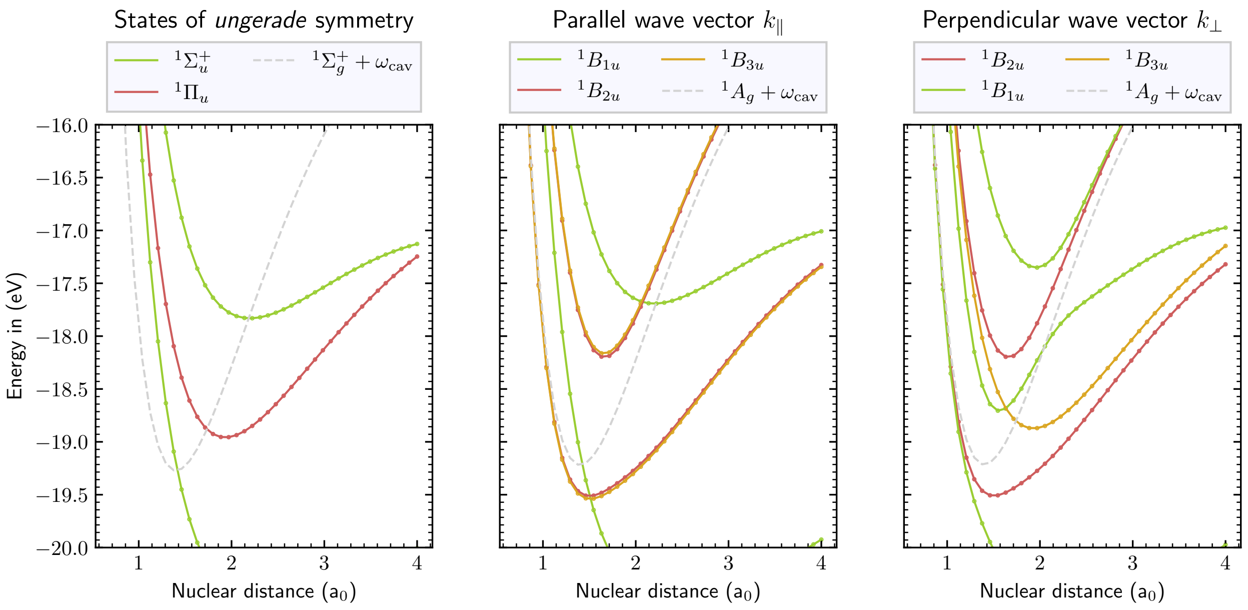

Figure S3: Low-lying singlet states of H_2 in an cavity with different coupling strengths along the polarization vectors ($\lambda_1 = 0.05$ a.u. and $\lambda_2 = 0.0475$ a.u.). The reference without the cavity (left), the wave vector aligned parallel (mid) and perpendicular (right) with respect to the molecular axis. Only selected states of *ungerade* parity are shown. The cavity frequency was set to 6.694 eV. All QED-CCSD-12-SD calculations were performed with an unc-aug-cc-pV5Z basis.

Excited states of the H_2 molecule

Table S7: Excited states of the H_2 molecule in a lineary polarized cavity with $\lambda = 0.05$ a.u. and $\omega = 0.466$ E_h and the polarization vector parallel to the molecular axis. States of *ungerade* parity. An unc-aug-pV5Z basis was used. Table contains all energies in units of $E_{\rm h}$.

R in (a_0)	$\epsilon_\parallel(\Sigma_u^+)$	$\epsilon_\parallel(\Sigma_u^+)$	$\epsilon_\parallel(\Sigma_u^+)$	$\epsilon_\parallel(\Pi_u)$
0.70000000	-0.41803520	-0.26429576	-0.17213860	-0.24512039
0.74782609	-0.47056533	-0.32820299	-0.23548150	-0.30860300
0.79565217	-0.51333472	-0.38192042	-0.28879393	-0.36189699
0.84347826	-0.54822172	-0.42730805	-0.33396898	-0.40687100
0.89130435	-0.57669428	-0.46581368	-0.37246619	-0.44498437
0.93913043	-0.59991614	-0.49857641	-0.40541846	-0.47739142
0.98695652	-0.61882292	-0.52649974	-0.43371782	-0.50501575
1.03478261	-0.63417782	-0.55030417	-0.45807919	-0.52860396
1.08260870	-0.64661337	-0.57056502	-0.47908439	-0.54876521
1.13043478	-0.65666324	-0.58773993	-0.49721274	-0.56600055

1.17826087	-0.66478640	-0.60218950	-0.51286297	-0.58072521
1.22608696	-0.67138461	-0.61419410	-0.52636920	-0.59328558
1.27391304	-0.67681212	-0.62397064	-0.53801286	-0.60397251
1.32173913	-0.68137633	-0.63169271	-0.54803195	-0.61303149
1.36956522	-0.68532894	-0.63751581	-0.55662860	-0.62067088
1.41739130	-0.68885339	-0.64160307	-0.56397506	-0.62706829
1.46521739	-0.69205949	-0.64414113	-0.57021842	-0.63237580
1.51304348	-0.69499385	-0.64533827	-0.57548420	-0.63672414
1.56086957	-0.69766265	-0.64540862	-0.57987929	-0.64022614
1.60869565	-0.70005523	-0.64455447	-0.58349427	-0.64297951
1.65652174	-0.70215992	-0.64295527	-0.58640532	-0.64506915
1.70434783	-0.70397122	-0.64076482	-0.58867571	-0.64656906
1.75217391	-0.70549110	-0.63811371	-0.59035687	-0.64754397
1.80000000	-0.70672776	-0.63511397	-0.59148907	-0.64805061
1.84782609	-0.70769369	-0.63186419	-0.59210177	-0.64813889
1.89565217	-0.70840384	-0.62845405	-0.59221369	-0.64785279
1.94347826	-0.70887427	-0.62496773	-0.59183311	-0.64723117
1.99130435	-0.70912116	-0.62148533	-0.59095906	-0.64630847
2.03913043	-0.70916020	-0.61808129	-0.58958492	-0.64511523
2.08695652	-0.70900617	-0.61481883	-0.58770570	-0.64367860
2.13478261	-0.70867281	-0.61174141	-0.58532798	-0.64202276
2.18260870	-0.70817265	-0.60886541	-0.58247873	-0.64016928
2.23043478	-0.70751708	-0.60617932	-0.57920715	-0.63813741
2.27826087	-0.70671635	-0.60365116	-0.57557836	-0.63594436
2.32608696	-0.70577964	-0.60123977	-0.57166272	-0.63360558
2.37391304	-0.70471517	-0.59890464	-0.56752665	-0.63113488
2.42173913	-0.70353027	-0.59661137	-0.56322764	-0.62854470
2.46956522	-0.70223145	-0.59433332	-0.55881296	-0.62584624
2.51739130	-0.70082453	-0.59205121	-0.55432040	-0.62304959
2.56521739	-0.69931471	-0.58975174	-0.54977992	-0.62016389
2.61304348	-0.69770666	-0.58742623	-0.54521527	-0.61719739
2.66086957	-0.69600455	-0.58506942	-0.54064539	-0.61415763
2.70869565	-0.69421219	-0.58267848	-0.53608561	-0.61105146
2.75652174	-0.69233303	-0.58025237	-0.53154844	-0.60788514
2.80434783	-0.69037026	-0.57779129	-0.52704433	-0.60466446
2.85217391	-0.68832683	-0.57529633	-0.52258209	-0.60139471
2.90000000	-0.68620553	-0.57276923	-0.51816935	-0.59808083
2.94782609	-0.68400898	-0.57021218	-0.51381284	-0.59472741
2.99565217	-0.68173972	-0.56762770	-0.50951864	-0.59133873

3.04347826	-0.67940021	-0.56501855	-0.50529240	-0.58791885
3.09130435	-0.67699287	-0.56238765	-0.50113950	-0.58447160
3.13913043	-0.67452008	-0.55973802	-0.49706519	-0.58100062
3.18695652	-0.67198424	-0.55707278	-0.49307474	-0.57750940
3.23478261	-0.66938774	-0.55439505	-0.48917352	-0.57400130
3.28260870	-0.66673302	-0.55170798	-0.48536703	-0.57047955
3.33043478	-0.66402252	-0.54901468	-0.48166092	-0.56694730
3.37826087	-0.66125876	-0.54631820	-0.47806088	-0.56340759
3.42608696	-0.65844427	-0.54362152	-0.47457237	-0.55986341
3.47391304	-0.65558163	-0.54092749	-0.47120026	-0.55631766
3.52173913	-0.65267347	-0.53823886	-0.46794816	-0.55277319
3.56956522	-0.64972245	-0.53555820	-0.46481769	-0.54923278
3.61739130	-0.64673126	-0.53288792	-0.46180768	-0.54569913
3.66521739	-0.64370264	-0.53023023	-0.45891350	-0.54217490
3.71304348	-0.64063931	-0.52758715	-0.45612686	-0.53866268
3.76086957	-0.63754402	-0.52496050	-0.45343613	-0.53516497
3.80869565	-0.63441953	-0.52235186	-0.45082725	-0.53168421
3.85652174	-0.63126857	-0.51976263	-0.44828501	-0.52822276
3.90434783	-0.62809387	-0.51719396	-0.44579427	-0.52478288
3.95217391	-0.62489811	-0.51464685	-0.44334111	-0.52136674
4.00000000	-0.62168396	-0.51212207	-0.44091344	-0.51797643

Table S8: Excited states of the H_2 molecule in a lineary polarized cavity with $\lambda = 0.05$ a.u. and $\omega = 0.466$ E_h and the polarization vector perpendicular to the molecular axis. States of *ungerade* parity. An unc-aug-pV5Z basis was used. Table contains all energies in units of E_h.

R in (a_0)	$\epsilon_\perp(B_{1u})$	$\epsilon_\perp(B_{1u})$	$\epsilon_\perp(B_{2u})$	$\epsilon_\perp(B_{3u})$	$\epsilon_\perp(B_{3u})$	$\epsilon_\perp(B_{3u})$
0.70000000	-0.26746090	-0.17632971	0.70000000	-0.24492874	-0.41776116	-0.24220182
0.74782609	-0.33167250	-0.23974924	0.74782609	-0.30842823	-0.47020883	-0.30546670
0.79565217	-0.38574306	-0.29311831	0.79565217	-0.36174054	-0.51287318	-0.35852038
0.84347826	-0.43154095	-0.33833669	0.84347826	-0.40673441	-0.54762626	-0.40322843
0.89130435	-0.47052476	-0.37687257	0.89130435	-0.44486924	-0.57592769	-0.44104653
0.93913043	-0.50384765	-0.40986768	0.93913043	-0.47729944	-0.59893020	-0.47312468
0.98695652	-0.53243194	-0.43822075	0.98695652	-0.50494867	-0.61755471	-0.50038132
1.03478261	-0.55702309	-0.46265070	1.03478261	-0.52856361	-0.63254478	-0.52355692
1.08260870	-0.57822924	-0.48374125	1.08260870	-0.54875348	-0.64450680	-0.54325334
1.13043478	-0.59655061	-0.50197235	1.13043478	-0.56601940	-0.65393994	-0.55996303
1.17826087	-0.61240168	-0.51774288	1.17826087	-0.58077667	-0.66125894	-0.57409105
1.22608696	-0.62612824	-0.53138731	1.22608696	-0.59337176	-0.66681151	-0.58597191
1.27391304	-0.63802051	-0.54318796	1.27391304	-0.60409557	-0.67089176	-0.59588262
1.32173913	-0.64832347	-0.55338434	1.32173913	-0.61319369	-0.67375056	-0.60405299
1.36956522	-0.65724483	-0.56218039	1.36956522	-0.62087454	-0.67560357	-0.61067392
1.41739130	-0.66496156	-0.56975024	1.41739130	-0.62731582	-0.67663712	-0.61590457
1.46521739	-0.67162496	-0.57624305	1.46521739	-0.63266967	-0.67701226	-0.61987870
1.51304348	-0.67736488	-0.58178693	1.51304348	-0.63706691	-0.67686716	-0.62271069
1.56086957	-0.68229311	-0.58649230	1.56086957	-0.64062045	-0.67631820	-0.62450132
1.60869565	-0.68650619	-0.59045472	1.60869565	-0.64342806	-0.67546020	-0.62534305
1.65652174	-0.69008764	-0.59375717	1.65652174	-0.64557471	-0.67436666	-0.62532444
1.70434783	-0.69310994	-0.59647205	1.70434783	-0.64713447	-0.67309084	-0.62453284
1.75217391	-0.69563608	-0.59866272	1.75217391	-0.64817209	-0.67166805	-0.62305528
1.80000000	-0.69772087	-0.60038497	1.80000000	-0.64874437	-0.67011928	-0.62097746
1.84782609	-0.69941212	-0.60168807	1.84782609	-0.64890123	-0.66845528	-0.61838189
1.89565217	-0.70075152	-0.60261579	1.89565217	-0.64868670	-0.66668061	-0.61534576
1.94347826	-0.70177548	-0.60320698	1.94347826	-0.64813966	-0.66479670	-0.61193938
1.99130435	-0.70251580	-0.60349602	1.99130435	-0.64729456	-0.66280412	-0.60822532
2.03913043	-0.70300023	-0.60351309	2.03913043	-0.64618197	-0.66070374	-0.60425828
2.08695652	-0.70325301	-0.60328457	2.08695652	-0.64482905	-0.65849738	-0.60008552
2.13478261	-	-	2.13478261	-0.64326001	-0.65618787	-0.59574756
2.18260870	-0.70314543	-0.60218006	2.18260870	-0.64149641	-0.65377902	-0.59127891
2.23043478	-0.70281963	-0.60134221	2.23043478	-0.63955755	-0.65127536	-0.58670898

2.27826087	-0.70233192	-0.60033593	2.27826087	-0.63746065	-0.64868194	-0.58206278
2.32608696	-0.70169457	-0.59917561	2.32608696	-0.63522116	-0.64600414	-0.57736160
2.37391304	-0.70091832	-0.59787426	2.37391304	-0.63285293	-0.64324749	-0.57262360
2.42173913	-0.70001257	-0.59644369	2.42173913	-0.63036843	-0.64041750	-0.56786425
2.46956522	-0.69898557	-0.59489465	2.46956522	-0.62777885	-0.63751963	-0.56309682
2.51739130	-0.69784455	-0.59323696	2.51739130	-0.62509432	-0.63455915	-0.55833263
2.56521739	-0.69659590	-0.59147963	2.56521739	-0.62232399	-0.63154116	-0.55358140
2.61304348	-0.69524527	-0.58963095	2.61304348	-0.61947614	-0.62847053	-0.54885145
2.66086957	-0.69379769	-0.58769856	2.66086957	-0.61655832	-0.62535187	-0.54414993
2.70869565	-0.69225766	-0.58568956	2.70869565	-0.61357739	-0.62218960	-0.53948294
2.75652174	-0.69062923	-0.58361057	2.75652174	-0.61053963	-0.61898787	-0.53485572
2.80434783	-0.68891611	-0.58146778	2.80434783	-0.60745083	-0.61575064	-0.53027274
2.85217391	-0.68712169	-0.57926699	2.85217391	-0.60431631	-0.61248165	-0.52573781
2.90000000	-0.68524912	-0.57701369	2.90000000	-0.60114100	-0.60918448	-0.52125414
2.94782609	-0.68330137	-0.57471306	2.94782609	-0.59792949	-0.60586252	-0.51682447
2.99565217	-0.68128125	-0.57237007	2.99565217	-0.59468609	-0.60251904	-0.51245107
3.04347826	-0.67919148	-0.56998943	3.04347826	-0.59141483	-0.59915717	-0.50813582
3.09130435	-0.67703468	-0.56757566	3.09130435	-0.58811954	-0.59577994	-0.50388026
3.13913043	-0.67481345	-0.56513311	3.13913043	-0.58480387	-0.59239027	-0.49968565
3.18695652	-0.67253035	-0.56266597	3.18695652	-0.58147129	-0.58899102	-0.49555295
3.23478261	-0.67018793	-0.56017826	3.23478261	-0.57812515	-0.58558497	-0.49148290
3.28260870	-0.66778876	-0.55767388	3.28260870	-0.57476868	-0.58217486	-0.48747604
3.33043478	-0.66533543	-0.55515658	3.33043478	-0.57140500	-0.57876335	-0.48353270
3.37826087	-0.66283054	-0.55262995	3.37826087	-0.56803716	-0.57535308	-0.47965307
3.42608696	-0.66027674	-0.55009747	3.42608696	-0.56466812	-0.57194666	-0.47583718
3.47391304	-0.65767672	-0.54756245	3.47391304	-0.56130077	-0.56854663	-0.47208495
3.52173913	-0.65503319	-0.54502805	3.52173913	-0.55793794	-0.56515552	-0.46839616
3.56956522	-0.65234890	-0.54249726	3.56956522	-0.55458239	-0.56177580	-0.46477051
3.61739130	-0.64962662	-0.53997292	3.61739130	-0.55123683	-0.55840992	-0.46120761
3.66521739	-0.64686915	-0.53745767	3.66521739	-0.54790388	-0.55506028	-0.45770699
3.71304348	-0.64407930	-0.53495398	3.71304348	-0.54458611	-0.55172920	-0.45426810
3.76086957	-0.64125989	-0.53246413	3.76086957	-0.54128604	-0.54841899	-0.45089035
3.80869565	-0.63841372	-0.52999020	3.80869565	-0.53800608	-0.54513186	-0.44757308
3.85652174	-0.63554360	-0.52753408	3.85652174	-0.53474858	-0.54186998	-0.44431561
3.90434783	-0.63265232	-0.52509747	3.90434783	-0.53151580	-0.53863541	-0.44111720
3.95217391	-0.62974262	-0.52268189	3.95217391	-0.52830991	-0.53543016	-0.43797708
4.00000000	-0.62681721	-0.52028868	4.00000000	-0.52513299	-0.53225612	-0.43489448

Table S9: Excited states of the H_2 molecule in an unpolarized cavity with $\lambda = 0.05$ a.u. and $\omega = 0.466$ E_h and the wave vector parallel to the molecular axis. States of *ungerade* parity. An unc-aug-cc-pV5Z basis was used. Table contains all energies in units of $E_{\rm h}$.

R in (a_0)	$k_{\parallel}(\Sigma_u^+)$	$k_{\parallel}(\Sigma_u^+)$	$k_{\parallel}(\Pi_u)$	$k_{\parallel}(\Pi_u)$
0.70000000	-0.26513901	-0.17393353	-0.41636890	-0.24005056
0.74782609	-0.32933273	-0.23724571	-0.46877792	-0.30328785
0.79565217	-0.38338572	-0.29050380	-0.51140294	-0.35631377
0.84347826	-0.42916600	-0.33561339	-0.54611601	-0.40099402
0.89130435	-0.46813188	-0.37404743	-0.57437670	-0.43878443
0.93913043	-0.50143633	-0.40695063	-0.59733770	-0.47083514
0.98695652	-0.53000164	-0.43522266	-0.61591984	-0.49806477
1.03478261	-0.55457331	-0.45958189	-0.63086656	-0.52121399
1.08260870	-0.57575957	-0.48061068	-0.64278407	-0.54088489
1.13043478	-0.59406071	-0.49878724	-0.65217137	-0.55757019
1.17826087	-0.60989131	-0.51450874	-0.65944295	-0.57167525
1.22608696	-0.62359723	-0.52810810	-0.66494621	-0.58353495
1.27391304	-0.63546879	-0.53986642	-0.66897493	-0.59342670
1.32173913	-0.64575100	-0.55002221	-0.67177962	-0.60158073
1.36956522	-0.65465165	-0.55877866	-0.67357557	-0.60818837
1.41739130	-0.66234772	-0.56630937	-0.67454880	-0.61340913
1.46521739	-0.66899057	-0.57276311	-0.67486022	-0.61737698
1.51304348	-0.67471006	-0.57826777	-0.67464808	-0.62020628
1.56086957	-0.67961802	-0.58293369	-0.67402920	-0.62199739
1.60869565	-0.68381097	-0.58685645	-0.67309923	-0.62284202
1.65652174	-0.68737243	-0.59011920	-0.67193282	-0.62282760
1.70434783	-0.69037487	-0.59279450	-0.67058455	-0.62204020
1.75217391	-0.69288123	-0.59494600	-0.66909105	-0.62056557
1.80000000	-0.69494631	-0.59662971	-0.66747433	-0.61848839
1.84782609	-0.69661786	-0.59789521	-0.66574587	-0.61589050
1.89565217	-0.69793754	-0.59878651	-0.66391052	-0.61284880
1.94347826	-0.69894172	-0.59934272	-0.66196974	-0.60943359
1.99130435	-0.69966216	-0.59959839	-0.65992388	-0.60570764
2.03913043	-0.70012659	-0.59958385	-0.65777353	-0.60172601
2.08695652	-0.70035920	-0.59932552	-0.65552011	-0.59753632
2.13478261	-0.70038111	-0.59884641	-0.65316611	-0.59317944
2.18260870	-0.70021077	-0.59816670	-0.65071499	-0.58869026
2.23043478	-0.69986425	-0.59730421	-0.64817098	-0.58409847
2.27826087	-0.69935561	-0.59627486	-0.64553889	-0.57942937

2.32608696	-0.69869712	-0.59509296	-0.64282388	-0.57470446
2.37391304	-0.69789954	-0.59377143	-0.64003127	-0.56994208
2.42173913	-0.69697225	-0.59232198	-0.63716645	-0.56515789
2.46956522	-0.69592351	-0.59075527	-0.63423475	-0.56036527
2.51739130	-0.69476055	-0.58908103	-0.63124134	-0.55557566
2.56521739	-0.69348978	-0.58730817	-0.62819123	-0.55079886
2.61304348	-0.69211685	-0.58544489	-0.62508922	-0.54604329
2.66086957	-0.69064681	-0.58349874	-0.62193989	-0.54131614
2.70869565	-0.68908417	-0.58147673	-0.61874758	-0.53662358
2.75652174	-0.68743300	-0.57938539	-0.61551642	-0.53197090
2.80434783	-0.68569703	-0.57723082	-0.61225034	-0.52736260
2.85217391	-0.68387965	-0.57501875	-0.60895305	-0.52280252
2.90000000	-0.68198405	-0.57275459	-0.60562810	-0.51829391
2.94782609	-0.68001321	-0.57044347	-0.60227889	-0.51383952
2.99565217	-0.67796995	-0.56809028	-0.59890865	-0.50944165
3.04347826	-0.67585703	-0.56569967	-0.59552052	-0.50510220
3.09130435	-0.67367707	-0.56327614	-0.59211750	-0.50082273
3.13913043	-0.67143271	-0.56082397	-0.58870253	-0.49660448
3.18695652	-0.66912650	-0.55834732	-0.58527844	-0.49244846
3.23478261	-0.66676104	-0.55585019	-0.58184803	-0.48835540
3.28260870	-0.66433890	-0.55333644	-0.57841401	-0.48432585
3.33043478	-0.66186270	-0.55080979	-0.57497906	-0.48036015
3.37826087	-0.65933505	-0.54827382	-0.57154582	-0.47645849
3.42608696	-0.65675864	-0.54573199	-0.56811688	-0.47262092
3.47391304	-0.65413615	-0.54318760	-0.56469480	-0.46884734
3.52173913	-0.65147032	-0.54064377	-0.56128210	-0.46513755
3.56956522	-0.64876392	-0.53810351	-0.55788127	-0.46149126
3.61739130	-0.64601974	-0.53556960	-0.55449473	-0.45790806
3.66521739	-0.64324058	-0.53304469	-0.55112490	-0.45438750
3.71304348	-0.64042928	-0.53053122	-0.54777411	-0.45092903
3.76086957	-0.63758866	-0.52803143	-0.54444465	-0.44753206
3.80869565	-0.63472154	-0.52554740	-0.54113875	-0.44419593
3.85652174	-0.63183075	-0.52308098	-0.53785856	-0.44091996
3.90434783	-0.62891908	-0.52063387	-0.53460615	-0.43770341
3.95217391	-0.62598928	-0.51820756	-0.53138353	-0.43454553
4.00000000	-0.62304407	-0.51580337	-0.52819260	-0.43144552

Table S10: Excited states of the H_2 molecule in an unpolarized cavity with $\lambda = 0.05$ a.u. and $\omega = 0.466$ E_h and the wave vector perpendicular to the molecular axis. States of *ungerade* parity. An unc-aug-cc-pV5Z basis was used. Table contains all energies in units of E_h.

R in (a_0)	$k_\perp(B_{1u})$	$k_\perp(B_{1u})$	$k_\perp(B_{1u})$	$k_\perp(B_{2u})$	$k_\perp(B_{2u})$	$k_\perp(B_{3u})$
0.70000000	-0.41664229	-0.26186856	-0.16970924	-0.41623591	-0.24019889	-0.24302814
0.74782609	-0.46913330	-0.32575759	-0.23294080	-0.46862501	-0.30341852	-0.30648066
0.79565217	-0.51186270	-0.37945847	-0.28613961	-0.51122859	-0.35642534	-0.35974401
0.84347826	-0.54670871	-0.42483089	-0.33120496	-0.54591866	-0.40108494	-0.40468690
0.89130435	-0.57513911	-0.46332246	-0.36960133	-0.57415482	-0.43885303	-0.44276871
0.93913043	-0.59831738	-0.49607221	-0.40246442	-0.59708973	-0.47087963	-0.47514384
0.98695652	-0.61717877	-0.52398383	-0.43068685	-0.61564424	-0.49808320	-0.50273590
1.03478261	-0.63248594	-0.54777825	-0.45498261	-0.63056183	-0.52120428	-0.52629157
1.08260870	-0.64487069	-0.56803150	-0.47593182	-0.64244875	-0.54084481	-0.54642002
1.13043478	-0.65486573	-0.58520219	-0.49401185	-0.65180405	-0.55749733	-0.56362236
1.17826087	-0.66292888	-0.59965212	-0.50961967	-0.65904230	-0.57156699	-0.57831384
1.22608696	-0.66946064	-0.61166290	-0.52308788	-0.66451103	-0.58338844	-0.59084091
1.27391304	-0.67481441	-0.62145232	-0.53469675	-0.66850414	-0.59323886	-0.60149442
1.32173913	-0.67929772	-0.62919382	-0.54468342	-0.67127233	-0.60134817	-0.61051994
1.36956522	-0.68316414	-0.63504096	-0.55324942	-0.67303106	-0.60790741	-0.61812583
1.41739130	-0.68660082	-0.63915303	-0.56056664	-0.67396655	-0.61307579	-0.62448974
1.46521739	-0.68972211	-0.64171195	-0.56678200	-0.67423982	-0.61698702	-0.62976378
1.51304348	-0.69257838	-0.64292197	-0.57202106	-0.67398917	-0.61975530	-0.63407873
1.56086957	-0.69517778	-0.64299488	-0.57639095	-0.67333129	-0.62148100	-0.63754742
1.60869565	-0.69750982	-0.64213227	-0.57998271	-0.67236152	-0.62225603	-0.64026760
1.65652174	-0.69956196	-0.64051380	-0.58287328	-0.67115400	-0.62216825	-0.64232419
1.70434783	-0.70132733	-0.63829370	-0.58512695	-0.66976262	-0.62130428	-0.64379122
1.75217391	-0.70280658	-0.63560270	-0.58679651	-0.66822330	-0.61975050	-0.64473341
1.80000000	-0.70400672	-0.63255245	-0.58792399	-0.66655738	-0.61759220	-0.64520752
1.84782609	-0.70493930	-0.62924055	-0.58854104	-0.66477583	-0.61491165	-0.64526347
1.89565217	-0.70561850	-0.62575510	-0.58866898	-0.66288315	-0.61178602	-0.64494523
1.94347826	-0.70605981	-0.62217837	-0.58831881	-0.66088062	-0.60828574	-0.64429170
1.99130435	-0.70627897	-0.61858884	-0.58749185	-0.65876855	-0.60447358	-0.64333728
2.03913043	-0.70629130	-0.61506071	-0.58618223	-0.65654754	-0.60040450	-0.64211254
2.08695652	-0.70611135	-0.61165973	-0.58438274	-0.65421912	-0.59612597	-0.64064463
2.13478261	-0.70575263	-0.60843554	-0.58209404	-0.65178587	-0.59167873	-0.63895772
2.18260870	-0.70522753	-0.60541343	-0.57933430	-0.64925138	-0.58709748	-0.63707339
2.23043478	-0.70454731	-0.60259076	-0.57614398	-0.64661999	-0.58241177	-0.63501089
2.27826087	-0.70372214	-0.59994154	-0.57258220	-0.64389659	-0.57764673	-0.63278745

2.32608696	-0.70276111	-0.59742686	-0.56871716	-0.64108643	-0.57282375	-0.63041849
2.37391304	-0.70167240	-0.59500541	-0.56461606	-0.63819490	-0.56796106	-0.62791786
2.42173913	-0.70046327	-0.59264049	-0.56033869	-0.63522745	-0.56307419	-0.62529799
2.46956522	-0.69914022	-0.59030277	-0.55593498	-0.63218945	-0.55817643	-0.62257008
2.51739130	-0.69770904	-0.58797050	-0.55144517	-0.62908610	-0.55327915	-0.61974424
2.56521739	-0.69617491	-0.58562834	-0.54690124	-0.62592246	-0.54839207	-0.61682959
2.61304348	-0.69454247	-0.58326601	-0.54232848	-0.62270335	-0.54352352	-0.61383443
2.66086957	-0.69281591	-0.58087698	-0.53774704	-0.61943334	-0.53868065	-0.61076628
2.70869565	-0.69099901	-0.57845751	-0.53317310	-0.61611682	-0.53386958	-0.60763199
2.75652174	-0.68909524	-0.57600582	-0.52861985	-0.61275793	-0.52909552	-0.60443786
2.80434783	-0.68710779	-0.57352156	-0.52409818	-0.60936059	-0.52436295	-0.60118964
2.85217391	-0.68503960	-0.57100540	-0.51961726	-0.60592854	-0.51967564	-0.59789266
2.90000000	-0.68289348	-0.56845873	-0.51518494	-0.60246534	-0.51503682	-0.59455186
2.94782609	-0.68067206	-0.56588349	-0.51080809	-0.59897440	-0.51044919	-0.59117183
2.99565217	-0.67837789	-0.56328197	-0.50649286	-0.59545897	-0.50591500	-0.58775687
3.04347826	-0.67601343	-0.56065677	-0.50224486	-0.59192219	-0.50143612	-0.58431104
3.09130435	-0.67358112	-0.55801066	-0.49806938	-0.58836709	-0.49701409	-0.58083817
3.13913043	-0.67108336	-0.55534655	-0.49397150	-0.58479661	-0.49265011	-0.57734191
3.18695652	-0.66852255	-0.55266747	-0.48995621	-0.58121362	-0.48834515	-0.57382577
3.23478261	-0.66590111	-0.54997646	-0.48602846	-0.57762092	-0.48409991	-0.57029309
3.28260870	-0.66322149	-0.54727660	-0.48219327	-0.57402126	-0.47991491	-0.56674713
3.33043478	-0.66048615	-0.54457093	-0.47845564	-0.57041733	-0.47579048	-0.56319104
3.37826087	-0.65769761	-0.54186247	-0.47482047	-0.56681180	-0.47172676	-0.55962787
3.42608696	-0.65485843	-0.53915415	-0.47129239	-0.56320727	-0.46772378	-0.55606061
3.47391304	-0.65197120	-0.53644878	-0.46787536	-0.55960634	-0.46378142	-0.55249218
3.52173913	-0.64903857	-0.53374906	-0.46457227	-0.55601155	-0.45989945	-0.54892543
3.56956522	-0.64606322	-0.53105753	-0.46138424	-0.55242540	-0.45607756	-0.54536313
3.61739130	-0.64304785	-0.52837654	-0.45831013	-0.54885036	-0.45231532	-0.54180802
3.66521739	-0.63999519	-0.52570829	-0.45534592	-0.54528885	-0.44861224	-0.53826274
3.71304348	-0.63690801	-0.52305473	-0.45248458	-0.54174322	-0.44496778	-0.53472989
3.76086957	-0.63378907	-0.52041764	-0.44971627	-0.53821579	-0.44138132	-0.53121198
3.80869565	-0.63064112	-0.51779857	-0.44702896	-0.53470879	-0.43785218	-0.52771146
3.85652174	-0.62746692	-0.51519885	-0.44440940	-0.53122440	-0.43437967	-0.52423068
3.90434783	-0.62426920	-0.51261964	-0.44184413	-0.52776471	-0.43096304	-0.52077192
3.95217391	-0.62105067	-0.51006186	-0.43932048	-0.52433172	-0.42760152	-0.51733735
4.00000000	-0.61781398	-0.50752628	-0.43682719	-0.52092734	-0.42429430	-0.51392905

Table S11: Excited states of the H_2 molecule in a lineary polarized cavity with $\lambda = 0.05$ a.u. and $\omega = 0.466$ E_h and the polarization vector parallel to the molecular axis. States of *gerade* parity. An unc-aug-pV5Z basis was used. Table contains all energies in units of E_h.

R in (a_0)	$\epsilon_\parallel(\Sigma_g^+)$	$\epsilon_\parallel(\Sigma_g^+)$	$\epsilon_\parallel(\Sigma_g^+)$	$\epsilon_\parallel(\Sigma_g^+)$	$\epsilon_\parallel(\Pi_g)$
0.70000000	-0.39096443	-0.27348739	-0.14367337	-0.11846410	-0.13436680
0.73333333	-0.42857705	-0.31845932	-0.18882317	-0.16378211	-0.18014830
0.76666667	-0.46111714	-0.35818255	-0.22877943	-0.20382659	-0.22067218
0.80000000	-0.48929307	-0.39336448	-0.26424748	-0.23929893	-0.25664491
0.83333333	-0.51369694	-0.42459714	-0.29581431	-0.27078608	-0.28865792
0.86666667	-0.53482692	-0.45237938	-0.32397165	-0.29878426	-0.31720968
0.90000000	-0.55310475	-0.47713407	-0.34913501	-0.32371642	-0.34272296
0.93333333	-0.56888940	-0.49922178	-0.37165850	-0.34594497	-0.36555840
0.96666667	-0.58248806	-0.51895149	-0.39184609	-0.36578165	-0.38602529
1.00000000	-0.59416486	-0.53658933	-0.40996017	-0.38349573	-0.40439034
1.03333333	-0.60414800	-0.55236541	-0.42622838	-0.39932082	-0.42088466
1.06666667	-0.61263552	-0.56647954	-0.44084904	-0.41346056	-0.43570941
1.10000000	-0.61980011	-0.57910566	-0.45399562	-0.42609327	-0.44904045
1.13333333	-0.62579317	-0.59039551	-0.46582043	-0.43737571	-0.46103210
1.16666667	-0.63074835	-0.60048140	-0.47645758	-0.44744611	-0.47182017
1.20000000	-0.63478497	-0.60947813	-0.48602547	-0.45642656	-0.48152459
1.23333333	-0.63801192	-0.61748346	-0.49462884	-0.46442492	-0.49025150
1.26666667	-0.64053387	-0.62457567	-0.50236053	-0.47153654	-0.49809504
1.30000000	-0.64246593	-0.63080196	-0.50930305	-0.47784576	-0.50513887
1.33333333	-0.64398409	-0.63613081	-0.51552989	-0.48342736	-0.51145748
1.36666667	-0.64552909	-0.64025025	-0.52110672	-0.48834786	-0.51711725
1.40000000	-0.64797916	-0.64239719	-0.52609237	-0.49266667	-0.52217751
1.43333333	-0.65113618	-0.64287282	-0.53053963	-0.49643706	-0.52669133
1.46666667	-0.65421932	-0.64255052	-0.53449599	-0.49970698	-0.53070628
1.50000000	-0.65698497	-0.64175708	-0.53800421	-0.50251976	-0.53426508
1.53333333	-0.65939085	-0.64060971	-0.54110286	-0.50491464	-0.53740618
1.56666667	-0.66144326	-0.63916973	-0.54382674	-0.50692728	-0.54016421
1.60000000	-0.66316153	-0.63747892	-0.54620734	-0.50859017	-0.54257048
1.63333333	-0.66456827	-0.63557002	-0.54827317	-0.50993297	-0.54465330
1.66666667	-0.66568620	-0.63347041	-0.55005009	-0.51098287	-0.54643833
1.70000000	-0.66653709	-0.63120388	-0.55156167	-0.51176481	-0.54794889
1.73333333	-0.66714134	-0.62879143	-0.55282942	-0.51230179	-0.54920617
1.76666667	-0.66751785	-0.62625185	-0.55387308	-0.51261509	-0.55022952
1.80000000	-0.66768411	-0.62360203	-0.55471086	-0.51272449	-0.55103658

1.83333333	-0.66765621	-0.62085723	-0.55535968	-0.51264851	-0.55164350
1.86666667	-0.66744900	-0.61803134	-0.55583537	-0.51240460	-0.55206510
1.90000000	-0.66707612	-0.61513695	-0.55615288	-0.51200941	-0.55231497
1.93333333	-0.66655015	-0.61218559	-0.55632646	-0.51147897	-0.55240565
1.96666667	-0.66588270	-0.60918776	-0.55636986	-0.51082899	-0.55234869
2.00000000	-0.66508447	-0.60615311	-0.55629649	-0.51007513	-0.55215478
2.03333333	-0.66416534	-0.60309047	-0.55611969	-0.50923332	-0.55183383
2.06666667	-0.66313446	-0.60000796	-0.55585290	-0.50832019	-0.55139505
2.10000000	-0.66200032	-0.59691303	-0.55550999	-0.50735356	-0.55084700
2.13333333	-0.66077077	-0.59381256	-0.55510554	-0.50635301	-0.55019770
2.16666667	-0.65945316	-0.59071290	-0.55465524	-0.50534067	-0.54945464
2.20000000	-0.65805429	-0.58761988	-0.55417622	-0.50434198	-0.54862484
2.23333333	-0.65658058	-0.58453895	-0.55368751	-0.50338652	-0.54771492
2.26666667	-0.65503800	-0.58147515	-0.55321040	-0.50250838	-0.54673111
2.30000000	-0.65343223	-0.57843327	-0.55276875	-0.50174565	-0.54567931
2.33333333	-0.65176862	-0.57541794	-0.55238908	-0.50113766	-0.54456510
2.36666667	-0.65005228	-0.57243390	-0.55210017	-0.50071885	-0.54339379
2.40000000	-0.64828811	-0.56948654	-0.55193197	-0.50050850	-0.54217045
2.43333333	-0.64648086	-0.56658311	-0.55191276	-0.50049891	-0.54089991
2.46666667	-0.64463516	-0.56373617	-0.55206341	-0.50064780	-0.53958680
2.50000000	-0.64275559	-0.56097498	-0.55238245	-0.50088160	-0.53823558
2.53333333	-0.64084673	-0.55839726	-0.55279054	-0.50111008	-0.53685051
2.56666667	-0.63891322	-0.55646780	-0.55282846	-0.50124556	-0.53543571
2.60000000	-0.63695986	-0.55627262	-0.55140118	-0.50121795	-0.53399518
2.63333333	-0.63499165	-0.55724494	-0.54905235	-0.50098169	-0.53253275
2.66666667	-0.63301394	-0.55862518	-0.54650702	-0.50051530	-0.53105214
2.70000000	-0.63103251	-0.56021020	-0.54392618	-0.49981661	-0.52955694
2.73333333	-0.62905375	-0.56191437	-0.54134980	-0.49889661	-0.52805062
2.76666667	-0.62708479	-0.56367879	-0.53879084	-0.49777389	-0.52653655
2.80000000	-0.62513375	-0.56545474	-0.53625384	-0.49647039	-0.52501795
2.83333333	-0.62320991	-0.56719963	-0.53374011	-0.49500866	-0.52349791
2.86666667	-0.62132402	-0.56887537	-0.53124944	-0.49341023	-0.52197941
2.90000000	-0.61948849	-0.57044735	-0.52878090	-0.49169478	-0.52046526
2.93333333	-0.61771765	-0.57188347	-0.52633321	-0.48987988	-0.51895813
2.96666667	-0.61602773	-0.57315348	-0.52390488	-0.48798097	-0.51746050
3.00000000	-0.61443670	-0.57422851	-0.52149441	-0.48601148	-0.51597469
3.03333333	-0.61296358	-0.57508129	-0.51910028	-0.48398308	-0.51450281
3.06666667	-0.61162711	-0.57568697	-0.51672106	-0.48190583	-0.51304674
3.10000000	-0.61044368	-0.57602481	-0.51435542	-0.47978844	-0.51160814

3.13333333	-0.60942471	-0.57608048	-0.51200213	-0.47763844	-0.51018843
3.16666667	-0.60857416	-0.57584835	-0.50966008	-0.47546235	-0.50878877
3.20000000	-0.60788679	-0.57533292	-0.50732832	-0.47326581	-0.50741005
3.23333333	-0.60734825	-0.57454862	-0.50500599	-0.47105375	-0.50605288
3.26666667	-0.60693683	-0.57351794	-0.50269239	-0.46883043	-0.50471761
3.30000000	-0.60662632	-0.57226837	-0.50038693	-0.46659959	-0.50340431
3.33333333	-0.60638918	-0.57082922	-0.49808911	-0.46436447	-0.50211279
3.36666667	-0.60619899	-0.56922906	-0.49579859	-0.46212794	-0.50084262
3.40000000	-0.60603203	-0.56749406	-0.49351509	-0.45989249	-0.49959313
3.43333333	-0.60586805	-0.56564721	-0.49123843	-0.45766032	-0.49836342
3.46666667	-0.60569030	-0.56370817	-0.48896853	-0.45543337	-0.49715244
3.50000000	-0.60548538	-0.56169348	-0.48670537	-0.45321335	-0.49595896
3.53333333	-0.60524276	-0.55961694	-0.48444901	-0.45100175	-0.49478163
3.56666667	-0.60495436	-0.55748999	-0.48219957	-0.44879991	-0.49361900
3.60000000	-0.60461411	-0.55532219	-0.47995721	-0.44660901	-0.49246955
3.63333333	-0.60421762	-0.55312150	-0.47772217	-0.44443008	-0.49133173
3.66666667	-0.60376178	-0.55089460	-0.47549470	-0.44226404	-0.49020397
3.70000000	-0.60324458	-0.54864717	-0.47327512	-0.44011172	-0.48908472
3.73333333	-0.60266487	-0.54638404	-0.47106376	-0.43797381	-0.48797246
3.76666667	-0.60202217	-0.54410934	-0.46886098	-0.43585096	-0.48686573
3.80000000	-0.60131657	-0.54182668	-0.46666718	-0.43374370	-0.48576315
3.83333333	-0.60054860	-0.53953919	-0.46448276	-0.43165252	-0.48466342
3.86666667	-0.59971913	-0.53724965	-0.46230815	-0.42957782	-0.48356531
3.90000000	-0.59882932	-0.53496048	-0.46014378	-0.42751996	-0.48246774
3.93333333	-0.59788058	-0.53267388	-0.45799010	-0.42547921	-0.48136968
3.96666667	-0.59687445	-0.53039181	-0.45584758	-0.42345582	-0.48027024
4.00000000	-0.59581264	-0.52811602	-0.45371666	-0.42144994	-0.47916864

Table S12: Excited states of the H_2 molecule in a lineary polarized cavity with $\lambda = 0.05$ a.u. and $\omega = 0.466$ E_h and the polarization vector perpendicular to the molecular axis. States of *gerade* parity. An unc-aug-pV5Z basis was used. Table contains all energies in units of E_h.

R in (a_0)	$\epsilon_\perp(A_g)$	$\epsilon_\perp(A_g)$	$\epsilon_\perp(A_g)$	$\epsilon_\perp(A_g)$	$\epsilon_\perp(B_{2g})$	$\epsilon_\perp(B_{3g})$
0.70000000	-0.39087730	-0.27399996	-0.14501300	-0.11571064	-0.00793795	-0.13383998
0.73333333	-0.42847558	-0.31898685	-0.19018665	-0.16103409	-0.05341178	-0.17963624
0.76666667	-0.46099957	-0.35872588	-0.23012982	-0.20108712	-0.09362673	-0.22017553
0.80000000	-0.48915753	-0.39392447	-0.26554909	-0.23657630	-0.12929026	-0.25616427
0.83333333	-0.51354139	-0.42517468	-0.29703566	-0.26809290	-0.16099457	-0.28819386
0.86666667	-0.53464916	-0.45297538	-0.32508771	-0.29613511	-0.18923875	-0.31676272
0.90000000	-0.55290241	-0.47774950	-0.35012781	-0.32112528	-0.21444606	-0.34229357
0.93333333	-0.56865991	-0.49985763	-0.37251662	-0.34342365	-0.23697751	-0.36514699
0.96666667	-0.58222864	-0.51960883	-0.39256368	-0.36333918	-0.25714262	-0.38563225
1.00000000	-0.59387252	-0.53726924	-0.41053591	-0.38113844	-0.27520813	-0.40401601
1.03333333	-0.60381949	-0.55306910	-0.42666453	-0.39705264	-0.29140495	-0.42052932
1.06666667	-0.61226730	-0.56720829	-0.44115058	-0.41128350	-0.30593378	-0.43537335
1.10000000	-0.61938830	-0.57986090	-0.45416952	-0.42400795	-0.31896979	-0.44872393
1.13333333	-0.62533352	-0.59117887	-0.46587500	-0.43538181	-0.33066637	-0.46073536
1.16666667	-0.63023614	-0.60129475	-0.47640201	-0.44554281	-0.34115830	-0.47154347
1.20000000	-0.63421500	-0.61032363	-0.48586945	-0.45461279	-0.35056439	-0.48126820
1.23333333	-0.63737856	-0.61836346	-0.49438234	-0.46269954	-0.35898967	-0.49001568
1.26666667	-0.63983204	-0.62549174	-0.50203358	-0.46989841	-0.36652728	-0.49788008
1.30000000	-0.64169704	-0.63174901	-0.50890564	-0.47629373	-0.37325998	-0.50494508
1.33333333	-0.64319741	-0.63705559	-0.51507192	-0.48196025	-0.37926156	-0.51128518
1.36666667	-0.64507760	-0.64079559	-0.52059792	-0.48696442	-0.38459789	-0.51696683
1.40000000	-0.64826986	-0.64215160	-0.52554230	-0.49136560	-0.38932797	-0.52204938
1.43333333	-0.65179213	-0.64220850	-0.52995772	-0.49521699	-0.39350472	-0.52658596
1.46666667	-0.65503346	-0.64166945	-0.53389152	-0.49856650	-0.39717573	-0.53062421
1.50000000	-0.65789175	-0.64071941	-0.53738637	-0.50145741	-0.40038384	-0.53420693
1.53333333	-0.66036539	-0.63943460	-0.54048076	-0.50392896	-0.40316776	-0.53737265
1.56666667	-0.66247441	-0.63786224	-0.54320951	-0.50601682	-0.40556247	-0.54015612
1.60000000	-0.66424375	-0.63603811	-0.54560410	-0.50775351	-0.40759965	-0.54258875
1.63333333	-0.66569873	-0.63399182	-0.54769312	-0.50916875	-0.40930806	-0.54469896
1.66666667	-0.66686350	-0.63174893	-0.54950256	-0.51028978	-0.41071382	-0.54651255
1.70000000	-0.66776066	-0.62933194	-0.55105611	-0.51114160	-0.41184070	-0.54805295
1.73333333	-0.66841110	-0.62676089	-0.55237548	-0.51174728	-0.41271038	-0.54934150
1.76666667	-0.66883409	-0.62405374	-0.55348058	-0.51212815	-0.41334262	-0.55039768
1.80000000	-0.66904731	-0.62122664	-0.55438983	-0.51230403	-0.41375550	-0.55123925

1.83333333	-0.66906703	-0.61829418	-0.55512033	-0.51229340	-0.41396553	-0.55188249
1.86666667	-0.66890820	-0.61526955	-0.55568807	-0.51211366	-0.41398786	-0.55234235
1.90000000	-0.66858454	-0.61216473	-0.55610814	-0.51178128	-0.41383637	-0.55263253
1.93333333	-0.66810869	-0.60899056	-0.55639490	-0.51131204	-0.41352377	-0.55276567
1.96666667	-0.66749229	-0.60575691	-0.55656218	-0.51072121	-0.41306179	-0.55275341
2.00000000	-0.66674606	-0.60247277	-0.55662347	-0.51002383	-0.41246115	-0.55260654
2.03333333	-0.66587989	-0.59914634	-0.55659209	-0.50923496	-0.41173178	-0.55233503
2.06666667	-0.66490294	-0.59578512	-0.55648140	-0.50837001	-0.41088276	-0.55194814
2.10000000	-0.66382368	-0.59239598	-0.55630500	-0.50744509	-0.40992251	-0.55145451
2.13333333	-0.66264995	-0.58898531	-0.55607694	-0.50647744	-0.40885877	-0.55086218
2.16666667	-0.66138905	-0.58555905	-0.55581193	-0.50548602	-0.40769868	-0.55017868
2.20000000	-0.66004779	-0.58212286	-0.55552549	-0.50449202	-0.40644885	-0.54941105
2.23333333	-0.65863251	-0.57868231	-0.55523409	-0.50351953	-0.40511538	-0.54856593
2.26666667	-0.65714915	-0.57524326	-0.55495506	-0.50259612	-0.40370391	-0.54764956
2.30000000	-0.65560331	-0.57181253	-0.55470608	-0.50175300	-0.40221968	-0.54666784
2.33333333	-0.65400027	-0.56839968	-0.55450359	-0.50102444	-0.40066752	-0.54562637
2.36666667	-0.65234506	-0.56502196	-0.55435764	-0.50044556	-0.39905193	-0.54453044
2.40000000	-0.65064248	-0.56172267	-0.55425311	-0.50004759	-0.39737709	-0.54338510
2.43333333	-0.64889715	-0.55866557	-0.55405441	-0.49985037	-0.39564687	-0.54219519
2.46666667	-0.64711356	-0.55661089	-0.55302799	-0.49985332	-0.39386488	-0.54096530
2.50000000	-0.64529614	-0.55629203	-0.55046367	-0.50002890	-0.39203449	-0.53969986
2.53333333	-0.64344926	-0.55675459	-0.54733320	-0.50032307	-0.39015883	-0.53840311
2.56666667	-0.64157736	-0.55756271	-0.54408162	-0.50066448	-0.38824084	-0.53707913
2.60000000	-0.63968495	-0.55862134	-0.54080320	-0.50097861	-0.38628325	-0.53573185
2.63333333	-0.63777674	-0.55989112	-0.53752606	-0.50120069	-0.38428863	-0.53436506
2.66666667	-0.63585768	-0.56133954	-0.53426166	-0.50128322	-0.38225939	-0.53298242
2.70000000	-0.63393315	-0.56293192	-0.53101584	-0.50119762	-0.38019780	-0.53158743
2.73333333	-0.63200900	-0.56463066	-0.52779211	-0.50093183	-0.37810598	-0.53018349
2.76666667	-0.63009177	-0.56639666	-0.52459283	-0.50048619	-0.37598595	-0.52877386
2.80000000	-0.62818889	-0.56819092	-0.52141976	-0.49986916	-0.37383961	-0.52736168
2.83333333	-0.62630885	-0.56997576	-0.51827431	-0.49909375	-0.37166875	-0.52594992
2.86666667	-0.62446152	-0.57171537	-0.51515771	-0.49817498	-0.36947509	-0.52454145
2.90000000	-0.62265841	-0.57337588	-0.51207111	-0.49712808	-0.36726024	-0.52313897
2.93333333	-0.62091297	-0.57492509	-0.50901570	-0.49596751	-0.36502578	-0.52174499
2.96666667	-0.61924075	-0.57633205	-0.50599279	-0.49470627	-0.36277317	-0.52036190
3.00000000	-0.61765949	-0.57756663	-0.50300409	-0.49335559	-0.36050384	-0.51899187
3.03333333	-0.61618872	-0.57859953	-0.50005197	-0.49192452	-0.35821916	-0.51763688
3.06666667	-0.61484883	-0.57940287	-0.49714015	-0.49041939	-0.35592046	-0.51629869
3.10000000	-0.61365930	-0.57995155	-0.49427507	-0.48884256	-0.35360901	-0.51497884

3.13333333	-0.61263613	-0.58022558	-0.49146892	-0.48718954	-0.35128605	-0.51367865
3.16666667	-0.61178876	-0.58021284	-0.48874669	-0.48544206	-0.34895278	-0.51239918
3.20000000	-0.61111748	-0.57991149	-0.48615943	-0.48355499	-0.34661038	-0.51114122
3.23333333	-0.61061231	-0.57933089	-0.48378560	-0.48145503	-0.34426000	-0.50990535
3.26666667	-0.61025394	-0.57849048	-0.48165733	-0.47911463	-0.34190276	-0.50869186
3.30000000	-0.61001658	-0.57741687	-0.47970062	-0.47661184	-0.33953977	-0.50750079
3.33333333	-0.60987137	-0.57614019	-0.47782770	-0.47403801	-0.33717209	-0.50633195
3.36666667	-0.60978968	-0.57469088	-0.47599086	-0.47144405	-0.33480081	-0.50518492
3.40000000	-0.60974524	-0.57309733	-0.47416926	-0.46885369	-0.33242696	-0.50405906
3.43333333	-0.60971530	-0.57138474	-0.47235410	-0.46627826	-0.33005159	-0.50295355
3.46666667	-0.60968098	-0.56957469	-0.47054191	-0.46372358	-0.32767571	-0.50186736
3.50000000	-0.60962710	-0.56768529	-0.46873159	-0.46119284	-0.32530034	-0.50079937
3.53333333	-0.60954171	-0.56573157	-0.46692319	-0.45868787	-0.32292647	-0.49974831
3.56666667	-0.60941565	-0.56372594	-0.46511733	-0.45620978	-0.32055509	-0.49871282
3.60000000	-0.60924202	-0.56167867	-0.46331492	-0.45375923	-0.31818716	-0.49769148
3.63333333	-0.60901576	-0.55959823	-0.46151696	-0.45133663	-0.31582366	-0.49668283
3.66666667	-0.60873329	-0.55749174	-0.45972453	-0.44894220	-0.31346551	-0.49568542
3.70000000	-0.60839224	-0.55536513	-0.45793868	-0.44657608	-0.31111367	-0.49469779
3.73333333	-0.60799117	-0.55322346	-0.45616043	-0.44423831	-0.30876904	-0.49371850
3.76666667	-0.60752940	-0.55107102	-0.45439077	-0.44192888	-0.30643252	-0.49274619
3.80000000	-0.60700685	-0.54891153	-0.45263063	-0.43964775	-0.30410501	-0.49177955
3.83333333	-0.60642393	-0.54674822	-0.45088088	-0.43739487	-0.30178738	-0.49081734
3.86666667	-0.60578142	-0.54458392	-0.44914235	-0.43517015	-0.29948046	-0.48985841
3.90000000	-0.60508042	-0.54242111	-0.44741580	-0.43297353	-0.29718510	-0.48890172
3.93333333	-0.60432227	-0.54026201	-0.44570191	-0.43080491	-0.29490210	-0.48794630
3.96666667	-0.60350849	-0.53810862	-0.44400134	-0.42866424	-0.29263224	-0.48699130
4.00000000	-0.60264075	-0.53596272	-0.44231467	-0.42655147	-0.29037630	-0.48603596

Table S13: Excited states of the H_2 molecule in an unpolarized cavity with $\lambda = 0.05$ a.u. and $\omega = 0.466$ E_h and the wave vector parallel to the molecular axis. States of *gerade* parity. An unc-aug-pV5Z basis was used. Table contains all energies in units of E_h.

R in (a_0)	$k_{\parallel}(\Sigma_g^+)$	$k_{\parallel}(\Sigma_g^+)$	$k_{\parallel}(\Sigma_g^+)$	$k_{\parallel}(\Sigma_g^+)$	$k_{\parallel}(\Delta_g)$	$k_{\parallel}(\Pi_g)$
0.70000000	-0.38949939	-0.38948100	-0.27078242	-0.14251710	-0.13757422	-0.00793795
0.73333333	-0.42707303	-0.42705176	-0.31574353	-0.18763423	-0.18335404	-0.05341178
0.76666667	-0.45957243	-0.45954778	-0.35545647	-0.22752002	-0.22387344	-0.09362673
0.80000000	-0.48770588	-0.48767726	-0.39062864	-0.26288139	-0.25983945	-0.12929026
0.83333333	-0.51206541	-0.51203211	-0.42185205	-0.29430994	-0.29184414	-0.16099457
0.86666667	-0.53314912	-0.53311026	-0.44962552	-0.32230419	-0.32038678	-0.18923875
0.90000000	-0.55137864	-0.55133318	-0.47437189	-0.34728709	-0.34589094	-0.21444606
0.93333333	-0.56711291	-0.56705952	-0.49645164	-0.36961965	-0.36871798	-0.23697751
0.96666667	-0.58065905	-0.58059609	-0.51617367	-0.38961170	-0.38917780	-0.25714262
1.00000000	-0.59228118	-0.59220654	-0.53380396	-0.40753042	-0.40753749	-0.27520813
1.03333333	-0.60220749	-0.60211847	-0.54957245	-0.42360724	-0.42402837	-0.29140495
1.06666667	-0.61063611	-0.61052912	-0.56367869	-0.43804332	-0.43885170	-0.30593378
1.10000000	-0.61773992	-0.61761007	-0.57629625	-0.45101423	-0.45218331	-0.31896979
1.13333333	-0.62367066	-0.62351114	-0.58757632	-0.46267370	-0.46417738	-0.33066637
1.16666667	-0.62856268	-0.62836352	-0.59765034	-0.47315677	-0.47496957	-0.34115830
1.20000000	-0.63253655	-0.63228247	-0.60663163	-0.48258241	-0.48467961	-0.35056439
1.23333333	-0.63570364	-0.63536953	-0.61461530	-0.49105566	-0.49341345	-0.35898967
1.26666667	-0.63817362	-0.63771437	-0.62167437	-0.49866952	-0.50126503	-0.36652728
1.30000000	-0.64007277	-0.63939640	-0.62784471	-0.50550648	-0.50831781	-0.37325998
1.33333333	-0.64160178	-0.64048606	-0.63306985	-0.51163994	-0.51464612	-0.37926156
1.36666667	-0.64321908	-0.64104601	-0.63701993	-0.51713543	-0.52031621	-0.38459789
1.40000000	-0.64564767	-0.64113208	-0.63908679	-0.52205158	-0.52538729	-0.38932797
1.43333333	-0.64869815	-0.64079413	-0.63956268	-0.52644098	-0.52991236	-0.39350472
1.46666667	-0.65171362	-0.64007677	-0.63919674	-0.53035092	-0.53393896	-0.39717573
1.50000000	-0.65444336	-0.63901995	-0.63832258	-0.53382398	-0.53750983	-0.40038384
1.53333333	-0.65683085	-0.63765953	-0.63707133	-0.53689857	-0.54066345	-0.40316776
1.56666667	-0.65887512	-0.63602775	-0.63551125	-0.53960938	-0.54343457	-0.40556247
1.60000000	-0.66059184	-0.63415363	-0.63368742	-0.54198778	-0.54585462	-0.40759965
1.63333333	-0.66200166	-0.63206329	-0.63163413	-0.54406223	-0.54795205	-0.40930806
1.66666667	-0.66312621	-0.62978032	-0.62937951	-0.54585859	-0.54975271	-0.41071382
1.70000000	-0.66398656	-0.62732601	-0.62694762	-0.54740043	-0.55128006	-0.41184070
1.73333333	-0.66460267	-0.62471961	-0.62435946	-0.54870929	-0.55255547	-0.41271038
1.76666667	-0.66499317	-0.62197856	-0.62163365	-0.54980497	-0.55359843	-0.41334262
1.80000000	-0.66517534	-0.61911861	-0.61878680	-0.55070575	-0.55442671	-0.41375550

1.83333333	-0.66516514	-0.61615408	-0.61583385	-0.55142858	-0.55505661	-0.41396553
1.86666667	-0.66497731	-0.61309792	-0.61278825	-0.55198935	-0.55550303	-0.41398786
1.90000000	-0.66462541	-0.60996192	-0.60966219	-0.55240302	-0.55577966	-0.41383637
1.93333333	-0.66412197	-0.60675675	-0.60646669	-0.55268384	-0.55589910	-0.41352377
1.96666667	-0.66347852	-0.60349213	-0.60321179	-0.55284553	-0.55587293	-0.41306179
2.00000000	-0.66270573	-0.60017688	-0.59990664	-0.55290148	-0.55571180	-0.41246115
2.03333333	-0.66181342	-0.59681902	-0.59655961	-0.55286492	-0.55542556	-0.41173178
2.06666667	-0.66081071	-0.59342585	-0.59317837	-0.55274911	-0.55502329	-0.41088276
2.10000000	-0.65970602	-0.59000400	-0.58977004	-0.55256754	-0.55451339	-0.40992251
2.13333333	-0.65850719	-0.58655950	-0.58634124	-0.55233413	-0.55390368	-0.40885877
2.16666667	-0.65722147	-0.58309784	-0.58289830	-0.55206338	-0.55320143	-0.40769868
2.20000000	-0.65585565	-0.57962399	-0.57944738	-0.55177050	-0.55241345	-0.40644885
2.23333333	-0.65441606	-0.57614250	-0.57599481	-0.55147145	-0.55154615	-0.40511538
2.26666667	-0.65290864	-0.57265749	-0.57254760	-0.55118263	-0.55060555	-0.40370391
2.30000000	-0.65133896	-0.56917268	-0.56911454	-0.55092008	-0.54959735	-0.40221968
2.33333333	-0.64971232	-0.56570867	-0.56569148	-0.55069701	-0.54852693	-0.40066752
2.36666667	-0.64803372	-0.56235392	-0.56221697	-0.55051706	-0.54739942	-0.39905193
2.40000000	-0.64630798	-0.55910637	-0.55875192	-0.55035253	-0.54621968	-0.39737709
2.43333333	-0.64453972	-0.55613612	-0.55529888	-0.55006157	-0.54499236	-0.39564687
2.46666667	-0.64273346	-0.55398966	-0.55186012	-0.54912425	-0.54372188	-0.39386488
2.50000000	-0.64089362	-0.55329519	-0.54843771	-0.54693525	-0.54241252	-0.39203449
2.53333333	-0.63902464	-0.55356167	-0.54503351	-0.54400235	-0.54106833	-0.39015883
2.56666667	-0.63713096	-0.55428874	-0.54164919	-0.54083421	-0.53969327	-0.38824084
2.60000000	-0.63521717	-0.55531237	-0.53828626	-0.53759311	-0.53829113	-0.38628325
2.63333333	-0.63328804	-0.55656713	-0.53494607	-0.53433220	-0.53686557	-0.38428863
2.66666667	-0.63134865	-0.55800943	-0.53162983	-0.53107314	-0.53542017	-0.38225939
2.70000000	-0.62940447	-0.55959910	-0.52833862	-0.52782655	-0.53395838	-0.38019780
2.73333333	-0.62746157	-0.56129552	-0.52507338	-0.52459846	-0.53248356	-0.37810598
2.76666667	-0.62552671	-0.56305786	-0.52183498	-0.52139273	-0.53099898	-0.37598595
2.80000000	-0.62360763	-0.56484604	-0.51862416	-0.51821208	-0.52950783	-0.37383961
2.83333333	-0.62171326	-0.56662160	-0.51544157	-0.51505860	-0.52801320	-0.37166875
2.86666667	-0.61985401	-0.56834808	-0.51228779	-0.51193406	-0.52651810	-0.36947509
2.90000000	-0.61804213	-0.56999095	-0.50916331	-0.50884009	-0.52502545	-0.36726024
2.93333333	-0.61629196	-0.57151721	-0.50606857	-0.50577836	-0.52353805	-0.36502578
2.96666667	-0.61462020	-0.57289490	-0.50300391	-0.50275076	-0.52205860	-0.36277317
3.00000000	-0.61304589	-0.57409275	-0.49996964	-0.49975960	-0.52058968	-0.36050384
3.03333333	-0.61158995	-0.57508021	-0.49696601	-0.49680810	-0.51913373	-0.35821916
3.06666667	-0.61027403	-0.57582827	-0.49399322	-0.49390097	-0.51769302	-0.35592046
3.10000000	-0.60911834	-0.57631121	-0.49105143	-0.49104563	-0.51626967	-0.35360901

3.13333333	-0.60813869	-0.57650934	-0.48825442	-0.48814074	-0.51486557	-0.35128605
3.16666667	-0.60734306	-0.57641209	-0.48554810	-0.48526125	-0.51348243	-0.34895278
3.20000000	-0.60672905	-0.57602039	-0.48295976	-0.48241300	-0.51212169	-0.34661038
3.23333333	-0.60628318	-0.57534716	-0.48053066	-0.47959599	-0.51078457	-0.34426000
3.26666667	-0.60598267	-0.57441541	-0.47828393	-0.47681023	-0.50947199	-0.34190276
3.30000000	-0.60579882	-0.57325465	-0.47619645	-0.47405566	-0.50818462	-0.33953977
3.33333333	-0.60570098	-0.57189693	-0.47421654	-0.47133225	-0.50692282	-0.33717209
3.36666667	-0.60565964	-0.57037355	-0.47229926	-0.46863990	-0.50568668	-0.33480081
3.40000000	-0.60564845	-0.56871306	-0.47041681	-0.46597852	-0.50447599	-0.33242696
3.43333333	-0.60564509	-0.56694025	-0.46855414	-0.46334799	-0.50329030	-0.33005159
3.46666667	-0.60563135	-0.56507608	-0.46670347	-0.46074819	-0.50212889	-0.32767571
3.50000000	-0.60559279	-0.56313794	-0.46486097	-0.45817898	-0.50099080	-0.32530034
3.53333333	-0.60551820	-0.56114017	-0.46302491	-0.45564020	-0.49987490	-0.32292647
3.56666667	-0.60539909	-0.55909453	-0.46119472	-0.45313169	-0.49877986	-0.32055509
3.60000000	-0.60522915	-0.55701072	-0.45937050	-0.45065327	-0.49770423	-0.31818716
3.63333333	-0.60500383	-0.55489674	-0.45755270	-0.44820476	-0.49664645	-0.31582366
3.66666667	-0.60471999	-0.55275928	-0.45574197	-0.44578597	-0.49560489	-0.31346551
3.70000000	-0.60437562	-0.55060396	-0.45393906	-0.44339669	-0.49457790	-0.31111367
3.73333333	-0.60396957	-0.54843553	-0.45214478	-0.44103674	-0.49356381	-0.30876904
3.76666667	-0.60350144	-0.54625806	-0.45035992	-0.43870588	-0.49256098	-0.30643252
3.80000000	-0.60297134	-0.54407508	-0.44858530	-0.43640391	-0.49156780	-0.30410501
3.83333333	-0.60237989	-0.54188964	-0.44682167	-0.43413061	-0.49058275	-0.30178738
3.86666667	-0.60172800	-0.53970443	-0.44506976	-0.43188574	-0.48960438	-0.29948046
3.90000000	-0.60101692	-0.53752183	-0.44333026	-0.42966909	-0.48863135	-0.29718510
3.93333333	-0.60024808	-0.53534397	-0.44160381	-0.42748040	-0.48766239	-0.29490210
3.96666667	-0.59942312	-0.53317275	-0.43989101	-0.42531945	-0.48669638	-0.29263224
4.00000000	-0.59854379	-0.53100988	-0.43819241	-0.42318600	-0.48573230	-0.29037630

Table S14: Excited states of the H_2 molecule in an unpolarized cavity with $\lambda = 0.05$ a.u. and $\omega = 0.466$ E_h and the wave vector perpendicular to the molecular axis. States of *gerade* parity. An unc-aug-pV5Z basis was used. Table contains all energies in units of E_h.

R in (a_0)	$k_\perp(A_g)$	$k_\perp(A_g)$	$k_\perp(A_g)$	$k_\perp(A_g)$	$k_\perp(A_g)$	$k_\perp(B_{2g})$	$k_\perp(B_{3g})$	$k_\perp(B_{3g})$
0.70000000	-0.38957704	-0.38935555	-0.27004845	-0.14113564	-0.11724357	-0.13757422	-0.13383998	0.15724594
0.73333333	-0.42716354	-0.42691372	-0.31499139	-0.18622078	-0.16262387	-0.18335404	-0.17963624	0.11090510
0.76666667	-0.45967728	-0.45939660	-0.35468535	-0.22611188	-0.20272772	-0.22387344	-0.22017553	0.06983876
0.80000000	-0.48782667	-0.48751243	-0.38983772	-0.26151545	-0.23825565	-0.25983945	-0.25616427	0.03334356
0.83333333	-0.51220380	-0.51185313	-0.42104052	-0.29301960	-0.26979389	-0.29184414	-0.28819386	0.00083011
0.86666667	-0.53330689	-0.53291671	-0.44879254	-0.32111688	-0.29783820	-0.32038678	-0.31676272	-0.02819903
0.90000000	-0.55155765	-0.55112470	-0.47351661	-0.34622328	-0.32281139	-0.34589094	-0.34229357	-0.05416639
0.93333333	-0.56731508	-0.56683585	-0.49557320	-0.36869307	-0.34507599	-0.36871798	-0.36514699	-0.07743310
0.96666667	-0.58088637	-0.58035708	-0.51527118	-0.38883016	-0.36494404	-0.38917780	-0.38563225	-0.09830982
1.00000000	-0.59253565	-0.59195222	-0.53287647	-0.40689677	-0.38268517	-0.40753749	-0.40401601	-0.11706569
1.03333333	-0.60249112	-0.60184909	-0.54861899	-0.42312031	-0.39853337	-0.42402837	-0.42052932	-0.13393552
1.06666667	-0.61095080	-0.61024525	-0.56269819	-0.43769883	-0.41269265	-0.43885170	-0.43537335	-0.14912555
1.10000000	-0.61808738	-0.61731273	-0.57528757	-0.45080559	-0.42534167	-0.45218331	-0.44872393	-0.16281799
1.13333333	-0.62405223	-0.62320199	-0.58653824	-0.46259268	-0.43663747	-0.46417738	-0.46073536	-0.17517452
1.16666667	-0.62897895	-0.62804521	-0.59658154	-0.47319405	-0.44671852	-0.47496957	-0.47154347	-0.18633899
1.20000000	-0.63298675	-0.63195923	-0.60553079	-0.48272802	-0.45570706	-0.48467961	-0.48126820	-0.19643963
1.23333333	-0.63618425	-0.63504812	-0.61348145	-0.49129924	-0.46371110	-0.49341345	-0.49001568	-0.20559070
	-	-	-	-	-	-0.50126503	-0.49788008	-0.21389401
1.30000000	-0.64057123	-0.63912066	-0.62665356	-0.50591435	-0.47713630	-0.50831781	-0.50494508	-0.22144020
1.33333333	-0.64202863	-0.64027838	-0.63188961	-0.51211417	-0.48271668	-0.51464612	-0.51128518	-0.22830991
1.36666667	-0.64337014	-0.64096952	-0.63599479	-0.51766563	-0.48763373	-0.52031621	-0.51696683	-0.23457496
1.40000000	-0.64524012	-0.64126919	-0.63842054	-0.52262749	-0.49194688	-0.52538729	-0.52204938	-0.24029939
1.43333333	-0.64786918	-0.64117717	-0.63908944	-0.52705251	-0.49570943	-0.52991236	-0.52658596	-0.24554053

1.46666667	-0.65067683	-0.64067484	-0.63873773	-0.53098811	-0.49896937	-0.53393896	-0.53062421	-0.25034992
1.50000000	-0.65328927	-0.63979827	-0.63782629	-0.53447700	-0.50177008	-0.53750983	-0.53420693	-0.25477428
1.53333333	-0.65559557	-0.63859757	-0.63652674	-0.53755768	-0.50415086	-0.54066345	-0.53737265	-0.25885628
1.56666667	-0.65757488	-0.63711575	-0.63491677	-0.54026491	-0.50614742	-0.54343457	-0.54015612	-0.26263540
1.60000000	-0.65923468	-0.63538836	-0.63304345	-0.54263009	-0.50779233	-0.54585462	-0.54258875	-0.26614858
1.63333333	-0.66059181	-0.63344559	-0.63094126	-0.54468169	-0.50911530	-0.54795205	-0.54469896	-0.26943094
1.66666667	-0.66166589	-0.63131363	-0.62863807	-0.54644550	-0.51014357	-0.54975271	-0.54651255	-0.27251639
1.70000000	-0.66247686	-0.62901564	-0.62615762	-0.54794502	-0.51090215	-0.55128006	-0.54805295	-0.27543826
1.73333333	-0.66304402	-0.62657227	-0.62352063	-0.54920170	-0.51141404	-0.55255547	-0.54934150	-0.27822981
1.76666667	-0.66338556	-0.62400208	-0.62074549	-0.55023521	-0.51170055	-0.55359843	-0.55039768	-0.28092475
1.80000000	-0.66351847	-0.62132180	-0.61784860	-0.55106370	-0.51178143	-0.55442671	-0.55123925	-0.28355759
1.83333333	-0.66345852	-0.61854660	-0.61484471	-0.55170402	-0.51167515	-0.55505661	-0.55188249	-0.28616386
1.86666667	-0.66322031	-0.61569027	-0.61174713	-0.55217194	-0.51139908	-0.55550303	-0.55234235	-0.28878001
1.90000000	-0.66281733	-0.61276535	-0.60856788	-0.55248234	-0.51096971	-0.55577966	-0.55263253	-0.29144296
1.93333333	-0.66226203	-0.60978329	-0.60531787	-0.55264942	-0.51040287	-0.55589910	-0.55276567	-0.29418905
1.96666667	-0.66156590	-0.60675456	-0.60200697	-0.55268688	-0.50971399	-0.55587293	-0.55275341	-0.29705236
2.00000000	-0.66073959	-0.60368872	-0.59864419	-0.55260808	-0.50891832	-0.55571180	-0.55260654	-0.30006246
2.03333333	-0.65979293	-0.60059458	-0.59523770	-0.55242630	-0.50803127	-0.55542556	-0.55233503	-0.30324181
2.06666667	-0.65873502	-0.59748018	-0.59179499	-0.55215494	-0.50706875	-0.55502329	-0.55194814	-0.30660324
2.10000000	-0.65757430	-0.59435293	-0.58832290	-0.55180779	-0.50604762	-0.55451339	-0.55145451	-0.31014823
2.13333333	-0.65631862	-0.59121964	-0.58482773	-0.55139936	-0.50498623	-0.55390368	-0.55086218	-0.31386653
2.16666667	-0.65497527	-0.58808659	-0.58131531	-0.55094515	-0.50390509	-0.55320143	-0.55017868	-0.31773732
2.20000000	-0.65355108	-0.58495955	-0.57779114	-0.55046203	-0.50282759	-0.55241345	-0.54941105	-0.32173160
2.23333333	-0.65205240	-0.58184388	-0.57426050	-0.54996853	-0.50178077	-0.55154615	-0.54856593	-0.32581539
2.26666667	-0.65048524	-0.57874452	-0.57072875	-0.54948499	-0.50079588	-0.55060555	-0.54764956	-0.32995279
2.30000000	-0.64885525	-0.57566613	-0.56720173	-0.54903348	-0.49990811	-0.54959735	-0.54666784	-0.33410863
2.33333333	-0.64716777	-0.57261318	-0.56368689	-0.54863679	-0.49915478	-0.54852693	-0.54562637	-0.33825021
2.36666667	-0.64542791	-0.56959017	-0.56019606	-0.54831536	-0.49857048	-0.54739942	-0.54453044	-0.34234845

2.40000000	-0.64364059	-0.56660203	-0.55675489	-0.54807665	-0.49817849	-0.54621968	-0.54338510	-0.34637822
2.43333333	-0.64181057	-0.56365523	-0.55344614	-0.54786938	-0.49797950	-0.54499236	-0.54219519	-0.35031836
2.46666667	-0.63994248	-0.56076051	-0.55068858	-0.54729929	-0.49794249	-0.54372188	-0.54096530	-0.35415142
2.50000000	-0.63804094	-0.55794246	-0.54966526	-0.54518791	-0.49800465	-0.54241252	-0.53969986	-0.35786324
2.53333333	-0.63611058	-0.55528336	-0.54978223	-0.54206621	-0.49808284	-0.54106833	-0.53840311	-0.36144249
2.56666667	-0.63415607	-0.55320830	-0.54981811	-0.53873671	-0.49809186	-0.53969327	-0.53707913	-0.36488026
2.60000000	-0.63218230	-0.55292934	-0.54838911	-0.53536261	-0.49796060	-0.53829113	-0.53573185	-0.36816964
2.63333333	-0.63019437	-0.55390113	-0.54597813	-0.53198380	-0.49764067	-0.53686557	-0.53436506	-0.37130540
2.66666667	-0.62819775	-0.55528223	-0.54337839	-0.52861463	-0.49710722	-0.53542017	-0.53298242	-0.37428367
2.70000000	-0.62619841	-0.55686513	-0.54074943	-0.52526177	-0.49635484	-0.53395838	-0.53158743	-0.37710170
2.73333333	-0.62420297	-0.55856419	-0.53812841	-0.52192898	-0.49539167	-0.53248356	-0.53018349	-0.37975769
2.76666667	-0.62221889	-0.56032030	-0.53552699	-0.51861863	-0.49423394	-0.53099898	-0.52877386	-0.38225057
2.80000000	-0.62025468	-0.56208446	-0.53294916	-0.51533236	-0.49290167	-0.52950783	-0.52736168	-0.38457993
2.83333333	-0.61832020	-0.56381365	-0.53039595	-0.51207141	-0.49141588	-0.52801320	-0.52594992	-0.38674588
2.86666667	-0.61642689	-0.56546928	-0.52786703	-0.50883673	-0.48979686	-0.52651810	-0.52454145	-0.38874898
2.90000000	-0.61458807	-0.56701597	-0.52536142	-0.50562906	-0.48806338	-0.52502545	-0.52313897	-0.39059020
2.93333333	-0.61281917	-0.56842070	-0.52287782	-0.50244900	-0.48623225	-0.52353805	-0.52174499	-0.39227085
2.96666667	-0.61113776	-0.56965202	-0.52041475	-0.49929706	-0.48431835	-0.52205860	-0.52036190	-0.39379253
3.00000000	-0.60956322	-0.57067978	-0.51797067	-0.49617362	-0.48233468	-0.52058968	-0.51899187	-0.39515718
3.03333333	-0.60811595	-0.57147543	-0.51554408	-0.49307902	-0.48029255	-0.51913373	-0.51763688	-0.39636699
3.06666667	-0.60681567	-0.57201324	-0.51313352	-0.49001353	-0.47820178	-0.51769302	-0.51629869	-0.39742442
3.10000000	-0.60567897	-0.57227234	-0.51073764	-0.48697734	-0.47607088	-0.51626967	-0.51497884	-0.39833220
3.13333333	-0.60471631	-0.57223945	-0.50835518	-0.48397063	-0.47390722	-0.51486557	-0.51367865	-0.39909332
3.16666667	-0.60392933	-0.57191129	-0.50598501	-0.48099352	-0.47171718	-0.51348243	-0.51239918	-0.39971105
3.20000000	-0.60330945	-0.57129575	-0.50362613	-0.47804612	-0.46950633	-0.51212169	-0.51114122	-0.40018888
3.23333333	-0.60283858	-0.57041109	-0.50127766	-0.47512849	-0.46727949	-0.51078457	-0.50990535	-0.40053057
3.26666667	-0.60249156	-0.56928323	-0.49893884	-0.47224069	-0.46504083	-0.50947199	-0.50869186	-0.40074012
3.30000000	-0.60223969	-0.56794224	-0.49660904	-0.46938277	-0.46279400	-0.50818462	-0.50750079	-0.40082176

3.33333333	-0.60205392	-0.56641895	-0.49428775	-0.46655477	-0.46054216	-0.50692282	-0.50633195	-0.40077995
3.36666667	-0.60190731	-0.56474248	-0.49197455	-0.46375673	-0.45828804	-0.50568668	-0.50518492	-0.40061930
3.40000000	-0.60177629	-0.56293889	-0.48966914	-0.46098871	-0.45603398	-0.50447599	-0.50405906	-0.40034463
3.43333333	-0.60164112	-0.56103066	-0.48737131	-0.45825082	-0.45378198	-0.50329030	-0.50295355	-0.39996089
3.46666667	-0.60148582	-0.55903674	-0.48508092	-0.45554319	-0.45153371	-0.50212889	-0.50186736	-0.39947314
3.50000000	-0.60129778	-0.55697288	-0.48279794	-0.45286609	-0.44929049	-0.50099080	-0.50079937	-0.39888651
3.53333333	-0.60106722	-0.55485215	-0.48052238	-0.45021988	-0.44705332	-0.49987490	-0.49974831	-0.39820619
3.56666667	-0.60078676	-0.55268532	-0.47825433	-0.44760517	-0.44482275	-0.49877986	-0.49871282	-0.39743736
3.60000000	-0.60045095	-0.55048135	-0.47599393	-0.44502291	-0.44259883	-0.49770423	-0.49769148	-0.39658519
3.63333333	-0.60005590	-0.54824770	-0.47374138	-0.44247464	-0.44038091	-0.49664645	-0.49668283	-0.39565478
3.66666667	-0.59959898	-0.54599064	-0.47149692	-0.43996277	-0.43816728	-0.49560489	-0.49568542	-0.39465117
3.70000000	-0.59907854	-0.54371545	-0.46926084	-0.43749116	-0.43595470	-0.49457790	-0.49469779	-0.39357925
3.73333333	-0.59849375	-0.54142668	-0.46703344	-0.43506561	-0.43373787	-0.49356381	-0.49371850	-0.39244380
3.76666667	-0.59784440	-0.53912821	-0.46481506	-0.43269379	-0.43150955	-0.49256098	-0.49274619	-0.39124943
3.80000000	-0.59713081	-0.53682341	-0.46260609	-0.43038288	-0.42926284	-0.49156780	-0.49177955	-0.39000060
3.83333333	-0.59635369	-0.53451526	-0.46040690	-0.42813487	-0.42699603	-0.49058275	-0.49081734	-0.38870155
3.86666667	-0.59551411	-0.53220636	-0.45821791	-0.42594393	-0.42471511	-0.48960438	-0.48985841	-0.38735637
3.90000000	-0.59461335	-0.52989902	-0.45603954	-0.42379992	-0.42243031	-0.48863135	-0.48890172	-0.38596893
3.93333333	-0.59365293	-0.52759532	-0.45387222	-0.42169333	-0.42015117	-0.48766239	-0.48794630	-0.38454290
3.96666667	-0.59263451	-0.52529712	-0.45171639	-0.41961734	-0.41788450	-0.48669638	-0.48699130	-0.38308176
4.00000000	-0.59155990	-0.52300609	-0.44957251	-0.41756749	-0.41563465	-0.48573230	-0.48603596	-0.38158880
